# Computerized clinical decision support systems for drug prescribing and management: A decision-maker-researcher partnership systematic review

**DOI:** 10.1186/1748-5908-6-89

**Published:** 2011-08-03

**Authors:** Brian J Hemens, Anne Holbrook, Marita Tonkin, Jean A Mackay, Lorraine Weise-Kelly, Tamara Navarro, Nancy L Wilczynski, R Brian Haynes

**Affiliations:** 1 Health Information Research Unit, Department of Clinical Epidemiology and Biostatistics, McMaster University, 1280 Main Street West, Hamilton, ON, Canada; 2 Department of Medicine, McMaster University, 1280 Main Street West, Hamilton, ON, Canada; 3 Department of Clinical Epidemiology and Biostatistics, McMaster University, 1280 Main Street West, Hamilton, ON, Canada; 4 Hamilton Health Sciences, 1200 Main Street West, Hamilton, ON, Canada

## Abstract

**Background:**

Computerized clinical decision support systems (CCDSSs) for drug therapy management are designed to promote safe and effective medication use. Evidence documenting the effectiveness of CCDSSs for improving drug therapy is necessary for informed adoption decisions. The objective of this review was to systematically review randomized controlled trials assessing the effects of CCDSSs for drug therapy management on process of care and patient outcomes. We also sought to identify system and study characteristics that predicted benefit.

**Methods:**

We conducted a decision-maker-researcher partnership systematic review. We updated our earlier reviews (1998, 2005) by searching MEDLINE, EMBASE, EBM Reviews, Inspec, and other databases, and consulting reference lists through January 2010. Authors of 82% of included studies confirmed or supplemented extracted data. We included only randomized controlled trials that evaluated the effect on process of care or patient outcomes of a CCDSS for drug therapy management compared to care provided without a CCDSS. A study was considered to have a positive effect (*i.e.*, CCDSS showed improvement) if at least 50% of the relevant study outcomes were statistically significantly positive.

**Results:**

Sixty-five studies met our inclusion criteria, including 41 new studies since our previous review. Methodological quality was generally high and unchanged with time. CCDSSs improved process of care performance in 37 of the 59 studies assessing this type of outcome (64%, 57% of all studies). Twenty-nine trials assessed patient outcomes, of which six trials (21%, 9% of all trials) reported improvements.

**Conclusions:**

CCDSSs inconsistently improved process of care measures and seldomly improved patient outcomes. Lack of clear patient benefit and lack of data on harms and costs preclude a recommendation to adopt CCDSSs for drug therapy management.

## Background

Computerized clinical decision support systems (CCDSSs) algorithmically apply an electronic knowledge base to individual patient data to generate and present suggested actions intended to enhance health and healthcare [1-3]. CCDSSs for drug therapy management are used to facilitate evidence-informed medication use [4], reduce the incidence of harmful medication errors [5], and improve healthcare system efficiency [2,4,6]. In this review, we considered any CCDSS that provides recommendations to healthcare providers regarding the initiation, modification, monitoring, or discontinuation of drug therapy, based on the patient's characteristics. Systems designed solely to provide advice on the management of narrow therapeutic index drugs based on *in vivo *monitoring and pharmacokinetic principles (therapeutic drug monitoring [7]) were excluded because they are in a complementary in-depth review on therapeutic drug monitoring and dosing (submitted to *Implementation Science*). Variety in the structure and function of CCDSSs complicates methodologically sound investigations and comparisons of these interventions. A CCDSS may be integrated with one or more of electronic medical records (EMR), computerized provider order entry systems (CPOE) or electronic transmission of prescriptions to the point of dispensing. CCDSSs require input of patient data to deliver advice, and this may be accomplished via integration with patient information repositories or by manual entry. Optimally, the knowledge base of a CCDSS used to generate recommendations is evidence-informed, though this may not always be the case. Advice may be delivered to many kinds of providers through a variety of media across diverse settings of care. Systems may be developed 'in house' to meet the requirements of a specific organization or acquired from a commercial vendor.

Decision makers, clinicians, and patients should require sound evidence of CCDSS benefits, risks and costs prior to general adoption, as for any health intervention. Randomized controlled trials (RCTs) represent the gold standard for unbiased comparisons of alternative interventions [8].

Our previous review [2] included 24 RCTs of a CCDSS for drug therapy. Of the 13 trials measuring patient-important outcomes, only one detected benefit with a CCDSS. Small study size limited detection of change in patient-important clinical endpoints.

Lack of data on which to base overall conclusions on the effects of CCDSSs together with the increased pace of research in the field prompted this update of our previous review. To aid decision makers and providers, we evaluated the effects of CCDSSs on process of care and patient outcomes via cumulative synthesis of relevant RCTs. This review is one of a series of reviews considering the effects of CCDSSs across multiple application areas (therapeutic drug monitoring and dosing, primary preventive care, diagnostic test ordering, acute care management, and chronic disease management).

## Methods

This review was conducted in accord with a published protocol http://www.implementationscience.com/content/5/1/12[9]. Some trials have been included in more than one review because they were relevant to more than one CCDSS intervention area. Specific details for the drug therapy review follow.

### Research questions

For this review, we were primarily interested in determining: 1) Do CCDSSs improve performance on drug-related process of care measures or patient outcomes compared to usual care? 2) What features or characteristics of studies or systems are associated with improved process or patient measures? Based partly on our previous review [2], we expected studies demonstrating benefit from CCDSSs would: a) be integrated with an existing EMR or CPOE system (versus a standalone system); b) deliver decision support before or during a patient care encounter where the decision that is being supported was taken (versus supply of decision support at any other time); c) actively suggest treatments or other actions (versus supply general information or access to general information); d) be used in a patient care setting affiliated with an academic institution (versus any other setting); e) have developers of the CCDSS who were also the study investigators (versus study investigators not associated with developers); f) measure intermediate/surrogate patient outcomes (versus patient-important outcomes); g) describe higher rates of user satisfaction (versus no or low rates of user satisfaction).

### Partnering with decision makers

This review was conducted in partnership with senior hospital managers and clinical leaders with an academic research team in the field of knowledge translation, from healthcare research to clinical practice. Decision makers provided key input as to the kind of data needed about CCDSS to drive effective choices and these needs were incorporated into the research plan where feasible.

### Search strategy

The search methods employed have been described in detail elsewhere [9]. Briefly, a comprehensive search (2004 to 2010) of major biomedical databases (MEDLINE, EMBASE, Ovid's EBM Reviews, and Inspec) yielded citations for screening. Pairs of reviewers independently evaluated each citation and abstract. A third reader resolved disagreements where necessary. Inter-reviewer agreement on study eligibility was measured via unweighted Cohen's kappa (κ). Studies from our previous reviews were carried forward to this review if they met the inclusion criteria, effectively extending our search from database inception to 2010.

### Study selection

Studies were included for review if they described an RCT comparing outcomes for a group of providers or patients using a CCDSS compared with care without the CCDSS. Non-experimental or quasi-experimental investigations were excluded. For inclusion, we required that independent providers or post-graduate trainee (*e.g. *medical residents) providers be identified as primary users of the CCDSS. The intervention CCDSS was required to provide patient-specific output in the form of assessments, management options, or recommendations to the clinical user. Studies were excluded if the system was used solely by students, only provided summaries of information for patients, only provided feedback on groups of patients without feedback about individual patients, only provided computer-aided instruction, or were used for image analysis. The six CCDSS intervention areas in this series of reviews used a common eligibility screening process [9] to identify reports of trials of CCDSSs for any purpose. Studies were then further screened to determine if the system provided advice regarding drug therapy.

### Data extraction

Independent reviewers extracted key data concerning study methods, CCDSS and population characteristics, possible sources of bias, and outcomes in duplicate. Primary authors of each study were asked to review the extracted data for their study and offer comments on the extracted data.

### Assessment of study quality

Included studies were evaluated on five dimensions of quality--including concealment of allocation, appropriate unit of allocation, appropriate adjustment for baseline differences, adequate follow-up, and appropriate outcome assessment--to yield a 10-point methods score [9].

### Assessment of CCDSS intervention effects

#### Outcome selection and improvement determinations

Each included trial describing a CCDSS that provided advice exclusively or predominantly about drug therapy was classified as drug therapy management only (Rx-only). Systems that gave advice on drug therapy as part of a more complex intervention were categorised as 'multi-faceted' CCDSSs. Improvement was considered to have occurred where 50% or more of the selected outcomes showed a benefit with a CCDSS compared to control. To determine whether improvement occurred, all outcomes were selected from the first of: primary, then pre-specified, then any outcome(s), as defined by study authors (*i.e.*, if a primary outcome was reported for a trial this was used to determine improvement to the exclusion of any other reported outcomes). Where no outcomes were defined as primary, but the study reported a sample size calculation for an outcome, we defined that outcome as primary. These criteria are more specific than those used in our previous review [2]; therefore, the assignment of effect was adjusted for some studies included in the 2005 review. Process of care outcomes for multi-faceted CCDSS studies were selected only if they were clearly drug-related. Multi-faceted systems that reported a patient outcome but did not report a drug-related process of care outcome intermediary were excluded as non-responsive to our research questions. Where there were multiple intervention arms, the arm testing the most sophisticated CCDSS was used to determine improvement. Two reviewers, working independently and blinded to study results, classified trials as drug treatment-only or multi-faceted, and initially identified the outcomes used to determine improvement, with disagreements resolved by consensus.

#### Data synthesis and analysis

Data were summarized using descriptive summary measures, including proportions for categorical variables and means (± SD) for continuous variables. For interpretation, a 2-sided *p *< 0.05 indicated statistical significance. For individual studies we report the measures of association and *p *-values reported in the studies.

We did not attempt a meta-analysis because of differences across studies of participants, settings, disease conditions, interventions, and outcomes. Tests of association between study and CCDSS factors and improved outcomes were tested using the univariate Fisher's exact test. Multivariate analyses were conducted using multinomial logistic regression. All analyses were conducted using SPSS v. 17.

Sensitivity analyses were conducted to determine if the class of outcome selected to judge improvement affected our results. We also identified cluster randomized trials where units of allocation and units of analysis were appropriately matched or mismatched. The proportions of successful trials with matched versus mismatched units were compared.

## Results

**Figure 1:**
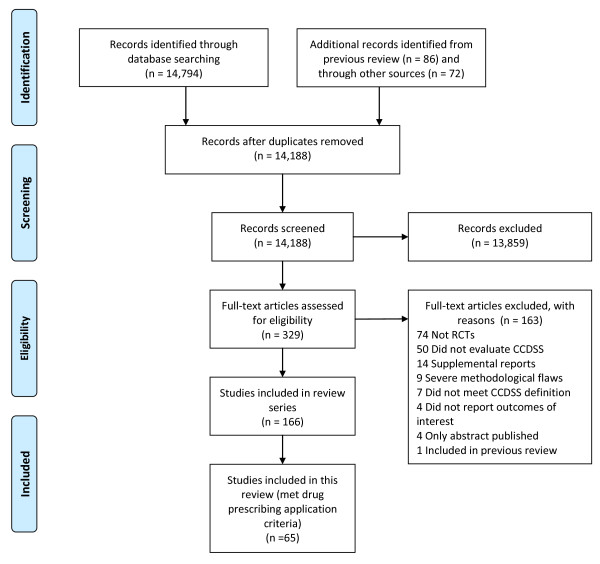
Flow diagram of included and excluded studies for the update 1 January 2004 to 6 January 2010 with specifics for drug prescribing and management*. *Details provided in: Haynes RB *et al. *[9]. Two updating searches were performed, for 2004 to 2009 and to 6 January 2010 and the results of the search process are consolidated here.

A total of 14,952 possibly relevant records were identified [9]. After excluding duplicate records, 14,188 records were screened to yield 329 articles eligible for full-text screening. Of those, 166 trials met our criteria for a CCDSS; Cohen's κ for reviewer agreement on trial eligibility was 0.93 (95% confidence interval [CI], 0.91 to 0.94). Initially, 71 trials were judged relevant to drug therapy management. Six of these trials [10-15] were excluded because they studied a multi-faceted CCDSS that included drug therapy, but did not report any drug-related process outcomes. A total of 65 trials reported in 74 papers were included [16-89] (see Figure 1). Twenty-four RCTs [60-62,65-67,69,71-76,78,80-89] had been included in the previous version of our review [2]. Study authors confirmed or supplemented our data extraction for 53 of 65 included studies (82%) [16-20,23,25-33,35-39,42-45,47-49,53-55,57,58,60-62,65-72,74-76,78,81,83-86,88]. Forty-seven included studies contribute outcomes to this review as well as other CCDSS interventions in the series; four studies [49,56,76,80] to four reviews, 16 studies [16,19,21,28,40,44,45,53,55,59,62,64,68,69,74,77-79,82,85,89] to three reviews, and 27 studies [20,22,23,26,27,29,31,32,34,35,39,41-43,46-48,50,52,54,60,63,66,70,72,75,81,86-88] to two reviews; but we focused here on drug prescribing-relevant outcomes.

Summary of trial quality is reported in Additional file 1, Table S1; system characteristics in Additional file 2, Table S2; study characteristics in Additional file 3, Table S3; outcome data in Additional file 4, Table S4 and Table 1, and other CCDSS-related outcomes in Additional file 5, Table S5.

**Table 1:** Summary of results for CCDSS trials of drug prescribing

Study	Methods score	Indication	No. of centres/providers/patients	Process of care outcomes	CCDSS Effect^a^	Patient outcomes	CCDSS Effect^a^
**Studies of drug-only interventions**							

Field, 2009[17,24]	7	Alerts to promote appropriate drug prescribing and monitoring for patients with renal insufficiency in long-term care.	1*/10/833	Appropriate final drug orders.	**0**	...	**..**.

Fortuna, 2009[18]	10	Alerts to consider cost when prescribing hypnotics for adults in primary and urgent care.	14*/257/...	Change in hypnotic drug prescriptions.	**+**	...	**..**.

Lo, 2009[20]	10	Alerts to order laboratory tests when prescribing new medications in primary care.	22*/366/2765	Ordering appropriate baseline laboratory tests.	**0**	...	**..**.

Terrell, 2009[23]	9	Alerts to avoid inappropriate prescriptions in geriatric outpatients during discharge from emergency care.	1/63*/5,162	Emergency department visits resulting in prescriptions for ≥1 of the 9 targeted inappropriate medications.	**+**	...	**..**.

Gurwitz, 2008[25]	7	Alerts to prevent adverse drug events in long-term care.	2*/37/1,118	...	**..**.	Adverse drug events.	**0**

Hicks, 2008[26]	7	Reminders for management of hypertension in adults in primary care.	14*/.../2,027	Visits with adherence to guideline medication prescribing within one week.	**+**	BP controlled.	**0**

Matheny, 2008[28]	8	Reminders for routine medication laboratory monitoring in primary care.	20*/303/1,922	Ordering appropriate laboratory tests.	**0**	...	**..**.

Reeve, 2008[30]	8	Reminders for use of aspirin in diabetic adults in primary care.	52*/150/258,979	Number of aspirin interventions in diabetic patients.	**+**	...	**..**.

Davis, 2007[32]	9	Alerts for appropriate prescribing for upper respiratory tract infections in paediatric outpatients.	2/44*/12,195	Prescriptions consistent with recommendations.	**+**	...	**..**.

Heidenreich, 2007[33]	7	Reminders to prescribe β-blockers for inpatients and outpatients with reduced LVEF.	3/50/1,546*	Patients with prescriptions for any β-blocker over nine months.	**+**	Survival free of heart failure hospitalization at one year.	**0**

Martens, 2007[34,46]	9	Reminders for prescribing of antibiotics and drugs for asthma, COPD, and dyslipidaemia.	23*/53/3,496	Sum scores for appropriate prescribing of antibiotics, statins, cholesterol-lowering drugs or drugs for asthma or COPD.	**0**	...	**..**.

Peterson, 2007[35]	4	Dosing advice for high-risk drugs in geriatric patients in a tertiary care academic health centre.	1/778/2,981*	Ratio of overall prescribed to recommended doses.	**+**	...	**..**.

Raebel, 2007a[37]	8	Alerts to review potentially inappropriate prescriptions in ambulatory geriatric patients.	21/.../59,680*	Dispensings of targeted potentially inappropriate medications.	**+**	...	**..**.

Raebel, 2007b[36]	7	Alerts to avoid teratogenic drugs in pregnant ambulatory patients.	.../.../11,100*	Dispensed category D or X drugs.	**+**	...	**..**.

Thomson, 2007[38]	8	Presented information for treatment decisions about warfarin or aspirin therapy for patients with atrial fibrillation in primary care.	2/2/109*	Difference in decision conflict scale score immediately post-clinic.	**+**	Admission to hospital; adverse events including transient ischemic attack, bleeding or stroke followed by general practitioner consultation or admission; patient anxiety.	**0**

Verstappen, 2007[39]	6	Management of methotrexate for early rheumatoid arthritis in adult outpatients.	6/.../299*	...	**..**.	Patients in remission for ≥3 months in first two years.	**+**

Feldstein, 2006a[22,41]	10	Alerts to order laboratory tests when prescribing new medications in primary care.	15*/200/961	Baseline laboratory monitoring completed by day 25.	**+**	...	**..**.

Judge, 2006[42]	8	Alerts to avoid inappropriate prescriptions in long-term care.	1*/27/445	Appropriate prescriber response to alerts.	**0**	...	**..**.

Kattan, 2006[43]	8	Feedback provided for management of drug therapy for severe asthma in paediatric outpatients.	.../435/937*	Time to compliance with recommended therapy step; visits resulting in medication step-up after step-up recommendation.	**+**	Maximum symptom days per two weeks.	**0**

Palen, 2006[47]	9	Reminders for laboratory monitoring based on medication orders in primary care.	16/207*/26,586	Overall compliance with ordering the recommended laboratory monitoring.	**0**	...	**..**.

Paul, 2006[48]	10	Recommendations for empiric antibiotic treatment in hospital inpatients.	15*/.../2,326	Overall rate of appropriate antibiotic treatment.	**+**	Duration of hospital stay or fever; 30-day mortality.	**0**

Derose, 2005[50]	7	Reminders to prescribe ACE-Is, angiotensin receptor blockers and/or statins in outpatients with diabetes or atherosclerosis.	.../1089/8,557*	Patients with prescriptions for at least one of ACE-I, angiotensin receptor blocker, or statin.	**+**	...	**..**.

Heidenreich, 2005[51]	6	Reminders to prescribe ACE-I or alternative for inpatients and outpatients with reduced LVEF.	1/.../600*	Patients with prescriptions for ≥ moderate daily dose of ACE-I or appropriate alternative.	**0**	Mortality; renal function; creatinine; systolic BP; diastolic BP.	**0**

Raebel, 2005[54]	8	Alerts to order laboratory tests when prescribing new medications in primary care.	.../.../400,000*	Laboratory test completed at time prescription is dispensed.	**+**	...	**..**.

Krall, 2004[58]	8	Alerts to prescribe of low dose aspirin therapy in primary care.	.../100*/10,972	Provider response to alerts (prescribe aspirin or document contraindication).	**+**	...	**..**.

Ansari, 2003[61]	7	Alerts to prescribe β-blockers for patients with heart failure in primary care.	1/74*/169	Initiated or uptitrated and maintained on β-blockers; patients at target β-blocker dose.	**0**	Proportion of patients hospitalised or with emergency department visits; deaths.	**0**

Filippi, 2003[62]	7	Reminders to prescribe acetylsalicylic acid or other antiplatelet agents to diabetic primary care patients.	.../300*/15,343	Antiplatelet drug prescription.	**+**	...	**..**.

Tamblyn, 2003[65]	7	Alerts to avoid inappropriate prescriptions in geriatric outpatients.	.../107*/12,560	Proportion of new inappropriate prescriptions; discontinuation of pre-existing inappropriate prescriptions.	**+**	...	**..**.

Weir, 2003[67]	8	Recommendations for appropriate prescribing of anti-platelet and/or anti-coagulant drugs following stroke or transient ischemic attack for in- and out-patients	16*/.../1,952	Number of optimal treatments provided and rank of therapy prescribed.	**0**	Reduction in ischemic and haemorrhagic vascular events.	**0**

Zanetti, 2003[68]	8	Alert to redose prophylactic antibiotics during prolonged cardiac surgery.	1/.../447*	Intraoperative redose of antibiotics.	**+**	Surgical-site infection.	**0**

Christakis, 2001[73]	5	Recommendations for appropriate prescribing of antibiotics for otitis media in paediatric outpatients.	1/38*/488	Antibiotics prescribed for <10 days.	**+**	...	**..**.

Rossi, 1997[81]	9	Reminders to modify drug therapy in hypertensive outpatients receiving calcium channel blockers as initial therapy.	1/71/719*	Prescription changes from a calcium channel blocker to another antihypertensive agent.	**+**	...	**..**.

Rotman, 1996[83]	7	Alerts to prescribe lowest cost drug alternative for adult outpatients.	1/37*/...	Rate of clinically relevant drug interactions.	**0**	...	**..**.

McDonald, 1980[87]	5	Detection and management of medication-related problems in outpatients.	1/31*/...	Response rate for reminders over five weeks.	**+**	...	**..**.

Coe, 1977[88]	4	Recommendations for medication management of hypertension in patients attending hypertension clinics.	2/.../116*	...	**..**.	Adequate BP control achieved.	**0**

McDonald, 1976[89]	2	Recommendations for laboratory tests to detect potential medication-related events in adults attending a diabetes clinic.	1/.../226*	Ordered required tests to monitor drug effects; appropriate response to abnormal measures.	**+**	...	**..**.

**Studies of multi-faceted interventions**							

Bertoni, 2009[16,21]	9	Recommendations for screening and treatment of dyslipidaemia in primary care.	59*/.../3,821	Patients with appropriate lipid management at follow-up.	**+**	...	**..**.

Gilutz, 2009[19]	7	Reminders for monitoring and treatment of dyslipidaemia in primary care patients with known coronary artery disease.	112*/600/7,448	Appropriate initiation, up-titration, or continuation of statin therapy; adequate lipoprotein monitoring.	**+**	Change in low-density lipoprotein level.	**+**

Javitt, 2008[27]	6	Patient-specific recommendations for detecting and correcting medical errors in a health maintenance organization setting.	1/1378/49,988*	Resolution for problems identified by care considerations by adding or stopping a drug.	**+**	...	**..**.

Quinn, 2008[29]	6	Recommendations for management of type 2 diabetes in primary care using remote glucose monitoring.	3/26/30*	Medications intensified; Medication errors identified.	**+**	Change in HbA1c levels.	**+**

Van Wyk, 2008[31]	10	On-demand and automatic alerts to screen and treat dyslipidaemia in primary care.	38*/80/92,054	Patients requiring treatment were treated.	**Auto, +** **On-demand, 0**	...	**..**.

Feldstein, 2006b[40]	8	Reminders for monitoring and treatment of osteoporosis care in high-risk women in primary care who experienced a fracture.	15/159/311*	Received only osteoporosis medication within 6 months of study start.	**+**	...	**..**.

Kuilboer, 2006[44]	10	Recommendations for monitoring and treatment of asthma and COPD in primary care.	32*/40/156,772	Number of prescriptions for respiratory drugs.	**0**	...	**..**.

Lester, 2006[45,59]	8	Recommendations for the management of dyslipidaemia in primary care.	1/14/235*	Change in statin prescriptions at 1 and 12 months.	**+**	Change in low-density lipoprotein levels.	**0**

Cobos, 2005[49]	10	Recommendations for treatment, monitoring and follow-up for patients with dyslipidaemia in primary care.	42*/.../2,221	Assessed AST/ALT measurements or creatine kinase determinations; use of lipid-lowering drugs in patients with CHD or those without CHD and at high-risk or low-risk.	**0**	Successful management of cardiovascular risk.	**0**

Javitt, 2005[52]	6	Recommendations for management of patients whose care deviates from recommended practices in primary care.	.../.../39,462*	Compliance with recommendations to add or discontinue a medication.	**+**	Hospital admissions, inpatient days, and length of hospital stay.	**+**

Plaza, 2005[53]	9	Recommendations for cost-effective management of asthma in primary care.	.../20*/198	Prescriptions of oral glucocorticoids	**+**	St. George Respiratory Questionnaire total score.	**+**

Sequist, 2005[55]	6	Reminders for management of diabetes and coronary artery disease in primary care.	20*/194/6,243	Receipt of recommended drugs in diabetes (statins, ACE-Is in hypertension) or coronary artery disease (including aspirin, β-blocker or statin use).	**0**	...	**..**.

Tierney, 2005[56]	9	Recommendations for the management of asthma and chronic obstructive pulmonary disease in adults in primary care.	4/266*/706	Suggestions adhered to for starting, modifying or stopping bronchodilators; medication compliance; patient satisfaction with physicians and pharmacists.	**0**	SF-36 subscale scores; McMaster Asthma Quality of Life and Chronic Respiratory Disease Questionnaires overall scores; emergency department visits; hospitalizations.	**0**

Wolfenden, 2005[57]	7	Reminders to provide smoking cessation interventions to patients attending non-cardiac pre-operative clinic.	1/18/210*	Preoperative nicotine replacement therapy offered or prescribed.	**+**	...	**..**.

Murray, 2004[60]	5	Recommendations for treatment of hypertension in primary care.	4/...*/712	Compliance with all antihypertensive drug suggestions; patient satisfaction.	**0**	Overall composite quality of life score.	**0**

Tierney, 2003[66]	10	Recommendations for management of heart disease in primary care.	4*/115/706	Adherence with care suggestions to start low-dose aspirin or antihyperlipidemic drugs, or start or increase an ACE-Is, β-blockers, diuretics, long-acting nitrates, or calcium blockers.	**0**	Quality of life (score SF-36) at 12 months; quality of life (Chronic heart disease questionnaire overall health status).	**0**

Eccles, 2002[64,69]	10	Recommendations for management of asthma or angina in adults in primary care.	62*/.../4,506	Prescription of β-blockers for angina patients.	**0** **(angina group)**	Overall and disease-specific quality of life for angina patients.	**0** **(angina group)**

Flottorp, 2002[63,70]	9	Recommendations for management of urinary tract infections in women or sore throat in primary care.	142*/.../...	Use of antibiotics for sore throat and urinary tract infections.	**+**	...	**..**.

Lesourd, 2002[71]	5	Recommendations for hormonal ovarian stimulation for infertile women in a teaching hospital.	3/4/164*	...	**..**.	Pregnancy rate.	**0**

Selker, 2002[72]	8	Recommendations for thrombolytic and other reperfusion therapy in acute myocardial infarction.	28/.../1,596*	Patients who had ST-segment elevation detected but did not have acute myocardial infarction, including those who received thrombolytic therapy and those who had contraindications; thrombolytic therapy prescribed within one hour of acute myocardial infarction.	**0**	Death, stroke, or thrombolysis-related bleeding events that required transfusion within 30 days follow-up.	**0**

Dexter, 2001[74]	10	Reminders for preventive therapies in hospital inpatients.	...*/202/3,416	Hospitalizations with an order for prophylactic heparin or aspirin at discharge (all patients and only eligible patients).	**+**	...	**..**.

McCowan, 2001[75]	8	Recommendations and reminders for management of asthma in primary care.	...*/46/477	Received prescription for acute asthma exacerbations.	**+**	Acute exacerbation of asthma.	**+**

Demakis, 2000[76]	7	Reminders for screening, monitoring, and counselling in accordance with predefined standards of care in ambulatory care.	12*/275/12,989	Adherence to recommendations for international normalised ratio monitoring in warfarin users, anticoagulation in atrial fibrillation, β-blocker following myocardial infarction or change in non-steroidal anti-inflammatory drug therapy following gastrointestinal bleed.	**0**	...	**..**.

Hetlevik, 1999[77-79]	8	Recommendations for diagnosis and management of hypertension, diabetes mellitus, and dyslipidaemia in primary care.	56*/56/3,273	...	**..**.	For hypertension and diabetic patients, change at 21 months in systolic and diastolic BP, serum cholesterol, BMI, proportion of smokers, CHD risk score (women or men), proportion of patients with cardiovascular inheritance, or HbA1c level (diabetic patients only).	**0**

Overhage, 1997[80]	8	Recommendations for corollary orders to prevent errors of omission for tests and treatments in general medicine inpatients.	1*/92/2,181	Pharmacist interventions with physicians for significant errors.	**+**	Days in hospital; maximum serum creatinine level during hospital stay.	**0**

Overhage, 1996[82]	10	Reminders to comply with 22 US Preventive Services Task Force preventive care measures for hospital inpatients.	1*/78/1,622	Compliance with preventive care guidelines for use of aspirin, oestrogen or calcium, ACE-Is, heparin prophylaxis, and β-blockers.	**0**	...	**..**.

Tierney, 1993[84]	10	Alerts for drug allergies and drug-drug interactions, and options for cost-effective testing in inpatients.	6*/276/5,219	...	**..**.	Length of hospital stay and resource use after discharge (outpatient visits and readmissions).	**0**

Mazzuca, 1990[85]	7	Reminders for the management of type 2 diabetes mellitus in outpatients.	4*/114/279	Initiation of oral hypoglycaemic therapy.	**0**	...	**..**.

McAlister, 1986[86]	7	Recommendations for management of hypertension in primary care.	50/50*/2,231	Patients receiving treatment for hypertension.	**0**	Diastolic BP ≤90 mmHg on last visit; days with diastolic BP ≤90 mmHg; change in diastolic BP from baseline.	**0**

Abbreviations: ACE-I = angiotensin converting enzyme inhibitor; BP = blood pressure; CCDSS = computerized clinical decision support system; CHD = coronary heart disease; COPD = chronic obstructive pulmonary disease; LVEF = left ventricular ejection fraction.

*Unit of allocation.

^a^Outcomes are evaluated for effect based on the following hierarchy, with an effect defined as ≥50% of relevant outcomes showing a statistically significant difference (2*p *< 0.05):

1. If a single primary outcome is reported, *in which all components are applicable*, this is the only outcome evaluated.

2. If >1 primary outcome is reported, the ≥50% rule applies and only the primary outcomes are evaluated.

3. If no primary outcomes are reported (or only some of the primary outcome components are relevant) but overall analyses are provided, the overall analyses are evaluated as primary outcomes. Subgroup analyses are not considered.

4. If no primary outcomes or overall analyses are reported, or only some components of the primary outcome are relevant for the application, any reported prespecified outcomes are evaluated.

5. If no clearly prespecified outcomes are reported, any available outcomes are considered.

6. If statistical comparisons are not reported and data are insufficient to conduct analyses, 'effect' is designated as not evaluated (...).

### Study characteristics

Thirty-six trials (55%) [17,18,20,22-26,28,30,32-39,41-43,46-48,50,51,54,58,61,62,65,67,68,73,81,83,87-89] described systems classified as drug therapy-only with the remaining 29 (45%) [16,19,21,27,29,31,40,44,45,49,52,53,55-57,59,60,63,64,66,69-72,74-80,82,84-86] describing multi-faceted CCDSSs. Forty-one of 65 included studies (63%) [16-59,63,68,70] were published since the previous version of this review. Eleven trials (17%) were published prior to 2000 [77-89], 16 (25%) trials [58,60-76] between 2000 and 2004 and 38 (58%) trials [16-57,59] after 2004. Most studies (n = 41, 63%) [16-21,23,24,26,28,31-33,38,40,42-44,47,48,55-57,60,61,63,66-68,70,72-80,82-88] reported public funding; nine (14%) [29,34,35,45,46,49,50,52,53,59,71] reported private funding; six (9%) [22,36,37,41,54,64,65,69] reported public and private funding, and 9 (14%) [25,27,30,39,51,58,62,81,89] did not disclose a funding source (see Additional file 3, Table S3). We were able to determine whether improvement occurred with a CCDSS for process of care outcomes in 59 studies [16-24,26-38,40-70,72-76,80-83,85-87,89]; 29 studies reported patient outcomes [19,25,26,29,33,38,39,43,45,48,49,51-53,56,59-61,64,66-69,71,72,75,77-80,84,86,88], and both patient and process outcomes were extracted from 23 (of 29, 79%) reports [19,26,29,33,38,43,45,48,49,51-53,56,59-61,64,66-69,72,75,80,86] (Table 1 and see Additional file 4, Table S4). Twenty [32,33,38,39,43,48,51-53,56,60,61,66-69,71-73,75,80,84] of 29 (69%) studies reported a patient important outcome rather than an intermediate or surrogate outcome [90].

### Study quality

Included trials had a median methodological quality score of 8 (interquartile range [IQR], 2) of a total possible score of 10. Quality assessments for each trial are presented in Additional file 1, Table S1. Most included studies were cluster randomized (n = 44/65, 68%) [16-26,28,30-32,34,41,42,44,46-49,53,55,56,58,60-67,69,70,73-87], measured an objective outcome or blinded outcome assessments appropriately (n = 64/65, 98%) [16-56,58-89], had 80% or greater follow-up of subjects (n = 56, 86%) [16-38,40-50,52-54,56-59,61-72,74,76-79,81-87] and 41 (63%) [16,18,20-23,30-34,36-51,53,54,56-59,63,64,66,68-70,72,74,75,77-82,84] reported adequate allocation concealment. There was no change in quality score over time (R^2 ^= 0.01, *p *= 0.53).

### CCDSS and study characteristics

Additional file 2, Table S2 describes CCDSS users and Additional file 3, Table S3 describes study settings. A sum of 8,932 providers (median, 80; IQR, 193) used a CCDSS to assist with drug management for a total studied population of 1,246,686 patients (median, 2027; IQR 6960). Most CCDSSs were used by fully-trained physicians (61/65, 94%) [16-29,31-35,38-56,58-89] and some by post-graduate medical trainees (19/65, 29%) [20,23,32,35,55,56,60,66,73,74,76,80-82,84,85,87-89]. After physicians, nurses in advanced practice roles (16/25 studies, 25%) [16,18,20-22,25-27,33,35,41,42,57,58,68,73,81,87], physician assistants 8/65, 12%) [16,18,20,21,25,42,58,63,70,77-79], and pharmacists (8/65, 12%) [30,35-37,54,56,60,66] were the most common provider types interacting with CCDSSs. Many systems reported use by more than one type of provider. CCDSSs were studied in the United States (n = 44, 68%) [16,18,20-23,26-30,32,33,35-37,40-43,45,47,50-52,54-56,58-61,66,68,72-74,76,80-85,87-89], European Union or European Economic Area countries (n = 13, 20%) [31,34,38,39,44,46,49,53,62-64,69-71,75,77-79], and Canada (n = 3, 5%) [17,24,65,86], with the remaining five studies (8%) [19,25,48,57,67] occurring in multiple or other countries.

Outpatient settings were studied more often (n = 55, 85%) [16,18-23,26-41,43-47,49-67,69-71,73,75-79,81,83,85-89] than other settings of care. Studies were conducted in both academic settings (n = 34, 52%) [18,23,25,26,28,33-35,38,39,42,46,48,51,55-57,60,61,66,68,71-74,76,80-85,87-89] and outside academic centres (n = 31, 48%) [16,17,19-22,24,27,29-32,36,37,40,41,43-45,47,49,50,52-54,58,59,62-65,67,69,70,75,77-79,86].

As presented in Additional file 2, Table S2, the majority of CCDSS systems in our sample were integrated with an EMR (n = 38/61, 62%) [17,18,20,23-26,28,31,32,34,40,42,44-47,49,55,56,58-66,68-70,73,74,77-85,87,89], delivered feedback via a computer display (n = 44/62, 71%) [17,18,20,23-26,28,30-36,38-40,42,44,46-49,51,55-58,60-66,68-71,73-76,80,82-84] at the time of care (n = 53/64, 83%) [16-21,23-26,28,30-35,38,40,42,44,46-51,53,55-58,60-70,72-85,87-89]. A minority of authors reported testing a CCDSS with a graphical user interface (n = 22/25, 88%) [16-18,20,21,23-25,28,30,31,34,38,40,45,46,55,56,58-60,63,65,70,73,75,83], pilot-testing the system before the trial (n = 25/45, 56%) [17-19,24,28,29,31-33,35,38,39,43-45,47,48,55,59,63,66,70-72,74,75,77-79,84], or training users on system use (n = 29/52, 56%) [16,19,21,26,28,29,31,32,34,35,38,43-47,53,56-60,62,64-66,69,75-79,83-85]. Data required by the CCDSS to produce recommendations were most commonly entered via EMR link (n = 32/61, 52%) [17,18,20,24,26,28,31,32,34,40,44-47,49,51,55,56,58-60,62-66,68-70,74,80-82,84,85,87,89], followed by provider entry (n = 23/61, 38%) [16,21,23,25,30,31,34,35,38,39,46,53,56,63,64,66,67,69-71,73,75,76,80,83,86,88], study staff (n = 10/61, 16%) [19,39,42,43,48,61,63,70,74,86,89], and existing staff (n = 8/61, 13%) [36,37,68,72-74,88,89], although multiple modes of entry were reported in some studies. Nineteen (29%) [17,18,20,23-25,31,32,34,35,42,45-47,56,58-60,80,82-84] studies reported using systems that were integrated with CPOE.

### Clinical characteristics

CCDSSs were grouped into one of three categories representing the primary pharmacotherapeutic purpose of the system. Systems designed to optimize drug therapy were tested in 47 (72%) trials [16,19,26,27,29-34,38-40,43-45,48-53,55-58,60-62,66-76,78,81,82,85-88]; systems to prevent adverse drug events accounted for 16 (25%) trials [17,20,23,25,28,35-37,41,42,47,54,65,80,84,89];while the remaining two (3%) trials [18,83] focused on drug cost management. Patient populations were identified (for each system) and consisted of seven (11%) systems for geriatric patients [17,23,25,35,37,42,65], three (5%) systems for paediatrics [32,43,73], four (6%) systems for women's health [36,40,70,71], and 51 (78%) for adults or unspecified general populations. We attempted to identify the main disease state targeted by each system. Sixteen systems (25%) [17,23,25,27,34,35,37,42,52,58,65,74,76,82-84] were employed for multiple conditions. Each of the following disease groupings included three or more systems: cardiovascular disease [26,33,51,60,61,66,69,72,81,86,88] (n = 11, 17%), diabetes mellitus [29,30,50,55,62,78,85] (n = 7, 11%), respiratory disease [43,44,53,56,75] (n = 5, 8%), dyslipidaemia [16,19,31,45,49] (n = 5, 8%) and infectious diseases [32,48,68,70,73] (n = 5, 8%). Nine of the remaining 16 systems [20,28,36,41,47,54,80,87,89] were designed to prevent or detect drug related problems via laboratory monitoring.

### CCDSS effectiveness

Thirty-seven trials [16,18,19,21-23,26,27,29-33,35-38,40,41,43,45,48,50,52-54,57-59,62,63,65,68,70,73-75,80,81,87,89] of 59 (63%) showed improvement in process of care outcomes due to CCDSS use. No significant difference was found between Rx-only (23/33) [18,22,23,26,30,32,33,35-38,41,43,48,50,54,58,62,65,68,73,81,87,89] and multi-faceted (14/26) [16,19,21,27,29,31,40,45,52,53,57,59,63,70,74,75,80] CCDSSs for process of care improvement.

Six trials [19,29,39,52,53,75] (9% of all trials, 21% of trials measuring a patient outcome) demonstrated improved patient outcomes with CCDSS use compared to usual care without a CCDSS (see Table 1). Four [39,52,53,75] of the six trials demonstrating improved patient outcomes measured patient-important outcomes. No significant difference in improvement was found between drug-only (1/12) [39] and multi-faceted (5/17) [19,29,52,53,75] CCDSSs.

Results did not significantly vary, for either process of care or patient outcomes, by the type of outcome (primary, pre-specified, or other) selected to determine improvement.

All studies demonstrating improved patient outcomes also showed improvement in measured process of care outcomes.

The proportion of successful trials was not significantly different between cluster trials where units of allocation were mismatched with units of analyses (7/15 for process and 1/5 for patient outcomes) compared with non-cluster trials or cluster trials with an appropriately adjusted analysis (29/43 for process (*p = *0.22) and 5/24 for patient outcomes (*p *= 1)).

### Predictors of success

This analysis was limited by incomplete data in many studies and by limited power for multivariate analysis. In univariate analysis, CCDSSs not integrated with an EMR were more likely to improve process of care outcomes, 16/20 (80%) non-integrated systems showed improvement versus 18/35 (51%) improved with EMR linkage (*p = *0.03). The same trend was seen with integration of EMR and CCDSS for patient outcomes (6/15 (40%) improved outcomes without EMR link versus (0/13 (0%) with EMR link, *p *= 0.017). This association between EMR integration and CCDSS failure was not statistically significant via multivariate regression.

Improvement in process of care or patient outcomes was not affected by integration with CPOE, timing or method of decision support delivery, or method of data entry. Improvement in process or patient outcomes did not vary by country, provider type, and outpatient versus other settings of care. Systems trialed outside of academic settings were more likely to improve patient outcomes (5/12 (42%) outside academic settings versus 1/17 (6%) in academic settings, *p *= 0.03). This finding was not replicated in multivariate analyses. Investigators who developed the system under study were not significantly more likely to see improvement with a CCDSS than investigators studying systems developed by unrelated parties (*p *= 0.56 for process and *p *= 1 for patient outcomes). Patient important outcomes were as likely as surrogate outcomes to show improvement with a CCDSS. *Post hoc*, none of primary disease state, primary patient population, or pharmacotherapeutic purpose predicted success. We found no association between the presence of a sample size calculation and success or between number of trial participants and success. The proportion of studies added in this update demonstrating benefit with CCDSS for process of care and patient outcomes increased compared with studies included in the previous review version, although this trend was not statistically significant.

### Costs and practical process related outcomes

#### Harms

Potential or actual harm resulting from CCDSS use was explicitly discussed in four (6%) [16,21,36,68,89] included studies (see Additional file 5, Table S5). Two studies reported quantitative data regarding harms. Raebel *et al. *[36] reported a trial stopped early due to a high rate (40%) of clinically inappropriate reminders generated by the CCDSS. Zanetti *et al. *[68] reported one inappropriate redose of intra-operative prophylactic antibiotic for every 137 appropriate redose reminders.

#### Costs

Some information on financial or economic costs associated with CCDSS was reported for 15 (23%) [17,19,24,27,43,48,49,52,53,56,57,60,66,80,83,84] trials (see Additional file 5, Table S5). A formal cost-effectiveness analysis for a patient outcome was performed in only one case [53]. Twelve trials compared direct healthcare costs between CCDSSs and control groups with mixed results: significantly decreased costs were observed in six trials [27,43,48,49,52,84], no significant change in five trials [53,60,66,80,83], and significantly increased costs in one trial [56].

#### User satisfaction

Fifteen authors reported on user satisfaction with the CCDSS studied (see Additional file 5, Table S5) [18,19,29,30,33,39,55,57,63,64,67,69,70,75,77-79,83,84]. All attempts to measure user satisfaction were conducted via surveys and the properties of the measure used were only discussed in a single trial [55]. Survey response rates ≥50% were found in eight studies. Of these eight, six reported [18,29,33,55,57,84] that ≥70% of respondents thought the CCDSS improved care, was useful, or should be continued in use. Satisfaction data from the other two trials [77-79,83] suggested users could not or would not use the CCDSS due to technical or user interface problems. Available data on user satisfaction were too sparse to determine if satisfaction impacted study results.

## Discussion

We reviewed 65 RCTs of CCDSSs for drug therapy management reported over a 34-year span. Most trials measured process of care outcomes and results supported the use of CCDSSs to improve these outcomes in a majority of cases (improvement was based on at least 50% of the relevant study outcomes being statistically significantly positive). However, while nearly one-half of (29 studies) included studies measured a patient outcome, only a small proportion demonstrated any direct benefit to patients. While improvement in process outcomes could lead to benefits for patients, no consistent link was observed here. In the absence of data needed for an economic analysis, improved process of care measures alone are not sufficient to recommend adoption of these systems. The success rates we found for processs of care (64%) and clinical outcome measures (21%) are similar to those in our previous review [2] and also a recent umbrella review of systematic reviews of computerized decision support (57% and 30% respectively) by Jaspers *et al. *[91].

Several possible predictors of CCDSS success were examined. In most cases, these *a priori *factors did not explain success or failure across included studies. Our previous review [2] concluded that successful trials of CCDSS were more likely to have been conducted by the developers of the system under study. In our current review, no such association was noted. Previously, a significant trend towards increased study quality over time was noted, but not replicated in this update, and we attribute this to a more restrictive inclusion criterion (randomized controlled trials). Counter to our expectations, we found that integration of CCDSSs with EMRs and use in an academic setting was associated with CCDSS failure. This trend was not statistically significant when tested using multi-variate techniques and so we are unable to determine whether this finding represents a true association or is better explained by the lack of power in our multi-variate analysis. We report these findings as hypothesis generating only and suggest they be examined in future.

Compared with the review of Kawamoto *et al. *[92], we did not find that automatic provision of advice as part of the existing clinical workflow predicted CCDSS success. Because both the current analysis and that of Kawamoto were underpowered to detect such associations, we have refrained from drawing any conclusions in this regard.

Prospective data on the possible harms of CCDSSs are needed to facilitate informed adoption decisions. Only two trials quantitatively reported on harm from CCDSSs [36,68] with one trial ending early due to increased risk of harm with the CCDSS. We suggest this absence of evidence of harm should not be taken as proof that CCDSSs are safe to employ for drug management in patient care.

### Strengths and limitations of review

The results of our review should be interpreted with consideration of methodological strengths and limitations, including steps taken to mitigate the risk of bias. We based our review on the strongest studies available, RCTs. Reviews are necessarily retrospective and we employed multiple methods to limit the introduction of bias, including: duplicate study eligibility assessment, duplicate data abstraction, solicitation of study author feedback on abstracted data, and objective selection of outcomes used to determine improvement. We cannot exclude the possibility that a different method of selecting outcomes from each study to measure improvement could lead to different results, although sensitivity analyses did not suggest this to be the case. Several pre-specified analyses of possible predictors of system success were conducted. Several analyses demonstrated statistically significant results using univariate techniques that were not substantiated using a multi-variate model. Therefore, the few associations we reported between possible predictors of success and improved outcomes with CCDSS should be interpreted with caution.

We have relied upon vote counting as our method of obtaining an estimate of how often CCDSS for drug therapy management improve process or patient outcomes. Significant limitations to this approach as described by Hedges [93] include a tendency to inflate type II error and inadequate incorporation of the effect of unequal study sizes in overall results. The heterogeneity between studies included in our review precluded the use of more robust combination techniques. Formal assessment for publication bias using funnel plots was not possible with the vote-counting technique.

The effectiveness of any CCDSS will be determined in part by the efficacy of the underlying action suggested by the system. Where no benefit was detected with a particular CCDSS, we cannot exclude the possibility that the negative finding is due to a lack of efficacy of the intervention suggested by the system. Measurement of the concordance between decision advice given and followed would be a useful measure to address this issue. These outcomes were included in our analyses of process of care outcomes. It does not necessarily follow, however, that an effective CCDSS that recommends the appropriate prescription of an efficacious intervention will necessarily improve patient care. A multitude of intervening factors (*e.g. *patient non- or over-adherence or new errors introduced by CCDSS) may mitigate (or exaggerate) estimates of CCDSS effectiveness.

Finally, the systems reviewed constitute a heterogeneous group with differing functionality and clinical intent. While we have attempted to usefully divide the systems for the reader, we acknowledge other divisions were possible.

### Implications for practice and research

Because CCDSSs have not been shown to reliably and positively impact patients, and in the absence of useful data on potential harms, costs, and clinician impacts, we cannot recommend the general adoption of CCDSSs for drug therapy management. It is possible that these systems are still evolving and success will improve with time. Clearly further innovation is needed if these systems are to be dependably useful in clinical practice. Rigorous trials of these innovations will be necessary, and we suggest that future research explicitly address patient outcomes, including potential harms, and costs and adverse clinician impacts of CCDSSs. Given the availability of effective non-computerized approaches for promoting safe and effective medication use [5,94], future studies may wish to incorporate these interventions as active comparators to CCDSSs.

## Conclusions

CCDSSs inconsistently improved process of care measures and seldom improved patient outcomes. Lack of clear patient benefit and lack of data on harms and costs preclude a recommendation to adopt CCDSSs for drug therapy management.

## Competing interests

RBH, NLW, JAM, LWK, TN, BJH, AH, MT received support through the Canadian Institutes of Health Research Synthesis Grant: Knowledge Translation KRS 91791 for the submitted work. RBH is acquainted with several CCDSS developers and researchers, including authors of papers included in this review.

## Authors' contributions

RBH was responsible for study conception and design; acquisition, analysis, and interpretation of data; drafting and critical revision of the manuscript; obtaining funding; study supervision. He is the guarantor. BJH acquired, analyzed and interpreted data; drafted the manuscript; and conducted statistical analysis. AH analyzed and interpreted data as well as critically revised the manuscript. MT critically revised the manuscript. JAM acquired, analyzed, and interpreted data; drafted the manuscript; and provided statistical analysis. LWK and TN acquired data and drafted the manuscript. NLW acquired, analyzed, and interpreted data; provided administrative, technical, or material support; and provided study supervision. All authors read and approved the final manuscript.

## Supplementary Material

Additional file 1 **Study methods scores for trials of drug prescribing**. Methods scores for the included studies. Click here for file

Additional file 2 **CCDSS characteristics for trials of drug prescribing**. CCDSS characteristics of the included studies. Click here for file

Additional file 3 **Study characteristics for trials of drug prescribing**. Study characteristics of the included studies. Click here for file

Additional file 4 **Results for CCDSS trials of drug prescribing**. Details results of the included studies. Click here for file

Additional file 5 **Costs and CCDSS process-related outcomes for drug prescribing**. Cost and CCDSS process-related outcomes for the included studies. Click here for file
