# Supplementary material for: Computerized clinical decision support systems for drug prescribing and management: A decision-maker-researcher partnership systematic review
Source: Implement Sci. 2011 Aug 3;6:89. doi: 10.1186/1748-5908-6-89 (PMC3179735; doi:10.1186/1748-5908-6-89)
Supplement: Additional file 1 — Study methods scores for trials of drug prescribing. Methods scores for the included studies. [file 1748-5908-6-89-S1.DOCX]

**Additional file 1, Table S1. Study methods scores for trials of drug prescribing^a^**

| **Study** | **Allocation concealed^b^** | **Cluster randomization** | **Adjustment for baseline differences** | **Objective outcome** | **Adequate follow-up** | **Total score** |
| --- | --- | --- | --- | --- | --- | --- |
| McDonald, 1976[89] | 0 | 0 | 0 | 2 | 0 | 2 |
| Coe, 1977[88] | 0 | 0 | 2 | 2 | 0 | 4 |
| McDonald, 1980[87] | 0 | 1 | 0 | 2 | 2 | 5 |
| McAlister, 1986[86] | 1 | 1 | 2 | 2 | 1 | 7 |
| Mazzuca, 1990[85] | 0 | 2 | 2 | 2 | 1 | 7 |
| Tierney, 1993[84] | 2 | 2 | 2 | 2 | 2 | 10 |
| Overhage, 1996[82] | 2 | 2 | 2 | 2 | 2 | 10 |
| Rotman, 1996[83] | 0 | 1 | 2 | 2 | 2 | 7 |
| Overhage, 1997[80] | 2 | 2 | 2 | 2 | 0 | 8 |
| Rossi, 1997[81] | 2 | 1 | 2 | 2 | 2 | 9 |
| Hetlevik, 1999[77-79] | 2 | 2 | 1 | 2 | 1 | 8 |
| Demakis, 2000[76] | 0 | 2 | 1 | 2 | 2 | 7 |
| Christakis, 2001[73] | 0 | 1 | 2 | 2 | 0 | 5 |
| Dexter, 2001[74] | 2 | 2 | 2 | 2 | 2 | 10 |
| McCowan, 2001[75] | 2 | 2 | 2 | 2 | 0 | 8 |
| Eccles, 2002[69, 64] | 2 | 2 | 2 | 2 | 2 | 10 |
| Flottorp, 2002[63, 70] | 2 | 2 | 2 | 2 | 1 | 9 |
| Lesourd, 2002[71] | 0 | 0 | 1 | 2 | 2 | 5 |
| Selker, 2002[72] | 2 | 0 | 2 | 2 | 2 | 8 |
| Ansari, 2003[61] | 0 | 1 | 2 | 2 | 2 | 7 |
| Filippi, 2003[62] | 0 | 1 | 2 | 2 | 2 | 7 |
| Tamblyn, 2003[65] | 0 | 1 | 2 | 2 | 2 | 7 |
| Tierney, 2003[66] | 2 | 2 | 2 | 2 | 2 | 10 |
| Weir, 2003[67] | 0 | 2 | 2 | 2 | 2 | 8 |
| Zanetti, 2003[68] | 2 | 0 | 2 | 2 | 2 | 8 |
| Krall, 2004[58] | 2 | 1 | 1 | 2 | 2 | 8 |
| Murray, 2004[60] | 0 | 1 | 2 | 2 | 0 | 5 |
| Cobos, 2005[49] | 2 | 2 | 2 | 2 | 2 | 10 |
| Derose, 2005[50] | 2 | 0 | 1 | 2 | 2 | 7 |
| Heidenreich, 2005[51] | 2 | 0 | 2 | 2 | 0 | 6 |
| Javitt, 2005[52] | 1 | 0 | 2 | 2 | 1 | 6 |
| Plaza, 2005[53] | 2 | 1 | 2 | 2 | 2 | 9 |
| Raebel, 2005[54] | 2 | 0 | 2 | 2 | 2 | 8 |
| Sequist, 2005[55] | 0 | 2 | 2 | 2 | 0 | 6 |
| Tierney, 2005[56] | 2 | 1 | 2 | 2 | 2 | 9 |
| Wolfenden, 2005[57] | 2 | 0 | 2 | 1 | 2 | 7 |
| Feldstein, 2006a [22, 41] | 2 | 2 | 2 | 2 | 2 | 10 |
| Feldstein, 2006b[40] | 2 | 0 | 2 | 2 | 2 | 8 |
| Judge, 2006[42] | 2 | 2 | 0 | 2 | 2 | 8 |
| Kattan, 2006[43] | 2 | 0 | 2 | 2 | 2 | 8 |
| Kuilboer, 2006[44] | 2 | 2 | 2 | 2 | 2 | 10 |
| Lester, 2006[45, 59] | 2 | 0 | 2 | 2 | 2 | 8 |
| Palen, 2006[47] | 2 | 1 | 2 | 2 | 2 | 9 |
| Paul, 2006[48] | 2 | 2 | 2 | 2 | 2 | 10 |
| Davis, 2007[32] | 2 | 1 | 2 | 2 | 2 | 9 |
| Heidenreich, 2007[33] | 2 | 0 | 2 | 2 | 1 | 7 |
| Martens, 2007[34, 46] | 2 | 2 | 2 | 2 | 1 | 9 |
| Peterson, 2007[35] | 0 | 0 | 0 | 2 | 2 | 4 |
| Raebel, 2007a[37] | 2 | 0 | 2 | 2 | 2 | 8 |
| Raebel, 2007b[36] | 2 | 0 | 1 | 2 | 2 | 7 |
| Thomson, 2007[38] | 2 | 0 | 2 | 2 | 2 | 8 |
| Verstappen, 2007[39] | 2 | 0 | 2 | 2 | 0 | 6 |
| Gurwitz, 2008[25] | 1 | 2 | 0 | 2 | 2 | 7 |
| Hicks, 2008[26] | 0 | 2 | 1 | 2 | 2 | 7 |
| Javitt, 2008[27] | 1 | 0 | 2 | 2 | 1 | 6 |
| Matheny, 2008[28] | 0 | 2 | 2 | 2 | 2 | 8 |
| Quinn, 2008[29] | 0 | 0 | 2 | 2 | 2 | 6 |
| Reeve, 2008[30] | 2 | 2 | 0 | 2 | 2 | 8 |
| Van Wyk, 2008[31] | 2 | 2 | 2 | 2 | 2 | 10 |
| Bertoni, 2009[16, 21] | 2 | 2 | 2 | 2 | 1 | 9 |
| Field, 2009[17, 24] | 0 | 2 | 1 | 2 | 2 | 7 |
| Fortuna, 2009[18] | 2 | 2 | 2 | 2 | 2 | 10 |
| Gilutz, 2009[19] | 0 | 2 | 1 | 2 | 2 | 7 |
| Lo, 2009[20] | 2 | 2 | 2 | 2 | 2 | 10 |
| Terrell, 2009[23] | 2 | 1 | 2 | 2 | 2 | 9 |

^a^ Based on five individual items (score 2 = yes, 1 = partly, and 0 = no) and a summed total score (range 0 to 10). Because this review update included only randomized, controlled trials, the total score differs from that reported in the previous version of this review[2]: The item evaluating study type (randomized, quasi-randomized, or concurrent controls) has been replaced by one that evaluates use of concealed allocation (concealed, unclear, not concealed).

^b^If allocation concealment is not readily apparent from the description provided in the published article, the primary author of the trial confirmed or indicated that allocation was concealed.
