# Supplementary material for: Computerized clinical decision support systems for drug prescribing and management: A decision-maker-researcher partnership systematic review
Source: Implement Sci. 2011 Aug 3;6:89. doi: 10.1186/1748-5908-6-89 (PMC3179735; doi:10.1186/1748-5908-6-89)
Supplement: Additional file 2 — CCDSS characteristics for trials of drug prescribing. CCDSS characteristics of the included studies. [file 1748-5908-6-89-S2.DOCX]

**Additional file 2, Table S2. CCDSS characteristics for trials of drug prescribing**

| **Study** | **Design** | | | **Interface description** | | | | | **Data entry source** | | | | | | **Methods for delivery of recommendations** | | | | | | | **CCDSS users** | | | | | | **Other characteristics** | | | | |
| --- | --- | --- | --- | --- | --- | --- | --- | --- | --- | --- | --- | --- | --- | --- | --- | --- | --- | --- | --- | --- | --- | --- | --- | --- | --- | --- | --- | --- | --- | --- | --- | --- |
|  | **Stand alone** | **Integrated with EMR** | **Integrated with CPOE** | Graphic user interface | User must type | Drop down menus | Drag and drop | Other interface | **Automated through EMR** | **Project staff** | **Existing staff** | **Practitioner/decision-maker** | **Patient** | **Other data entry** | Desktop/Laptop computer | E-Mail | PDA | Pager | Project staff | Existing non-prescribing staff | Other Methods | **Trainees** | **Physicians** | **Advanced Practice Nurses** | **Physician Assistants** | **Pharmacists** | **Other health professionals** | Pilot tested | Users trained | Feedback at time of care | CCDSS suggested diagnoses/ treatments/procedures | Authors as developers |
| McDonald, 1976[89] | **-** | **+** | **-** | ? | ? | ? | ? | ? | **+** | **+** | **+** | **-** | **-** | **-** | - | - | - | - | - | + | - | **+** | **+** | **-** | **-** | **-** | **-** | - | - | + | + | + |
| Coe, 1977[88] | **+** | **-** | **-** | ? | ? | ? | ? | ? | **-** | **-** | **+** | **+** | **-** | **-** | ? | ? | ? | ? | ? | ? | ? | **+** | **+** | **-** | **-** | **-** | **-** | - | - | + | + | + |
| McDonald, 1980[87] | **-** | **+** | **-** | ? | ? | ? | ? | ? | **+** | **-** | **-** | **-** | **-** | **-** | - | - | - | - | + | + | - | **+** | **+** | **+** | **-** | **-** | **-** | - | - | + | + | + |
| McAlister, 1986[86] | **+** | **-** | **-** | ? | ? | ? | ? | ? | **-** | **+** | **-** | **+** | **-** | **-** | - | - | - | - | + | - | - | **-** | **+** | **-** | **-** | **-** | **-** | - | - | - | + | + |
| Mazzuca, 1990[85] | **-** | **+** | **?** | ? | ? | ? | ? | ? | **+** | **-** | **-** | **-** | **-** | **-** | - | - | - | - | - | + | - | **+** | **+** | **-** | **-** | **-** | **-** | - | + | + | + | + |
| Tierney, 1993[84] | **-** | **+** | **+** | ? | + | + | ? | ? | **+** | **-** | **-** | **-** | **-** | **-** | + | - | - | - | - | - | - | **+** | **+** | **-** | **-** | **-** | **-** | + | + | + | - | + |
| Overhage, 1996[82] | **-** | **+** | **+** | ? | ? | ? | ? | ? | **+** | **-** | **-** | **-** | **-** | **-** | + | - | - | - | - | - | + | **+** | **+** | **-** | **-** | **-** | **-** | - | - | + | + | + |
| Rotman, 1996[83] | **-** | **+** | **+** | + | ? | ? | ? | ? | **-** | **-** | **-** | **+** | **-** | **-** | + | - | - | - | - | - | - | **-** | **+** | **-** | **-** | **-** | **-** | ? | + | + | + | - |
| Overhage, 1997[80] | **-** | **+** | **+** | ? | ? | ? | ? | ? | **+** | **-** | **-** | **+** | **-** | **-** | + | - | - | - | - | - | - | **+** | **+** | **-** | **-** | **-** | **-** | - | - | + | + | + |
| Rossi, 1997[81] | **-** | **+** | **-** | ? | ? | ? | ? | ? | **+** | **-** | **-** | **-** | **-** | **-** | - | - | - | - | - | + | - | **+** | **+** | **+** | **-** | **-** | **-** | - | - | + | + | + |
| Hetlevik, 1999[77-79] | **-** | **+** | **-** | ? | ? | ? | ? | ? | **?** | **?** | **?** | **?** | **?** | **?** | ? | ? | ? | ? | ? | ? | ? | **-** | **+** | **-** | **+** | **-** | **-** | + | + | + | + | - |
| Demakis, 2000[76] | **+** | **-** | **-** | - | + | - | - | + | **-** | **-** | **-** | **+** | **-** | **+** | + | - | - | - | - | + | - | **+** | **+** | **-** | **-** | **-** | **-** | - | + | + | + | + |
| Christakis, 2001[73] | **-** | **+** | **?** | + | ? | ? | ? | + | **-** | **-** | **+** | **+** | **-** | **-** | + | - | - | - | - | - | - | **+** | **+** | **+** | **-** | **-** | **-** | - | - | + | + | + |
| Dexter, 2001[74] | **-** | **+** | **-** | ? | ? | ? | ? | ? | **+** | **+** | **+** | **-** | **-** | **-** | + | - | - | - | - | - | - | **+** | **+** | **-** | **-** | **-** | **-** | + | - | + | + | + |
| McCowan, 2001[75] | **+** | **-** | **-** | + | + | + | - | ? | **-** | **-** | **-** | **+** | **-** | **-** | + | - | - | - | - | - | - | **-** | **+** | **-** | **-** | **-** | **-** | + | + | + | + | + |
| Eccles, 2002[64, 69] | **-** | **+** | **-** | ? | ? | ? | ? | ? | **+** | **-** | **-** | **+** | **-** | **-** | + | - | - | - | - | - | - | **-** | **+** | **-** | **-** | **-** | **-** | - | + | + | + | + |
| Flottorp, 2002[63, 70] | **-** | **+** | **?** | + | ? | ? | ? | + | **+** | **+** | **-** | **+** | **-** | **-** | + | - | - | - | - | - | - | **-** | **+** | **-** | **+** | **-** | **-** | + | - | + | + | + |
| Lesourd, 2002[71] | **+** | **-** | **-** | - | + | ? | ? | ? | **-** | **-** | **-** | **+** | **-** | **-** | + | - | - | - | - | - | - | **-** | **+** | **-** | **-** | **-** | **-** | + | - | - | + | + |
| Selker, 2002[72] | **+** | **-** | **-** | ? | ? | ? | ? | ? | **-** | **-** | **+** | **-** | **-** | **-** | - | - | - | - | - | + | - | **-** | **+** | **-** | **-** | **-** | **-** | + | - | + | + | - |
| Ansari, 2003[61] | **-** | **+** | **-** | ? | ? | ? | ? | ? | **-** | **+** | **-** | **-** | **-** | **-** | + | - | - | - | - | - | - | **-** | **+** | **-** | **-** | **-** | **-** | - | - | + | + | + |
| Filippi, 2003[62] | **-** | **+** | **?** | ? | ? | ? | ? | ? | **+** | **-** | **-** | **-** | **-** | **-** | + | - | - | - | - | - | - | **-** | **+** | **-** | **-** | **-** | **-** | ? | + | + | + | ? |
| Tamblyn, 2003[65] | **-** | **+** | **?** | + | ? | ? | ? | ? | **+** | **-** | **-** | **-** | **-** | **+** | + | - | - | - | - | - | - | **-** | **+** | **-** | **-** | **-** | **-** | - | + | + | + | + |
| Tierney, 2003[66] | **-** | **+** | **-** | ? | ? | ? | ? | ? | **+** | **-** | **-** | **+** | **-** | **-** | + | - | - | - | - | - | - | **+** | **+** | **-** | **-** | **+** | **-** | + | + | + | + | + |
| Weir, 2003[67] | **+** | **-** | **-** | ? | ? | ? | ? | ? | **-** | **-** | **-** | **+** | **-** | **-** | - | - | - | - | - | - | + | **-** | **+** | **-** | **-** | **-** | **-** | - | - | + | + | + |
| Zanetti, 2003[68] | **-** | **+** | **?** | ? | ? | ? | ? | + | **+** | **-** | **+** | **-** | **-** | **-** | + | - | - | - | - | + | - | **-** | **+** | **+** | **-** | **-** | **-** | ? | ? | + | + | + |
| Krall, 2004[58] | **-** | **+** | **+** | + | + | - | - | + | **+** | **-** | **-** | **-** | **-** | **-** | + | - | - | - | - | - | - | **-** | **+** | **+** | **+** | **-** | **+** | - | + | + | + | + |
| Murray, 2004[60] | **-** | **+** | **+** | + | - | ? | ? | + | **+** | **-** | **-** | **-** | **-** | **-** | + | - | - | - | - | - | + | **+** | **+** | **-** | **-** | **+** | **-** | ? | + | + | + | + |
| Cobos, 2005[49] | **-** | **+** | **-** | ? | ? | ? | ? | ? | **+** | **-** | **-** | **-** | **-** | **-** | + | - | - | - | - | - | - | **-** | **+** | **-** | **-** | **-** | **-** | ? | ? | + | + | ? |
| Derose, 2005[50] | **?** | **?** | **?** | ? | ? | ? | ? | ? | **?** | **?** | **?** | **?** | **?** | **?** | - | - | - | - | - | - | + | **-** | **+** | **-** | **-** | **-** | **-** | ? | ? | + | + | ? |
| Heidenreich, 2005[51] | **?** | **?** | **?** | ? | - | - | - | + | **+** | **-** | **-** | **-** | **-** | **-** | + | - | - | - | - | - | + | **-** | **+** | **-** | **-** | **-** | **-** | ? | ? | + | + | + |
| Javitt, 2005[52] | **+** | **-** | **-** | ? | ? | ? | ? | ? | **-** | **-** | **-** | **-** | **-** | **+** | - | - | - | - | - | - | + | **-** | **+** | **-** | **-** | **-** | **-** | ? | ? | - | + | + |
| Plaza, 2005[52] | **+** | **-** | **-** | ? | ? | ? | ? | ? | **-** | **-** | **-** | **+** | **-** | **-** | - | - | + | - | - | - | - | **-** | **+** | **-** | **-** | **-** | **-** | ? | + | + | + | ? |
| Raebel, 2005[54] | **?** | **?** | **?** | ? | ? | ? | ? | ? | **?** | **?** | **?** | **?** | **?** | **?** | ? | ? | ? | ? | ? | ? | ? | **-** | **+** | **-** | **-** | **+** | **-** | ? | ? | - | + | + |
| Sequist, 2005[55] | **-** | **+** | **-** | + | + | + | ? | ? | **+** | **-** | **-** | **-** | **-** | **-** | + | - | - | - | - | - | + | **+** | **+** | **-** | **-** | **-** | **-** | + | - | + | + | + |
| Tierney, 2005[56] | **-** | **+** | **+** | + | + | ? | ? | + | **+** | **-** | **-** | **+** | **-** | **-** | + | - | - | - | - | - | + | **+** | **+** | **-** | **-** | **+** | **-** | ? | + | + | + | + |
| Wolfenden, 2005[57] | **+** | **-** | **-** | ? | ? | ? | ? | ? | **-** | **-** | **-** | **-** | **+** | **-** | + | - | - | - | - | - | - | **-** | **-** | **+** | **-** | **-** | **+** | ? | + | + | + | ? |
| Feldstein, 2006a [22, 41] | **?** | **?** | **?** | ? | ? | ? | ? | ? | **?** | **?** | **?** | **?** | **?** | **?** | - | - | - | - | - | - | + | **-** | **+** | **+** | **-** | **-** | **-** | ? | ? | - | + | + |
| Feldstein, 2006b[40] | **-** | **+** | **?** | + | - | ? | ? | - | **+** | **-** | **-** | **-** | **-** | **-** | + | + | - | - | - | - | - | **-** | **+** | **-** | **-** | **-** | **-** | ? | ? | + | + | ? |
| Judge, 2006[42] | **-** | **+** | **+** | ? | - | - | - | + | **-** | **+** | **-** | **-** | **-** | **-** | + | - | - | - | - | - | - | **-** | **+** | **+** | **+** | **-** | **-** | - | - | + | + | + |
| Kattan, 2006[43] | **+** | **-** | **-** | - | - | - | - | + | **-** | **+** | **-** | **-** | **-** | **-** | - | - | - | - | - | - | + | **-** | **+** | **-** | **-** | **-** | **-** | + | + | ~ | + | + |
| Kuilboer, 2006[44] | **-** | **+** | **-** | ? | ? | + | ? | + | **+** | **-** | **-** | **-** | **-** | **-** | + | - | - | - | - | - | - | **-** | **+** | **-** | **-** | **-** | **-** | + | + | + | + | + |
| Lester, 2006[45, 59] | **-** | **+** | **+** | + | - | - | - | + | **+** | **-** | **-** | **-** | **-** | **-** | - | + | - | - | - | - | - | **-** | **+** | **-** | **-** | **-** | **-** | + | + | - | + | + |
| Palen, 2006[47] | **-** | **+** | **+** | ? | ? | ? | ? | ? | **+** | **-** | **-** | **-** | **-** | **-** | + | - | - | - | - | - | - | **-** | **+** | **-** | **-** | **-** | **-** | + | + | + | + | ? |
| Paul, 2006[48] | **+** | **-** | **-** | ? | - | - | - | + | **-** | **+** | **-** | **-** | **-** | **-** | + | - | - | - | - | - | - | **-** | **+** | **-** | **-** | **-** | **-** | + | - | + | + | + |
| Davis, 2007[32] | **-** | **+** | **+** | ? | ? | ? | ? | + | **+** | **-** | **-** | **-** | **-** | **+** | + | - | + | - | - | - | - | **+** | **+** | **-** | **-** | **-** | **+** | + | + | + | + | + |
| Heidenreich, 2007[33] | **+** | **-** | **-** | ? | ? | ? | ? | ? | **-** | **-** | **-** | **-** | **-** | **+** | + | - | - | - | - | - | - | **-** | **+** | **+** | **-** | **-** | **+** | + | - | + | + | + |
| Martens, 2007[33, 46] | **-** | **+** | **+** | + | + | ? | ? | + | **+** | **-** | **-** | **+** | **-** | **-** | + | - | - | - | - | - | - | **-** | **+** | **-** | **-** | **-** | **-** | ? | + | + | + | + |
| Peterson, 2007[35] | **-** | **-** | **+** | ? | + | + | - | ? | **-** | **-** | **-** | **+** | **-** | **-** | + | - | - | - | - | - | - | **+** | **+** | **+** | **-** | **+** | **-** | + | + | + | + | + |
| Raebel, 2007a[37] | **+** | **-** | **-** | ? | + | ? | ? | ? | **-** | **-** | **+** | **-** | **-** | **+** | - | - | - | - | - | - | + | **-** | **-** | **-** | **-** | **+** | **-** | ? | ? | - | + | + |
| Raebel, 2007b[36] | **+** | **-** | **-** | ? | ? | ? | ? | ? | **-** | **-** | **+** | **-** | **-** | **-** | + | - | - | - | - | - | + | **-** | **-** | **-** | **-** | **+** | **-** | ? | ? | - | + | + |
| Thomson, 2007[38] | **+** | **-** | **-** | + | + | - | - | + | **-** | **-** | **-** | **+** | **-** | **-** | + | - | - | - | - | - | - | **-** | **+** | **-** | **-** | **-** | **-** | + | + | + | + | + |
| Verstappen, 2007[39] | **+** | **-** | **-** | ? | ? | ? | ? | ? | **-** | **+** | **-** | **+** | **-** | **-** | + | - | - | - | - | - | - | **-** | **+** | **-** | **-** | **-** | **-** | + | ? | - | + | + |
| Gurwitz, 2008[25] | **-** | **+** | **+** | + | - | - | - | ? | **-** | **-** | **-** | **+** | **-** | **-** | + | - | - | - | - | - | - | **-** | **+** | **+** | **+** | **-** | **-** | - | - | + | - | - |
| Hicks, 2008[26] | **-** | **+** | **?** | ? | ? | ? | ? | ? | **+** | **-** | **-** | **-** | **-** | **-** | + | - | - | - | - | - | + | **-** | **+** | **+** | **-** | **-** | **-** | ? | + | + | + | ? |
| Javitt, 2008[27] | **+** | **-** | **-** | ? | ? | ? | ? | ? | **-** | **-** | **-** | **-** | **-** | **+** | - | - | - | - | + | + | - | **-** | **+** | **+** | **-** | **-** | **-** | ? | ? | - | + | ? |
| Matheny, 2008[28] | **-** | **+** | **-** | + | - | - | - | + | **+** | **-** | **-** | **-** | **-** | **-** | + | - | - | - | - | - | - | **-** | **+** | **-** | **-** | **-** | **-** | + | + | + | + | + |
| Quinn, 2008[29] | **+** | **-** | **-** | ? | + | ? | ? | + | **-** | **-** | **-** | **-** | **+** | **+** | - | + | - | - | - | - | - | **-** | **+** | **-** | **-** | **-** | **-** | + | + | - | + | + |
| Reeve, 2008[30] | **+** | **-** | **-** | + | - | - | - | - | **-** | **-** | **-** | **+** | **-** | **-** | + | - | - | - | - | - | - | **-** | **-** | **-** | **-** | **+** | **-** | ? | - | + | + | - |
| Van Wyk, 2008[31] | **-** | **+** | **+** | + | + | ? | ? | + | **+** | **-** | **-** | **+** | **-** | **-** | + | - | - | - | - | - | - | **-** | **+** | **-** | **-** | **-** | **-** | + | + | + | + | + |
| Bertoni, 2009[16, 21] | **+** | **-** | **-** | + | + | + | - | ? | **-** | **-** | **-** | **+** | **-** | **-** | - | - | + | - | - | - | - | **-** | **+** | **+** | **+** | **-** | **-** | - | + | + | + | + |
| Field, 2009[17, 24] | **-** | **+** | **+** | + | + | + | - | ? | **+** | **-** | **-** | **-** | **-** | **-** | + | - | - | - | - | - | - | **-** | **+** | **-** | **-** | **-** | **-** | + | - | + | + | + |
| Fortuna, 2009[18] | **-** | **+** | **+** | + | - | - | - | + | **+** | **-** | **-** | **-** | **-** | **-** | + | - | - | - | - | - | - | **-** | **+** | **+** | **+** | **-** | **-** | + | ? | + | + | + |
| Gilutz, 2009[19] | **+** | **-** | **-** | ? | ? | ? | ? | ? | **-** | **+** | **-** | **-** | **-** | **-** | - | - | - | - | - | - | + | **-** | **+** | **-** | **-** | **-** | **+** | + | + | + | + | + |
| Lo, 2009[20] | **-** | **+** | **+** | + | - | + | ? | ? | **+** | **-** | **-** | **-** | **-** | **-** | + | - | - | - | - | - | - | **+** | **+** | **+** | **+** | **-** | **-** | - | - | + | + | + |
| Terrell, 2009[23] | **-** | **+** | **+** | + | + | - | - | + | **-** | **-** | **-** | **+** | **-** | **-** | + | - | - | - | - | - | - | **+** | **+** | **-** | **-** | **-** | **-** | - | - | + | + | + |

Abbreviations: CCDSS, computerized clinical decision support system; CPOE, computerized physician order entry system; EMR, electronic medical record; PDA, personal digital assistant.

^a^Symbol key: +, characteristic present; -, characteristic absent; ~, characteristic sometimes present; ?, unstated or uncertain.
