# Supplementary material for: Computerized clinical decision support systems for drug prescribing and management: A decision-maker-researcher partnership systematic review
Source: Implement Sci. 2011 Aug 3;6:89. doi: 10.1186/1748-5908-6-89 (PMC3179735; doi:10.1186/1748-5908-6-89)
Supplement: Additional file 3 — Study characteristics for trials of drug prescribing. Study characteristics of the included studies. [file 1748-5908-6-89-S3.DOCX]

**Additional file 3, Table S3. Study characteristics for trials of drug prescribing^a^**

| **Study**  **(country)** | **Methods score ^b^** | **Funding source** | **Indication** | **No. of practitioners / patients** | **Setting ^c^ (No. of clinics / sites)** | **CCDSS intervention** | **Comparison** |
| --- | --- | --- | --- | --- | --- | --- | --- |
| **Studies of drug-only interventions** | | | | | | | |
| Field, 2009[17, 24], Canada | 7 | Public | Alerts to promote appropriate drug prescribing and monitoring for patients with renal insufficiency in long-term care. | 10 / 833 | •Long term care (includes nursing home) (1/1) | CCDSS embedded in the order-entry system component of the EMR provided alerts regarding maximum medication dosages and frequencies of administration, inappropriate medications, and missing creatinine clearance results or weights required to calculate appropriate dosages. Alerts were triggered and displayed on the order screen upon initial ordering of a medication for patients with renal insufficiency and could be ignored. | Order entry system displayed current creatinine clearance information with no further recommendations upon drug ordering. |
| Fortuna  2009[18]  USA | 10 | Public | Reminders to consider cost when prescribing hypnotics for adults in primary and urgent care. | 257 / ... | •Academic centre •Subspecialty clinic •Primary care (14/...) | CCDSS triggered an alert when physicians, nurse practitioners, or physician assistants entered new prescription for any of the specified drugs in the EHR-integrated electronic prescribing system. Alerts were based on Harvard Vanguard Medical Associates Pharmaceutical and Therapeutics Committee guidelines and recommended alternative medications (zolpidem, trazodone), linked to evidence summaries, provided co-payment and prescribing information, and provided patient education materials about insomnia and sleep hygiene. Alerts were randomly combined with group education or no additional education. | Usual care with alerts indicating only the drug co-payment status. |
| Lo  2009[20]  USA | 10 | Public | Reminders to order laboratory tests when prescribing new medications in primary care. | 366 / 2765 | •Primary care •Community-based clinic (22/1) | CCDSS generated a non-interruptive alert for missing baseline lab test when physicians ordered new medications on-line. Alerts displayed an on-screen warning in a reserved area of the screen. Providers did not have to act upon or acknowledge notifications to complete medication requests. | Usual care |
| Terrell  2009[23]  USA | 9 | Public | Alerts to avoid inappropriate prescriptions in geriatric outpatients during discharge from emergency care. | 63 / 5162 | •Academic centre •Emergency Department (1/1) | CCDSS data was only provided when a physician in the intervention group attempted to prescribe one of the nine targeted potentially inappropriate medications in patients aged 65 and older who were being discharged from the ED. The system provides either an option to order a recommended alternative therapy or to reject the recommendation. When the latter option was chosen, a second menu was displayed to query the most important reason for rejecting the CCDSS recommendation. | Usual care |
| Gurwitz  2008[25]  USA & Canada | 7 | ... | Prevention of drug-related adverse events in long-term care. | 37 / 1118 | •Academic centre •Long term care (includes nursing home) (2/2) | CPOE-embedded CCDSS displayed evidence-based alerts for potential serious drug interactions in a pop-up box when prescribers (physicians, nurse practitioners, or physician assistants) ordered targeted drugs. Alerts did not require specific action. Some alerts were unnecessary as the CCDSS could not distinguish different forms or strengths of drugs. | Usual care; alerts were not issued |
| Hicks  2008[26]  USA | 7 | Public | Management of hypertension in adults in primary care. | ... / 2027 | •Academic centre •Hospital inpatients •Hospital outpatients •Primary care •Community-based clinic (14/...) | CCDSS generated reminders of hypertension treatment recommendations and displayed them to clinicians at patient visits as part of main EMR screen. Paper version of reminders could be printed. 1 of the 7 clinics in the CCDSS group was also randomized to receive interval visits from a nurse practitioner. | Usual care. Same reminders were triggered but were not delivered to clinicians. 1 of the 7 clinics in the usual care group was also randomized to receive interval visits from a nurse-practitioner. |
| Matheny  2008[28]  USA | 8 | Public | Routine medication laboratory monitoring in primary care. | 303 / 1922 | •Academic centre •Hospital outpatients •Primary care •Community-based clinic (20/20) | CCDSS-generated reminders for laboratory testing (potassium, creatinine, liver or thyroid function, and therapeutic drug levels) appeared on EHRs during visits of patients who were on an included medication for ≥ 365 days with no relevant laboratory test in the past 365 days. | Usual care |
| Reeve  2008[30]  USA | 8 | ... | Use of aspirin in diabetic adults in primary care. | 150 / 258979 | •Other (52/52) | CCDSS, incorporated into pharmacy dispensing software, displayed pop-up prompts to pharmacists when they dispensed oral hypoglycaemic agents. Prompts reminded pharmacists to discuss aspirin therapy with patients potentially eligible for low-dose aspirin for prevention of cardiovascular disease. Pharmacists had to respond to prompts (dismiss, print patient leaflet, or view material and protocol for recommended interventions). During the 1st 3 weeks of the study, 15 of 31 pharmacies also had experienced pharmacists familiar with the dispensing system as observers to assist with documentation. | Prompts were not activated. During the 1^st^ 3 weeks of the study, 7 of 21 pharmacies had experienced pharmacists familiar with the dispensing system as observers to assist with documentation. |
| Davis  2007[32]  USA | 9 | Public | Appropriate prescribing for upper respiratory tract infections in paediatric outpatients. | 44 / 12195 | •Hospital outpatients •Primary care (2/2) | Physicians used an electronic prescription writer on 1 of several computer work stations or wireless hand-held computers to prescribe antibiotics (including selection of indication and treatment duration). CCDSS then displayed evidence-based data relating to the prescription. Full articles or article abstracts were available if requested. | Usual care |
| Heidenreich  2007[33]  USA | 7 | Public | Prescription of β-blockers for inpatients and outpatients with reduced LVEF. | 50 / 1546 | •Academic centre •Hospital inpatients •Hospital outpatients •Subspecialty clinic (3/...) | CCDSS-generated reminders were automatically printed in echocardiography reports of patients with LVEF <45%. The reminder noted that β-blockers improve survival in patients with reduced LVEF, provided initial doses for carvedilol and metoprolol, and recommended cardiology follow-up for patients with New York Heart Association (NYHA) class III or IV symptoms. | Usual care; reminders were not included in reports. |
| Martens  2007[34, 46]  The Netherlands | 9 | Private | Reminders for appropriate use of antibiotics, management of asthma/COPD and dyslipidaemia. | 53 / 3496 | •Academic centre •Primary care •Solo practice (23/...) | CCDSS generated 1 of 2 types of reminders: a) antibiotic/asthma/COPD prescriptions, or b) cholesterol-lowering drug prescriptions. Reminders were based on evidence-based prescribing guidelines and patient data stored in the general practitioners medical information system; the system included a computerized prescription module. | Physicians in the antibiotic/asthma/  COPD reminder group acted as controls for the cholesterol-lowering drug reminder group and vice versa. |
| Peterson  2007[35]  USA | 4 | Private | Dosing advice for high-risk drugs in geriatric patients in a tertiary care academic health centre. | 778 / 2981 | •Academic centre (1/...) | CCDSS provided initial dose advice for sedatives, neuroleptics, anti-emetics, and skeletal muscle relaxants and discouraged prescription of contraindicated drugs for patients ≥65 years old in emergency rooms, intensive care units, and subacute units. Practitioners were not prevented from selecting higher doses than recommended. | Control group not specified (probably usual care with no CCDSS recommendations) |
| Raebel  2007a[37]  USA | 8 | Public, Private | Alerts for potentially inappropriate prescriptions in ambulatory geriatric patients. | ... / 59680 | •Other •Primary care •Community-based clinic (21/21) | CCDSS, as part of the Pharmacy Information Management System (PIMS) linked prescription and age information (electronically obtained from admin and EMR/CPOE databases) and automatically alerted pharmacists when a patient ≥65 years of age was newly prescribed 1 of 11 potentially inappropriate medications. The alert did not allow the prescription label to print until the pharmacist determined whether the prescription should be dispensed. If a safer drug was available, the pharmacist consulted with the prescribing physician by telephone. The targeted medication list was developed by pharmacists and physicians. | Usual care |
| Raebel  2007b[36]  USA | 7 | Public, Private | Alerts to avoid teratogenic drugs in pregnant ambulatory patients. | ... / 11100 | •Hospital outpatients •Primary care •Community-based clinic (.../...) | CCDSS, as part of the Pharmacy Information Management System (PIMS), linked prescription and pregnancy information (electronically obtained from admin and EMR/CPOE databases) and automatically alerted pharmacists when a pregnant patient was prescribed US FDA category D or X medications. The CCDSS did not allow the prescription label to print until the pharmacist determined whether the prescription should be dispensed. Pharmacists consulted with prescribing physicians by telephone to develop plan to resolve alerts. | Usual care |
| Thomson  2007[38]  UK | 8 | Public | Treatment decisions about warfarin or aspirin therapy for patients with atrial fibrillation in primary care. | 2 / 109 | •Other •Academic centre (2/2) | CCDSS presented information to patients about warfarin treatment, including individualised information about benefits and potential harms. The CCDSS risk communication screen, presented information graphically and numerically, and was followed by a shared decision-making component for patients and practitioners. | Practitioners used evidence-based paper guidelines to make treatment recommendations. |
| Verstappen  2007[39]  The Netherlands | 6 | ... | Management of methotrexate for early rheumatoid arthritis in adult outpatients. | ... / 299 | •Academic centre •Hospital outpatients •Subspecialty clinic (6/1) | CCDSS used information on swollen joint count, tender joint count, erythrocyte sedimentation rate, and visual analogue scale for general well-being to determine whether criteria of response to treatment was met. Changes to treatment were made based on response to treatment according to algorithm. Patients attended outpatient clinic every 4 weeks. | Usual care |
| Feldstein  2006a[22, 41]  USA | 10 | Public, Private | Laboratory monitoring for initiating treatments with targeted medications in adult outpatients. | 200 / 961 | •Primary care (15/15) | 3 intervention groups: EMR, automated voice message (AVM), and pharmacy team outreach (PTO). CCDSS initiated specific baseline laboratory monitoring reminders for patients with new prescriptions for any of 10 study medications or medication classes. Reminders were delivered at baseline and 9 to 10 days later for nonrespondents. EMR reminders were sent electronically to practitioners from the chair of patient safety committee. AVM reminders were delivered via recorded telephone messages to patients, prompting them to have preordered tests completed. PTO group reminders were delivered to patients by telephone from pharmacy nurses who indicated preordered tests could be completed at designated labs. | Usual care |
| Judge  2006[42]  USA | 8 | Public | Safety of medication prescribing in a long-term care setting. | 27 / 445 | •Academic centre •Long term care (includes nursing home) (1/1) | CCDSS displayed evidence-based real-time alerts in a pop-up box on the CPOE system when prescribers entered drug orders that posed a potential risk, required monitoring for adverse events, or needed action to prevent adverse events. The 41 potential alerts were informational and did not require specific actions. | Alerts generated but not displayed to prescribers |
| Kattan  2006[43]  USA | 8 | Public | Management of drug therapy in severe asthma in paediatric outpatients. | 435 / 937 | •Hospital outpatients •Solo practice •Community-based clinic  •Primary care (.../7) | Information was collected from each child’s caretaker using a standardised computer-assisted interview every 2 months. The CCDSS used this information and national guidelines to generate a single-page feedback letter that was mailed directly to the child's primary care physician. The letter included a colour photograph of the child, identifying information, details about medication use, asthma symptoms, and health service use, and a 1-sentence treatment recommendation to step up, step down, or don’t change medications | Usual care. Data were also collected from child caretakers bimonthly but letters were not sent to physicians. The information from the calls was used to determine what recommendation would have been generated. |
| Palen  2006[47]  USA | 9 | Public | Reminders for laboratory monitoring based on medication orders in primary care. | 207 / 26586 | •Primary care (16/15) | CCDSS was integrated with EMR and CPOE systems and generated nonintrusive alert messages recommending baseline and ongoing laboratory monitoring when physicians entered orders for selected medications. | Usual care |
| Paul  2006[48]  Italy, Germany and Israel | 10 | Public | Management of antibiotic treatment in hospital inpatients. | ... / 2326 | •Academic centre •Hospital inpatients (15/3) | By imputing variables that significantly influence the probability of pathogens, physicians used the TREAT CCDSS to assess the probability of infection, pathogen distribution, mortality and antibiotic coverage, and prescribe empirical antibiotic treatment for microbiologically documented infections. | In control wards only observation and data collection was conducted and physicians could not access the CCDSS. |
| Derose  2005[50]  USA | 7 | Private | Prescription of ACE-Is, angiotensin receptor blockers and statins in outpatients with diabetes or atherosclerosis. | 1089 / 8557 | •Hospital outpatients •Subspecialty clinic •Primary care (.../...) | CCDSS generated recommendations for cardiovascular medications (ACE-Is or statins) in patients at high-risk for cardiovascular disease. A single-page patient summary sheet, including the recommendations, was faxed to physicians on the morning of a patient visit and attached to the patient’s medical chart. | Usual care. Physicians were faxed the patient summary sheet without recommendations. |
| Heidenreich  2005[51]  USA | 6 | ... | Prescription of ACE-Is or appropriate alternative treatment for inpatients and outpatients with reduced LVEF. | ... / 600 | •Academic centre •Community-based clinic (1/1) | CCDSS-generated reminders were automatically printed in echocardiography reports of patients with ejection fraction <40%. The reminder noted that ACE-Is improve survival in patients with ejection fraction ≤40% and provided a goal dose for lisinopril and fosinopril. | Usual care; reminders were not included in reports. |
| Raebel, 2005[54],  USA | 8 | Public, Private | Laboratory monitoring for initiating treatments with targeted medications in adult outpatients. | ... / 400000 | •Other (.../...) | CCDSS automatically alerted pharmacists at a call centre when targeted medications were ordered for patients who had not completed all pre-determined laboratory tests. Pharmacists reminded patients to obtain laboratory test(s) if previously ordered by physicians or ordered tests accordingly. Pharmacists notified prescribing clinicians of abnormal lab results in writing or by telephone (if urgent). | Usual care |
| Krall  2004[58]  USA | 8 | ... | Use of low dose aspirin therapy in primary care. | 100 / 10972 | •Hospital outpatients •Primary care (.../...) | CCDSS automatically alerted clinicians (physicians, osteopaths, nurse practitioners, or physician assistants) in a pop-up window when certain components of EMRs of patients eligible for aspirin therapy were accessed. Eligible patients were identified by off-line data processing and flagged. Clinicians had to respond to the alert by indicating whether aspirin was prescribed or there was an exclusion/contraindication, or postpone the alert. | Usual care |
| Ansari  2003[61]  USA | 7 | Public | Use of β-blockers for patients with heart failure in primary care. | 74 / 169 | •Academic centre •Primary care •Community-based clinic (1/1) | Providers received a list of heart failure patients who were candidates for β-blocker therapy. CCDSS generated computer alerts for these patients when providers accessed their EMRs during the first 2 visits after randomization. Letters were also sent to the patients advising them to discuss β-blocker therapy with their primary care provider. Providers also received education on β-blocker use in heart failure patients and had access to guidelines on β-blocker initiation and uptitration. | All providers received education on β-blocker use in heart failure patients and had access to guidelines on β-blocker initiation and uptitration. Two control groups: C1. Provider education only. C2. Nurse facilitator group. A study nurse practitioner, supervised by 2 cardiologists, initiated, titrated, and stabilised patients on β-blockers. Patient care was then returned to primary care providers. |
| Filippi  2003[62]  Italy | 7 | ... | Prescribing of anti-platelet medications to diabetic primary care patients. | 300 / 15343 | •Primary care (.../...) | CCDSS was integrated into a standard clinical practice management system, and displayed an electronic reminder when general practitioners opened medical records of diabetic patients ≥ 30 years of age. Physicians could deactivate the reminder. A letter summarizing practice guidelines, including the benefits of anti-platelet drugs in high-risk diabetics, was also sent to practitioners. | Usual care plus the letter summarizing practice guidelines |
| Tamblyn  2003[65]  Canada | 7 | Public, Private | Alerts to avoid inappropriate drug treatments in geriatric outpatients in primary care. | 107 / 12560 | •Primary care (.../...) | Physicians were given a computer, printer, health-record software that documented patient health problems and medications, and dial-up internet access. Trained personnel abstracted patient health problems from physician charts using standardised forms and entered data in the CCDSS. Physicians accessed drug prescribing data for patients through a dedicated computer link to the drug insurance program, and the CCDSS generated alerts for physicians when any of 159 clinically relevant prescribing problems were identified. Alerts identified the problem, possible consequences, and suggested alternative therapies. They were displayed when an electronic chart was opened, health or prescription data were recorded in the chart, or prescription data were downloaded from the insurance provider. | Usual care |
| Weir  2003[67]  UK & Germany | 8 | Public | Prognostic assessments and recommendations for antiplatelet and anticoagulant drugs following acute ischemic stroke or TIA in inpatients and out-patients. | ... / 1952 | •Hospital inpatients •Hospital outpatients (16/16) | CCDSS used patient’s history and clinical findings to estimate the risk of recurrent ischemic stroke, haemorrhagic stroke, MI, or other ischemic or haemorrhagic complications associated with each of 6 possible antiplatelet or anticoagulant therapy. The estimated event rates were provided in a graph of total ischemic event risk and total haemorrhagic event risk which was placed in the patient record for medical staff. | Usual care |
| Zanetti[68]  2003  USA | 8 | Public | Redosing of prophylactic antibiotics during prolonged cardiac surgery. | ... / 447 | •Academic centre •Hospital inpatients (1/1) | CCDSS provided an automated audible alarm and visual intraoperative alert on the operating room computer console for physicians to redose prophylactic antibiotics during cardiac surgery at 225 minutes after administration of preoperative antibiotics. A reply was required to clear the display. If planned redosing was indicated, a new alarm and alert was issued after 30 minutes and the circulating nurse was required to indicate whether a follow-up dose of antibiotics had been administered. | Usual care |
| Christakis  2001[73]  USA | 5 | Public | Use of antibiotics for otitis media in paediatric outpatients. | 38 / 488 | •Other •Academic centre •Hospital outpatients •Primary care (1/1) | Providers (residents, nurse practitioners, and attending physicians) used an electronic prescription writer. When antibiotics were ordered, the CCDSS displayed evidence-based data relating to the selected antibiotic, indication for treatment, and proposed duration of treatment. Full articles or article abstracts were available if requested. | Usual care |
| Rossi  1997[81]  USA | 9 | ... | Reminders to modify drug therapy in hypertensive outpatients receiving calcium channel blockers. | 71 / 719 | •Academic centre •Subspecialty clinic (1/1) | CCDSS automatically generated reminders which were placed in patient charts by the clinic pharmacist and attached to the medication refill forms given to primary care providers. The reminder highlighted the prescription and offered alternative drugs and doses to calcium channel blockers. | Usual Care |
| Rotman  1996[83]  USA | 7 | Public | Reminders to substitute less expensive medications for adult outpatients. | 37 / ... | •Academic centre •Hospital outpatients •Primary care (1/1) | CCDSS was accessed through a physician workstation, included a drug ordering module, and provided alerts to physicians for suggested drug substitutions to reduce costs and prevent adverse drug interactions. It used an internal knowledge base and data uploaded from the hospital information system and allowed users to track medications, problems, and laboratory values in a graphical format that displayed changes over time. | Usual care |
| McDonald  1980[87]  USA | 5 | Public | Detection and management of medication-related problems in outpatients. | 31 / ... | •Academic centre •Hospital outpatients (1/1) | Computerized medical record system used patient data and 410 physician-developed rules, mostly related to use and follow-up of medications, to produce reports for physicians at patient visits. Reports included patient medical history and management reminders for physicians, with or without literature references. | Computer produced reminders but these were not provided to physicians. |
| Coe  1977[88]  USA | 4 | Public | Medication management of hypertension in patients attending hypertension clinics. | ... / 116 | •Academic centre •Subspecialty clinic (2/2) | CCDSS created a compact sequential record of all visits, including a graphic display of blood pressure and drugs in use and provided physicians with hypertension treatment recommendations based on an adaptive algorithm. Physicians were free to follow or reject these recommendations. | Usual care |
| McDonald  1976[89]  USA | 2 | ... | Use of laboratory tests to detect potential medication-related events in adults attending a diabetes clinic. | ... / 226 | •Academic centre •Subspecialty clinic (1/1) | CCDSS generated protocol-driven recommendations for repeat laboratory tests and treatment changes based on EMR data, including past lab results, medications prescribed, and time since previous tests. Recommendations were printed as part of patient reports and placed at the front of patient charts before visits. | Usual care |
| **Studies of multi-faceted interventions** | | | | | | | |
| Bertoni  2009[16, 21]  USA | 9 | Public | Guideline-consistent screening and treatment of dyslipidaemia in primary care. | ... / 3821 | •Primary care (59/59) | CCDSS ran on personal digital assistants (PDAs) given to providers (physicians, physician assistants, and nurse practitioners) in the intervention group. CCDSS generated a 1-page report summarizing patient data, LDL-cholesterol level goals, and treatment recommendations, based on National Cholesterol Education Program Third Adult Treatment Panel (ATP III) guidelines. Providers also received print copies of guidelines, education, and academic detailing. | Comparison group were given automatic blood pressure measurement devices, print copies of guidelines, education, and academic detailing. |
| Gilutz  2009[19]  Israel | 7 | Public | Lipid monitoring and treatment of patients with known coronary artery disease (CAD) in primary care. | 600 / 7448 | •Primary care •Community-based clinic (112/112) | CCDSS collected data from 3 databases (discharge and diagnosis; laboratory; and pharmacy) and automatically generated reminders for management of dyslipidaemia in patients with coronary artery disease based on National Cholesterol Education Program-III and Israeli guidelines. The patient-specific reminders were mailed to physicians and nurses at primary care clinics. The reminders indicated the patient's risk factors, lipoprotein values, and know medications and recommended lipid lowering drug treatment if appropriate. Physicians and nurses could accept or reject CCDSS recommendations. | Usual care |
| Javitt  2008[27]  USA | 6 | ... | Detecting and correcting medical errors in a health maintenance organization setting. | 1378 / 49988 | •Hospital inpatients •Hospital outpatients •Primary care •Solo practice (1/...) | CCDSS collected information on patients > 11 years of age from billing records, lab feeds, and pharmacies, created a virtual EMR, and applied decision rules to produce patient-specific care considerations (CCs) if indicated. CCs fell into three categories (stop a drug, do a test, and add a drug) and included 3 severity levels. Each CC included issues of concern, suggested actions, and relevant literature citations. CCDSS-associated physicians reviewed each CC. Those that passed review were forwarded to patient physicians by telephone (level 1 severity) or to HMO nurses (level 2 or 3 severity), who reviewed them and could choose to fax them to patient's physicians. | Usual care |
| Quinn  2008[29]  USA | 6 | Private | Diabetes management in primary care patients with type 2 diabetes. | 26 / 30 | •Subspecialty clinic •Primary care •Community-based clinic (3/...) | WellDoc System (WDS) is a cell phone-based diabetes management software system that incorporates real-time patient coaching based on blood glucose (BG) measures taken with a bluetooth-adapted One Touch Ultra™ BG meter. The WDS also provided feedback for practitioners, including patient BG logbooks with automated analysis and suggested medication changes. Patients were provided with cell phones and adapted BG meters. | Usual provider care patients were also given One Touch Ultra™ BG meters (LifeScan, Milpitas, CA) and asked to fax or call in their BG logbooks to their providers for review. |
| van Wyk  2008[31]  The Netherlands | 10 | Public | Screening and treatment of dyslipidaemia in primary care. | 80 / 92054 | •Primary care •Solo practice •Community-based clinic (38/38) | There are 2 versions of the CCDSS: on-demand and automatic alerting, both integrated with an EHR and based on guidelines from the Dutch College of General Practitioners. The CCDSS generated patient-specific recommendations for preventative care and displayed them on an interactive patient overview screen in the EHR. With the on-demand CCDSS, users had to actively initiate the overview screen. With the automatic alerting CCDSS, recommendations were automatically displayed to users. | Usual Care |
| Feldstein  2006b[40]  USA | 8 | Public | Osteoporosis screening and treatment in at-risk women in primary care. | 159 / 311 | •Primary care (15/1) | Patient-specific advice, based on guidelines for osteoporosis management (ordering a BMD measurement and prescribing osteoporosis medication), was delivered via EMR to primary care physicians. Providers who had not ordered a BMD measurement or medication within 3 months of first reminder received a second reminder. In 1 of 2 intervention arms, patients also received a mailed reminder with educational materials. | Usual care |
| Kuilboer  2006[44]  The Netherlands | 10 | Public | Monitoring and treatment of asthma and COPD in daily practice in primary care. | 40 / 156772 | •Primary care •Solo practice (32/32) | CCDSS uses data in EHR and clinical guidelines to provide feedback on treatment to physicians for patients with asthma or COPD. | Usual care |
| Lester  2006[45, 59]  USA | 8 | Private | Management of dyslipidaemia in primary care. | 14 / 235 | •Primary care (1/1) | CCDSS identified high-risk patients with elevated LDL cholesterol levels (> 100mg/dL 6 to 24 mo before study initiation) for cholesterol management and sent a single, customised email to physicians. Via emails, users could review patient information and, with a single click, generate a statin prescription, repeat fasting lipid profile, or decline change in medical management. CCDSS recommendations were based on evidence-based guidelines. Existing EHRs were automatically updated. | Usual care |
| Cobos  2005[49]  Spain | 10 | Private | Management of dyslipidaemia in adults in primary care. | ... / 2221 | •Primary care (42/44) | CCDSS generated recommendations for hypercholesterolemia therapy, follow-up visit frequency, and laboratory test ordering, based on patient data entered by physicians, including CV risk and LDL cholesterol goals. Recommendations were adapted from the European Society of Cardiology and other societies for Hypercholesterolemia Management (ESCHM) guidelines. Physicians could adopt or ignore the recommendations. The intervention included availability of patient education promotions such as tablecloths and refrigerator magnets. | Usual care |
| Javitt  2005[52]  USA | 6 | Private | Management of patients when care deviates from recommended evidence-based practices in primary care. | ... / 39462 | •Primary care (.../...) | CCDSS scanned administrative data and used > 1000 decision rules to detect potential deviations from recommended care practices. Deviations triggered recommendations and supporting literature, which were sent to treating physicians. | Data about patients in the control group also triggered recommendations, but these were not sent to physicians. |
| Plaza  2005[53]  Spain | 9 | Private | Management of asthma in primary care. | 20 / 198 | •Subspecialty clinic •Primary care (.../5) | CCDSS provided recommendations to general practitioners and pneumologists for asthma treatment based GINA guidelines. GINA based intervention included information about chronic inflammatory illness, technique when using an inhaler, maximum expiratory flow (FEM), FEM self-monitoring techniques and GINA recommendations. | Usual care |
| Sequist  2005[55]  USA | 6 | Public | Management of diabetes and coronary artery disease in primary care. | 194 / 6243 | •Academic centre •Hospital outpatients •Primary care •Community-based clinic (20/20) | When clinicians opened patient charts within EMRs, the CCDSS determined whether the patient had received care in accordance with the recommended evidence-based practice guidelines for care of diabetes or coronary artery disease. Appropriate reminders were then displayed on the patient summary screen of the EMR. Physicians could also choose to have the reminders printed. All physicians received electronic reminders for overdue preventive care services. | Electronic reminders were suppressed but printing of paper reminders was an option. All physicians received electronic reminders for overdue preventive care services. |
| Tierney  2005[56]  USA | 9 | Public | Management of asthma and COPD in adults in primary care. | 266 / 706 | •Academic centre •Primary care (4/...) | Existing computer workstations were programmed to provide care suggestions to physicians and pharmacists based on evidence-based guidelines for asthma and COPD management and data in patient EMRs. Physicians received CCDSS-generated care suggestions on paper medication lists at patient visits and on computer workstations when writing orders. Pharmacists received them electronically and could choose to do nothing or discuss suggestions with patients or physicians. They received the same educational material as the control group. | Physicians and pharmacists received a printed summary of asthma and COPD management guidelines and could attend rounds about the guidelines but did not receive care suggestions. |
| Wolfenden  2005[57]  Australia | 7 | Public | Improving smoking cessation in patients attending a noncardiac preoperative clinic. | 18 / 210 | •Academic centre •Hospital outpatients •Subspecialty clinic (1/1) | CCDSS was part of a multi-faceted intervention. CCDSS provided interactive behavioural smoking cessation counselling; written prompts for nurses (n=5) and anaesthetists (n=13) to provide brief cessation advice, preoperative nicotine replacement therapy (NRT) if smoking >10 cigarettes/d, and a prescription for postoperative NRT if smoking >10 cigarettes/d and expect >1d on ward; and tailored self-help material based on patient responses to cessation information provided by the CCDSS. Other elements of the intervention included: identifying opinion leaders, staff involvement in intervention development (establishing consensus), nurse and anaesthetist staff training, and monitoring and feedback of care provision. | Usual care |
| Murray  2004[60]  USA | 5 | Public | Management of hypertension in primary care. | ... / 712 | •Academic centre •Primary care (4/4) | 2x2 factorial trial (physician intervention, pharmacist intervention, intervention for physician and pharmacist, no intervention). Existing computer workstations were programmed to provide treatment suggestions to physicians and pharmacists based on evidence-based guidelines for hypertension management and data in patient EMRs. Physicians received CCDSS-generated care suggestions on paper medication lists at patient visits and on computer workstations when writing orders. Pharmacists received them electronically and could choose to fill the prescription or discuss suggestions with patients or physicians. On-line and printed treatment suggestions were available for all study groups. | CCDSS generated suggestions for both intervention and control groups but they were not displayed for patients in the control group. |
| Tierney  2003[66]  USA | 10 | Public | Management of heart disease in primary care. | 115 / 706 | •Academic centre •Primary care •Community-based clinic (4/...) | 3 intervention groups: physician, pharmacist, or both. All physicians used an EMR system with computerized order entry. Physician intervention: CCDSS generated cardiac care suggestions approved by local cardiologists and general internists and based on EMR data, data entered by physicians after visits, and evidence-based guidelines (Agency for Health Care Policy and Research). Suggestions were printed on the patient encounter form and displayed on physician workstations. Physicians could follow or disregard the suggestions. Pharmacist intervention: CCDSS (Pharmacist Intervention Recording System [PIRS] printed a note (rather than bottle labels) when prescriptions were filled for eligible patients, directed pharmacists to care suggestions in PIRS and provided 3 options for action: fill the prescription as usual, discuss care suggestions with the patient, or contact the physician by telephone or PIRS-facilitated e-mail which would be displayed for the physician at next workstation log in. | Usual care with the same EMR and order entry system but without cardiac care suggestions. Longstanding computer-generated preventive care reminders were presented. |
| Eccles  2002[69, 64]  UK | 10 | Public, Private | Management of asthma and angina in adults in primary care. | ... / 4506 | •Primary care (62/...) | CCDSS provided internally-developed evidence-based guidelines and care suggestions to general practitioners and practice nurses for management of adults with asthma or angina in primary care, based on electronic patient records. CCDSS was triggered when EMRs of eligible patients were opened or a relevant morbidity code was entered. | Physicians receiving asthma guidelines did not receive angina guidelines and vice versa. |
| Flottorp  2002[70, 63]  Norway | 9 | Public | Management of urinary tract infections (UTIs) in women and sore throat in primary care. | ... / ... | •Primary care (142/1) | CCDSS was not described in detail but provided support and reminders during consultations for management of UTIs and sore throats, based on locally-developed guidelines. Guidelines recommended that most patients did not need antibiotics or lab tests for sore throats and antibiotics could generally be used without lab tests in non-pregnant women with UTIs. Patients could be given advice by telephone (except for patients with a UTI who had no previous UTIs). CCDSS was part of a broader intervention that also provided treatment recommendations and patient and provider education material electronically and in print, increased telephone consultation fees, and credited participants with points for continuing medical education. | The group receiving the sore throat intervention served as controls for the group receiving the intervention for UTIs and vice versa. |
| Lesourd  2002[71]  France | 5 | Private | Hormonal ovarian stimulation for infertile women in a teaching hospital. | 4 / 164 | •Academic centre (3/3) | CCDSS used data related to patient fertility, age, and current response to treatment to evaluate likely response of ovaries to FSH stimulation and suggest next steps for treatment, including adjustment of FHS regimen and monitoring, hCG induction of ovulation, or cycle cancellation. If patients did not become pregnant, the CCDSS suggested a protocol for a new treatment cycle based on data entered by clinicians. | Clinician monitoring of ovarian stimulation |
| Selker  2002[72]  USA | 8 | Public | Management of thrombolytic and other reperfusion therapy in acute myocardial infarction. | ... / 1596 | •Academic centre •Hospital inpatients •Primary care (28/28) | Thrombolytic Predictive Instrument (TPI) is an electrocardiograph-based CCDSS. When there is an ST segment elevation on the ECG, TPI prints on ECG text header its prediction of five key outcomes of thrombolytic therapy for acute myocardial infarction patients. | Usual care |
| Dexter  2001[74]  USA | 10 | Public | Preventive therapies in hospital inpatients. | 202 / 3416 | •Academic centre •Hospital inpatients (.../...) | CCDSS provided guideline-based reminders for preventative care procedures to physicians and medical students. | Usual care |
| McCowan  2001[75]  UK | 8 | Public | Management of asthma in primary care. | 46 / 477 | •Primary care (.../...) | CCDSS (Asthma Crystal Byte) used current asthma guidelines and data entered during consultation to provide management recommendations and reminders. Patient-specific self-management plans and advice sheets could be printed for patients. Physicians and practice nurses evaluated the CCDSS. | Usual care. Practices informed they would have to report on patient outcomes after 6 months. |
| Demakis  2000[76]  USA | 7 | Public | Screening, monitoring, and counselling in accordance with predefined standards of care in ambulatory care. | 275 / 12989 | •Other •Academic centre •Hospital outpatients (12/12) | Residents received CCDSS-generated reminders relating to 13 prespecified standards of care in 2 ways. 1) On entering a patient name into a computer terminal in the examining room, applicable reminders were automatically displayed in bold letters. 2) Applicable reminders were printed on the standard patient health summary which is attached to patient charts at visits. | Control residents only received the standard health summaries without the reminders. |
| Hetlevik  1999[77-79]  Norway | 8 | Public | Diagnosis and management of hypertension, diabetes, and dyslipidaemia in primary care. | 56 / 3273 | •Primary care (56/...) | CCDSS provided guidance for diagnosis, history taking, physical exams, tests, and treatment based on Norwegian clinical guidelines for patients with hypertension, diabetes, or hypercholesterolemia in primary care. The CCDSS was external to, but accessible from, the main computerized medical record system and was initiated by the physician at their discretion. | Usual care |
| Overhage  1997[80]  USA | 8 | Public | Corollary orders to prevent errors of omission for tests and treatments in general medicine inpatients. | 92 / 2181 | •Academic centre •Hospital inpatients (1/1) | A rule-based reminder CCDSS determined corollary orders for 87 target orders and displayed these on-line to physicians using the computerized order entry system. Corollary orders could be accepted or rejected by physicians. | Physicians used computerized order entry system but did not receive on-line corollary orders. |
| Overhage  1996[82]  USA | 10 | Public | Compliance with 22 US Preventive Services Task Force preventive care measures for hospital inpatients. | 78 / 1622 | •Academic centre •Hospital inpatients (1/1) | CCDSS was incorporated into the electronic medical record and order-entry system and used data from these sources to generate reminders for 22 preventive care measures. CCDSS ran overnight and provided reminders to physicians in 2 ways: printed at the top of daily patient reports, and displayed at the bottom of the workstation screen in red when physicians entered orders for patients. Physicians could accept or reject orders generated by the reminder program. | Usual care |
| Tierney  1993[84]  USA | 10 | Public | Alerts for drug allergies and drug-drug interactions, and options for cost-effective testing in inpatients. | 276 / 5219 | •Academic centre •Hospital inpatients (6/...) | CCDSS embedded in computerized order entry system displayed item charges, listed the most cost-effective tests and test intervals, and indicated drug allergies and potential interactions, based on data from patient electronic medical records, hospital billing system, and entered by physicians ordering tests. | Manual order writing |
| Mazzuca  1990[85]  USA | 7 | Public | Management of non-insulin dependent diabetes mellitus in outpatients. | 114 / 279 | •Academic centre (4/4) | 3 treatment groups: CCDSS patient-specific reminders + seminar (B); B + seminar-related clinical materials (C); and C + diabetes patient education service (D). CCDSS reminders were generated from the medical record system and placed in patients' clinic records whenever the computer detected history, physical, laboratory, or pharmacy data indicating that a seminar recommendation should be considered. | A 3.5-hour seminar covering blood sugar regulation in non-insulin dependent diabetes mellitus was offered to all physicians. All participants received a course syllabus, key reprints, and a reference book. |
| McAlister  1986[86]  Canada | 7 | Public | Management of hypertension in primary care. | 50 / 2231 | •Primary care (50/...) | 25 practices in each group. Physicians recorded patient-specific data, including information about medications and date of next scheduled visit, on encounter forms after visits with hypertensive patients. Forms were mailed to a central test centre, data entered into a CCDSS, and feedback generated for physicians including a chart of diastolic blood pressure, intra- and inter-practice blood pressure percentile rankings, and treatment suggestions based on the “stepped care” protocol. Appointment reminders were also mailed to patients and if a patient missed the appointment, a reminder letter was sent. | Filled out the same collection forms as the study group and mailed them to the study centre. No feedback/reminder was sent to doctors or patients. |

Abbreviations: ACE-I, angiotensin-converting enzyme inhibitors; AVM, automated voice message; BMD, bone mineral density; CCDSS, computerized clinical decision support system; COPD, chronic obstructive pulmonary disease; CPOE, computerized provider order entry; EHR, electronic health record; EMR, electronic medical record; LDL, low-density lipoprotein; LVEF, left ventricular ejection fraction.

^a^ Ellipses (…) indicate item was not assessed.

^b^ Based on 5 individual items (score 2, yes, 1, partly, and 0, no) and a summed total score (range 0 to 10). Because this review update included only randomized, controlled trials, the total score differs from that reported in the previous version of this review[2]: the item evaluating study type (randomized, quasi-analized, or concurrent controls) has been replaced by one that evaluates use of concealed allocation (concealed, unclear, not concealed).

^c^ Diabetes clinic is an example of a subspecialty clinic
