# Supplementary material for: Computerized clinical decision support systems for drug prescribing and management: A decision-maker-researcher partnership systematic review
Source: Implement Sci. 2011 Aug 3;6:89. doi: 10.1186/1748-5908-6-89 (PMC3179735; doi:10.1186/1748-5908-6-89)
Supplement: Additional file 4 — Results for CCDSS trials of drug prescribing. Details results of the included studies. [file 1748-5908-6-89-S4.DOCX]

**Additional file 4, Table S4. Results for CCDSS trials of drug prescribing**

| **Study** | **Process of care outcomes** | **CCDSS vs. control data** | **Patient outcomes** | **CCDSS vs. control data** | **CCDSS process of care effect^a^** | **CCDSS patient**  **effect^a^** |
| --- | --- | --- | --- | --- | --- | --- |
| **Studies of drug-only interventions** | | | | | | |
| Field  2009[17, 24]  Canada | 1. Number of final drug orders that were appropriate; number of appropriate orders/number of alerts (%), Relative Risk (95% CI). (primary) 1a. Dose 1b. Frequency 1c. Avoid 1d. Missing information **1e. Total**  2. Final orders for drugs that should have been avoided. Number per 1000 patient-days, Rate ratio, (95% CI) (secondary)  3. Number of drug orders that were appropriate by drug; number of appropriate orders / number of alerts (%) (not prespecified); no p values or CIs provided 3a. Allopurinol 3b. Amantadine  3c. Amoxicillin  3d. Cefprozil  3e. Cefuroxime  3f. Cephalexin  3g. Ciprofloxacin  3h. Clarithromycin  3i. Colchicine  3j. Cotrimoxazole  3k. Diclofenac  3l. Digoxin  3m. Famciclovir  3n. Gabapentin  3o. Glyburide  3p. Ibuprofen  3q. Indomethacin  3r. Levofloxacin  3s. Lithium  3t. Loratadine  3u. Meloxicam  3v. Memantine  3w. Metformin  3x. Metoclopropamide  3y. Metronidazole  3z. Nitrofurantoin  3aa.Norfloxacin  3ab. Pentoxifyline  3ac. Pramipexole  3ad. Primidone  3ae. Ranitidine  3af. Tetracycline  3ag. Trimethoprim  3ah. Venlafaxine | 1a. 86/114 (75.4%) vs. 107/134 (79.9%), 0.95 (0.83 to 1.1) 1b. 30/49 (61.2%) vs. 9/35 (25.7%), 2.4 (1.4 to 4.4) 1c. 26/64 (40.6%) vs. 10/65 (15.4%), 2.6 (1.4 to 5.0) 1d. 30/47 (63.8%) vs. 8/23 (34.8%), 1.8 (1.1 to 3.4) 1e. 172/274 (62.8%) vs. 134/257 (52.1%), 1.2 (1.0 to 1.4)  2. 3.5 vs. 5.2, 0.68 (0.45 to 1.0)  3a. 0/0 vs. 1/2 (50%) 3b. 0/2 (0%) vs. 0/3 (0%) 3c. 1/1 (100%) vs. 0/0 3d. 0/0 vs. 1/1 (100%) 3e. 1/1 (100%) vs. 0/0 3f. 16/31 (52%) vs. 3/23 (13%) 3g. 7/7 (100%) vs. 24/26 (92%) 3h. 1/1 (100%) vs. 0/0 3i. 0/0 vs. 2/3 (67%) 3j. 18/21 (86%) vs. 4/10 (40%) 3k. 0/0 vs. 1/5 (20%) 3l. 8/9 (89%) vs. 9/9 (100%) 3m. 4/4 (100%) vs. 0/1 (0%) 3n. 9/10 (90%) vs. 28/28 (100%) 3o. 4/22 (18%) vs. 2/15 (13%) 3p. 0/0 vs. 0/3 (0%) 3q. 1/2 (50%) vs. 0/0 3r. 50/68 (74%) vs. 31/50 (62%) 3s. 1/1 (100%) vs. 6/6 (100%) 3t. 4/5 (80%) vs. 0/2 (0%) 3u. 0/0 vs. 0/5 (0%) 3v. 1/2 (50%) vs. 1/1 (100%) 3w. 10/26 (39%) vs. 3/13 (23%) 3x. 1/2 (50%) vs. 0/0 3y. 4/4 (100%) vs. 1/1 (100%) 3z. 15/26 (58%) vs. 6/32 (19%) 3aa. 0/0 vs. 1/1 (100%) 3ab. 1/1 (100%) vs. 0/0 3ac. 1/1 (100%) vs. 0/0 3ad. 0/1 (0%) vs. 0/0 3ae. 2/4 (50%) vs. 2/7 (29%) 3af. 2/2 (100%) vs. 0/0 3ag. 1/1 (100%) vs. 0/0 3ah. 9/19 (47%) vs. 8/10 (80%) | ... | ... | 0 | … |
| Fortuna  2009[18]  USA | Primary 1. Change in proportion of hypnotic drug prescriptions that were for heavily marketed hypnotics over 1 y.  **1a. Alerts vs. control. Adjusted* RR (95% CI) for change from baseline; ratio of RRs (95% CI).** 1b. Alerts + education vs. control. Adjusted RR (95% CI) for change from baseline; ratio of RRs (95% CI). 1c. Alerts vs. alerts + education. Adjusted RR (95% CI) for change from baseline; ratio of RRs (95% CI).  RR <1 = prescribing decreased; RR>1, prescribing increased. Adjusted for clinician age, gender, full time status, years in practice, degree, and primary care or not. | 1a. 0.97 (0.82 to 1.14) vs. 1.31 (1.08 to 1.60); 0.74 (0.57 to 0.96), *P*=.02 1b. 0.98 (0.83 to 1.17) vs1.31 (1.08 to 1.60); 0.74 (0.58 to 0.97), *P*=.03 1c. 0.97 (0.82 to 1.14) vs. 0.98 (0.83 to 1.17); 1.02 (0.80 to 1.29), *P*=.90 | ... | ... | + | … |
| Lo  2009[20]  USA | **Primary outcome. 1. Rate of ordering appropriate baseline laboratory tests within 14 days of clinical encounter, n/N, %; OR, 95% CI.**  Not prespecified  2. Association between non-interruptive alerts and number of lab tests ordered within 14 days of alert for 11 (of 23) medication classes with >32 orders placed); n/N for both groups combined; OR, 95% CI.  2a. Antimanic agents. 2b. Hydroxymethylglutaryl-CoA reductase inhibitors. 2c. Diuretics. 2d. ACE-Is. 2e. Hypoglycaemic. 2f. Antifungal antibiotics, 2g. Anticonvulsants. 2h. Antiarthritics. 2i. Cardiotonic agents. 2j, Antituberculosis agents. 2k.Angiotensin II receptor antagonists.  Not prespecified  3. Association between non-interruptive alerts and number of lab tests ordered within 14 days of alert for 5 of 12 lab tests with sufficient sample size; n/N for both groups combined; OR, 95% CI. 3a. Alkaline phosphatase. 2b. Alanine aminotransferase 3c. Thyroid stimulating hormone. 3d. Creatinine. 3e. Potassium.  Not prespecified  4. Association between non-interruptive alerts and number of lab tests ordered within 14 days of alert for 3 medications with significant associations (of 70 medications monitored); 95% CI for OR. 4a. Pravastatin. 4b. Atorvastatin. 4c. Lithium. | 1. 689/1685, 41% vs. 771/1988, 39%; 1.048, 0.753 to 1.457, *P*=.782  2a. 24/71; 0.117, 0.016 to 0.858, *P*=.035 2b. 295/1025; 0.654, 0.377 to 1.136, *P*=.132  2c. 404/799; 1.324, 0.866 to 2.023, *P*=.196 2d. 289/621; 1.184 , 0.660 to 2.124, *P*=.571 2e. 82/177; 1.221, 0.662 to 2.252, *P*=.524 2f. 65/106; 0.854, 0.275 to 2.649, *P*=.785 2g. 44/255; 0.591, 0.127 to 2.756, *P*=.503 2h. 25/103; 1.328, 0.564 to 3.129, *P*=.517 2i. 35/56; 0.346, 0.024 to 4.977, *P*=.435 2j. 62/115; 1.964, 0.506 to 7.617; *P*=.329 2k. 53/130; 2.583, 0.821 to 8.131, *P*=.105  3a. 18/82; 0.740, 0.223 to 2.456, *P*=.623 3b. 483/1453; 0.789, 0.502 to 1.242, *P*=.306 3c. 17/56; 0.811, 0.235 to 2.803, *P*=.741 3d. 165/384; 1.267, 0.738 to 2.175, *P*=.392  3e. 744/1526; 1.288, 0.852 to 1.947, *P*=.229  4a. –ve association, OR CI 0.015 to 0.744, *P*=.024 4b. –ve association, OR CI 0.299 to 0.952, *P*=.034  4c. –ve association, OR CI 0.016 to 0.947, *P*=.044 | ... | ... | 0 | ... |
| Terrell  2009[23]  USA | **Primary 1. Number (%) of ED visits by older adults that resulted in prescriptions for one of more of the nine targeted inappropriate medications; odds ratio (95% CI), P-value.**  Secondary 2. Number (%) of all prescribed medications that were potentially inappropriate; odds ratio (95% CI), P-value.  Pre-specified 3. Number of times that each potentially inappropriate medication was initially prescribed (n)/ changed to an alternate treatment (n, %) in the CCDSS group vs. prescribed in the control group (n). 3a. Promethazine  3b.Diphenhydramine 3c. Diazepam 3d. Propoxyphene with acetaminophen  3e. Hydroxyzine 3f. Amitriptyline 3g. Cyclobenzaprine 3h. Clonidine 3i. Indomethacin 3j. All inappropriate medications | 1. 69 (2.6%) vs. 99 (3.9%); 0.55 (0.34 to 0.89), *P=.*02 2. 69 (3.4%) vs. 103 (5.4%); 0.59 (0.41 to 0.85), *P*=.006  3a. 32 / 19 (59%) vs. 40  3b. 22 / 8 (36%) vs. 15  3c. 18 / 5 (28%) vs. 10  3d. 8 / 2 (25%) vs. 9  3e. 15 / 6 (40%) vs. 9  3f. 1 / 0 (0%) vs. 8  3g. 5 / 2 (40%) vs. 7  3h. 3 / 2 (67%) vs. 4  3i. 10 / 5 (50%) vs. 1  3j. 114 / 49 (43%) vs. 103 | ... | ... | + | … |
| Gurwitz  2008[25]  USA & Canada | … | … | 1 y follow-up in 1 of 2 sites and 6 mo follow-up in the 2nd site for 3,803 vs. 3,257 resident-months of observation.  **Primary 1. Number (%) of adverse drug events; rate per 100 resident-months; adjusted rate ratio (95% CI). 1a. All.** 1b. Preventable. 1c. More severe 1d. Preventable more severe. 1e. Less severe. 1f. Preventable less severe.  Analyses not prespecified 2. Number (%) of adverse drug events by event type: all events; preventable events. 2a. Haemorrhagic. 2b. Neuropsychiatric (including oversedation, confusion, hallucinations, and delirium). 2c. Gastrointestinal. 2d. Metabolic or endocrine. 2e. Renal or electrolytic. 2f. Cardiovascular. 2g. Dermatological. 2h. Fall without injury. 2i. Extrapyramidal signs or symptoms. 2j. Syncope or dizziness. 2k. Infection. 2l. Haematological. 2m. Anticholinergic (including dry mouth, dry eyes, urinary retention, and constipation). 2n. Respiratory. 2o. Anorexia. 2p. Functional decline (decline in activities of daily living without other more-specific events). 2q. Fall with injury. 2r. Ataxia or difficulty with gait. 2s. Hepatic.  Analyses not prespecified 3. Number (%) of adverse drug events by drug category: all events; preventable events. 3a. Antiplatelet. 3b. Antipsychotic. 3c. Anticoagulant. 3d. Diuretic. 3e. Anti-infective. 3f. Cardiovascular. 3g. Hypoglycaemic. 3h. Gastrointestinal. 3i. Antidepressant. 3j. Opioid. 3k. Sedative or hypnotic. 3l. Antiepileptic. 3m. Nutrient or supplement. 3n. Steroid. 3o. Anti-Alzheimer’s. 3p. Thyroid. 3q. Digoxin. 3r. Anti-Parkinson’s. 3s. Antihistamine. 3t. Muscle relaxant. 3u. Topical.  3v. Ophthalmic. 3w. Gout. 3x. Antineoplastic. 3y. Respiratory. 3z. Osteoporosis. 3zz. Miscellaneous.  Post-hoc analysis 4. Number (%) of preventable events that could have been prevented as a result of >= 1 alert; rate per 100 resident-months; adjusted rate ratio (95% CI). | 1a. 411 (100%) vs. 340 (100%); 10.8 vs. 10.4; 1.06 (0.92 to 1.23) 1b. 152 (37.0%) vs. 126 (37.1%); 4.0 vs. 3.9; 1.02 (0.81 to 1.30) 1c. 123 (30.0%) vs. 97 (28.5%); 3.2 vs. 3.0; 1.07 (0.82 to 1.40) 1d. 79 (19.2%) vs. 58 (17.1%); 2.1 vs. 1.8; 1.15 (0.82 to 1.61) 1e. 288 (70.1%) vs. 243 (71.5%); 7.6 vs. 7.5; 1.06 (0.89 to 1.26) 1f. 73 (17.8%) vs. 68 (20.0%); 1.9 vs. 2.1; 0.92 (0.66 to 1.28)  2a. 102 (24.8%) vs. 85 (25.0%); 22 (14.5%) vs. 20 (15.9) 2b. 87 (21.2%) vs. 71 (20.9%); 42 (27.6%) vs. 28 (22.2%) 2c. 70 (17.0%) vs. 49 (14.4%); 17 (11.2%) vs. 18 (14.3%) 2d. 43 (10.5%) vs. 32 (9.4%); 24 (15.8%) vs. 13 (10.3%) 2e. 31 (7.5%) vs. 47 (13.8%); 15 (9.9%) vs. 29 (23.0%) 2f. 20 (4.9%) vs. 15 (4.4%); 13 (8.6%) vs. 8 (6.4%) 2g. 9 (2.2%) vs. 14 (4.1%); 0 (0%) vs. 1 (0.8%) 2h. 14 (3.4%) vs. 7 (2.1%); 8 (5.3%) vs. 2 (1.6%) 2i. 12 (2.9%) vs. 7 (2.1%); 6 (4.0%) vs. 1 (0.8%) 2j. 7 (1.7%) vs. 11 (3.2%); 5 (3.3%) vs. 4 (3.2%) 2k. 12 (2.9%) vs. 4 (1.2%); 0 (0%) vs. 0 (0%) 2l. 4 (1.0%) vs. 0 (0%); 1 (0.7%) vs. 0 (0%) 2m. 2 (0.5%) vs. 5 (1.5%); 2 (1.3%) vs. 2 (1.6%) 2n. 2 (0.5%) vs. 5 (1.5%); 1 (0.7%) vs. 3 (2.4%) 2o. 2 (0.5%) vs. 4 (1.2%); 2 (1.3%) vs. 2 (1.6%) 2p. 2 (0.5%) vs. 2 (0.6%); 2 (1.3%) vs. 2 (1.6%) 2q. 2 (0.5%) vs. 1 (0.3%); 2 (1.3%) vs. 1 (0.8%) 2r. 2 (0.5%) vs. 0 (0%); 2 (1.3%) vs. 0 (0%) 2s. 1 (0.2%) vs. 0 (0%); 0 (0%) vs. 0 (0%)  3a. 66 (16.1%) vs. 58 (17.1%); 11 (7.2%) vs. 11 (8.7%) 3b. 52 (12.7%) vs. 40 (11.7%); 25 (16.5%) vs. 13 (10.3%) 3c. 42 (10.2%) vs. 39 (11.5%); 17 (11.2%) vs. 10 (7.9%) 3d. 33 (8.0%) vs. 36 (10.6%); 18 (11.8%) vs. 23 (18.3%) 3e. 38 (9.3%) vs. 30 (8.8%); 1 (0.7%) vs. 7 (5.6%) 3f. 30 (7.3%) vs. 38 (11.2%); 18 (11.8%) vs. 24 (19.1%) 3g. 36 (8.8%) vs. 17 (5.0%); 19 (12.5%) vs. 6 (4.8%) 3h. 39 (9.5%) vs. 11 (3.2%); 9 (5.9%) vs. 5 (4.0%) 3i. 25 (6.1%) vs. 25 (7.4%); 14 (9.2%) vs. 9 (7.1%) 3j. 26 (6.3%) vs. 20 (5.9%); 11 (7.2%) vs. 9 (7.1%) 3k. 17 (4.1%) vs. 23 (6.8%); 10 (6.6%) vs. 12 (9.5%) 3l. 17 (4.1%) vs. 14 (4.1%); 7 (4.6%) vs. 9 (7.1%) 3m. 9 (2.2%) vs. 15 (4.4%); 4 (2.6%) vs. 8 (6.3%) 3n. 12 (2.9%) vs. 6 (1.8%); 1 (0.7%) vs. 0 (0%) 3o. 7 (1.7%) vs. 7 (2.1%); 4 (2.6%) vs. 0 (0%) 3p. 4 (1.0%) vs. 8 (2.3%); 3 (2.0%) vs. 5 (4.0%) 3q. 5 (1.2%) vs. 5 (1.5%); 4 (2.6%) vs. 2 (1.6%) 3r. 6 (1.5%) vs. 3 (0.9%); 4 (2.6%) vs. 1 (0.8%) 3s. 6 (1.5%) vs. 2 (0.6%); 3 (2.0%) vs. 1 (0.8%) 3t. 5 (1.2%) vs. 3 (0.9%); 2 (1.3%) vs. 2 (1.6%) 3u. 3 (0.7%) vs. 1 (0.3%); 2 (1.3%) vs. 0 (0%) 3v. 1 (0.2%) vs. 2 (0.6%); 0 (0%) vs. 0 (0%) 3w. 0 (0%) vs. 3 (0.9%); 0 (0%) vs. 2 (1.6%) 3x. 1 (0.2%) vs. 1 (0.3%); 0 (0%) vs. 0 (0%) 3y. 1 (0.2%) vs. 1 (0.3%); 0 (0%) vs. 1 (0.8%) 3z. 0 (0%) vs. 1 (0.3%); 0 (0%) vs. 0 (0%) 3zz. 2 (0.5%) vs. 4 (1.2%); 0 (0%) vs. 3 (2.4%)  4. 59/152 (38.8%) vs. 56/126 (44.4%); 1.55 vs. 1.72; 0.89 (0.61 to 1.28) | … | 0 |
| Hicks  2008[26]  USA | **1. Proportion of visits with triggered or suppressed reminders that had adherence to guideline medication prescribing within 1 week; adjusted OR (95% CI). (primary)** | 1. 7% vs. 5%; 1.32 (1.09 to 1.61); P=.002  No interaction for intervention effect by race/ethnicity. | **At 18 months. 1. n/N (%) patients with BP controlled; adjusted OR (95% CI). (primary)**  Prespecified  1. Mean BP at 18 months (mm Hg). 1a. Systolic. 1b. Diastolic. | 1. 410/859 (48%) vs. 527/1168 (45%); 0.96 (0.78 to 1.19); *P=*NS  Secondary analyses excluding patients without documented BP at index or outcome visit was consistent and analysis by race/ethnicity showed no difference in intervention effects (data not reported).  1a. 138 vs. 137, *P*=.67 1b. 77 vs. 78, *P*=.05  Secondary analysis: no difference in intervention effects by race/ethnicity. | + | 0 |
| Matheny  2008[28]  USA | Components of primary 1. Proportion of appropriate laboratory tests within 14 days of the clinical encounter (Medication–lab reminder): number of visits with overdue tests ordered/number of visits with overdue tests, %; adjusted odds ratio (95% CI). **1a. NSAID-Creatinine (8487 vs. 9307 visits).  1b. ARB-Creatinine (751 vs. 832 visits).  1c. Metformin-Creatinine (856 vs. 781 visits) 1d. Potassium supplement – Potassium (579 vs. 751 visits). 1e. Potassium sparing diuretic – Potassium (761 vs. 875 visits). 1f. Thiazide diuretic- Potassium (1997 vs. 2508 visits). 1g. ACE-I – Potassium (2279 vs. 2790 visits). 1h. Statin – ALT (9441 vs. 10935 visits). 1i. Thyroxine – thyroid-stimulating hormone (897 vs. 1233 visits).** | 1a. 150/442, 33.9% vs. 136/428, 31.8%; 1.24 (0.71 to 2.15), *P*=.457 1b. 17/31, 54.8% vs. 17/27, 63.0%; 0.24 (0.04 to 1.34), *P*=.104 1c. 7/20, 35.0% vs. 6/16, 37.5%; 0.53 (0.05 to 5.34), *P*=.594 1d. 7/12, 58.3% vs. 5/9, 55.5%; 0.91 (0.03 to 24.44), *P*=.956 1e. 13/19, 68.4% vs. 17/28, 60.7%; 0.82 (0.12 to 5.60), *P*=.836 1f. 40/62, 64.5% vs. 46/89, 51.7%; 1.30 (0.63 to 2.67), *P*=.473 1g. 57/119, 47.9% vs. 40/80, 50.0%; 1.00 (0.43 to 2.30), *P*=.993 1h. 291/613, 47.5% vs. 358/674, 53.1%; 0.89 (0.43 to 1.81), *P*=.740 1i. 22/38, 57.9% vs. 25/44, 56.8%; 1.19 (0.40 to 3.53), *P*=.747 | ... | ... | 0 | … |
| Reeve  2008[30]  USA | **Primary**  **1 Number of clinical Intervention/Number of patients; intervention rate per patient (95% CI) over 6 weeks of prompt activation plus 10 day follow-up. 1a. Aspirin interventions in diabetic patients.** 1b. Aspirin interventions in diabetic patients in observed/unobserved arms 1c. Aspirin interventions in diabetic patients in 1st 3 weeks (observers present) in observed/unobserved arms. 1d. Aspirin interventions in diabetic patients in 2nd 3 weeks (observers absent) in observed/unobserved arms. 1e. Aspirin interventions in diabetic patients in last 10 days (prompts inactive) in observed/unobserved arms. | 1a. 201/4174 (4.82%) vs. 0/3721 (0%); 2.55 (95% CI 0.85 to 4.24) interventions per 100 diabetic patients  1b. 160/2128 (7.52%) vs. 0%/ 41/2046 (2.00%) vs. 0% 1c.138/NR (12.6%) vs. 0% / 26/NR (2.3%) vs. 0% 1d. 20/NR (1.84%) vs. 0% / 11/NR (1.3%) vs. 0%  1e. 3/NR (% NR) vs. 0% / 4/NR (% NR) vs. 0% | ... | ... | + | … |
| Davis  2007[32]  USA | **Primary 1. Change in proportion of prescriptions consistent with evidence-based recommendations over 18-50 months (difference, 95% CI).**   By study site: Paediatric Care Centre (PCC, University of Washington outpatient teaching clinic) or Skagit Paediatrics (SP. Primary care paediatric clinic) 2. Change in proportion of prescriptions for otitis media consistent with evidence-based recommendations (difference, 95% CI). PCC over 50 months / SP over 18 months 2a. Antibiotic treatment.  2b. Amoxicillin.  2c. Twice daily treatment.  2d. <10 days of antibiotics.  2e. Dosage.  3. Change in proportion of prescriptions for allergic rhinitis consistent with evidence-based recommendations (difference, 95% CI). PCC over 50 months / SP over 18 months 3a. Appropriate treatment choice.  4. Change in proportion of prescriptions for bronchiolitis consistent with evidence-based recommendations at PCC over 50 months (difference, 95% CI). [Insufficient data for SP site] 4a. Albuterol.  5. Change in proportion of prescriptions for sinusitis, pharyngitis, croup, constipation, or urticaria consistent with evidence-based recommendations (difference, 95% CI). PCC over 50 months / SP over 18 months. 5a. Appropriate treatment choice.   Note: Proportional changes were based on individual-prescription-level data; differences were obtained using analyses adjusted for provider clustering and volume of provider visits.  Note: Very limited data were provided for 2 subanalyses: use of a 1-click prescription change option and exploration of provider fatigue over time. | 1. 4% vs. 1% (8%, 1 to 15) 2a. -20% vs. -23% (15%, 2 to 30) / -5% vs. -27% (24%, 8 to 40) 2b. 12% vs. -23% (-2%, -17 to 13) / 3% vs. -7% (12%, -12 to 37) 2c. 20% vs. 36% (-8%, -28 to 11) / 0% vs. 3% (6%, -21 to 32) 2d. 7% vs. 13% (-7%, -21 to 6) / 0% vs. 0% (0%, -0.1 to 0.6) 2e. 7% vs. 15% (9%, -6 to 24) / -10% vs. -3% (-3%, -17 to 11) 3a. 11% vs. 5% (19%, 4 to 35) / 6% vs. -21% (39%, -32 to 110) 4a. 21% vs. 32% (-6%, -18 to 7) 5a. 15% vs. 3% (15%, -1 to 32) / -14% vs. -19% (26%, -41 to 94) | ... | ... | + | … |
| Heidenreich  2007[33]  USA | Primary **1. Number (proportion) of patients with prescriptions for any β -blocker over 9 months; adjusted OR (95% CI).**  Secondary 2. Number (proportion) of patients with prescriptions for specified β –blockers (carvedilol or metoprolol) over 9 months.  Not prespecified 3. Number (proportion) of patients with prescriptions for any β -blocker over 9 months (excluding those on β–blockers at baseline).   Subgroup analyses (not clearly prespecified) 4. Number (proportion) of patients with prescriptions for any β -blocker over 9 months by referral source.  4a. Inpatients.  4b. Outpatients.  4c. Cardiology clinic patients.   5. Interaction of reminder effect with patient history over 9 months.  5a. Prior heart failure.  5b. COPD.  5c. Prior β –blocker use.  5d. LVEF <35%.  6. Trend in reminder effect over time (2001-2005).   Note: Inconsistency in data for COPD. Text, P=.09; figure 3, P=.08 for reminder effect in those without COPD. No author response to query. | 1. 458/621 (74%) vs. 428/650 (66%), *P*=.002; 1.30 (1.04 to 1.63) 2. 261/621 (42%) vs. 238/650 (37%), *P*=.048  3. 163/292 (56%) vs. 144/327 (44%), *P*=.003 4. *P*=.55 for interaction of referral source and reminder effect. 4a. 190/254 (75%) vs. 171/266 (64%), *P*=NR 4b. 268/367 (73%) vs. 257/284 (67%), *P*=NR 4c. 108/145 (74%) vs. 86/111 (77%), *P*=NR 5a. *P*=.07 for reminder effect in those without prior HF. 5b. *P*=.09 (*P*=.08 in figure 3) for reminder effect in those without COPD. 5c. *P*=.32 5d. *P*=.81 6. *P*>.2 | Not prespecified **1. Survival free of heart failure hospitalization at 1y; hazard ratio (95% CI).** | 1. 0.99 (0.83 to 1.18) | + | 0 |
| Martens  2007[34, 46]  The Netherlands | All measured during 12 month intervention period. 1. Appropriate prescribing when no prescribing of a particular drug was advised: % not prescribing [in accordance with recommendation] (95% CI)  1a. no antibiotics for acute sore throat divided by all patients with acute sore throat considered for prescription. 1b. no antibiotics except after 5 days, feneticilline, azitromycin, fenoxymethylpenicilline for acute sore throat divided by all prescriptions for sore throat. 1c. no antibiotics for acute sinusitis divided by all patients with acute sinusitis considered for prescription. 1d. no prescribing indicated, only prescriptions doxycyclin for acute sinusitis divided by all prescriptions for acute sinusitis. 1e. No statins for newly diagnosed patients with diabetes or CVD between 18 and 70 years with cholesterol <3.5mmol divided by all same population considered for prescription.  **2. Appropriate prescribing of antibiotics when no prescribing of a particular drug was advised: volume per GP per 1000 enlisted patients. (95% CI).** 2a. Doxycyclin and amoxicillin for acute bronchitis. 2b. Antibacterial antibiotics (for systemic use) for sore throat. 2c. Feneticilline, azitromycin, fenoxymethylpencilline for acute sore throat. 2d. Antibacterial antibiotics (for systemic use) without doxycyclin for acute sinusitis. 2e. Doxycyclin for acute sinusitis. 2f. Amoxicillin and azitromycin for otitis media acuta. 2g. Antibacterial antibiotics (for systemic use) for otitis media acuta. 2h. Quinolones for cystitis in women >12 years of age. **2i. Sum score for antibiotic prescription (primary).  3. Appropriate prescribing for asthma/COPD when no prescribing of a particular drug was advised: volume per GP per 1000 enlisted patients. (95% CI).** 3a. Prescriptions for intermittent asthma and maintenance treatment. 3b. Inhaled corticosteroids for newly diagnosed COPD in patients >40 years. **3c. Sum score for asthma/COPD prescriptions (primary).  4. Appropriate prescribing of statins for patients with newly diagnosed diabetes mellitus or CVD, 18-70 years of age, and cholesterol <3.5mmol, when no prescribing of a particular drug was advised: volume per GP per 1000 enlisted patients. (95% CI) (primary).**  5. Appropriate prescribing when prescribing of a particular drug was advised: % prescribing [in accordance with recommendation] (95% CI)  5a. benzolyperoxi and salicylacid for acne vulgaris divided by all prescriptions for acne vulgaris. 5b. erythromycin, minocyclin, cyproteronacetate for acne vulgaris divided by all prescriptions for acne vulgaris. 5c. minocyclin, benzoylperoxi, salicyl acid for acne vulgaris (comedones with inflammation, symptoms) divided by all prescriptions for acne. 5d. Fenoxymethyl penicillin, feneticillin, erytromycin for erysipelas divided by all prescriptions for erysipelias. 5e. Fusedine acid, zinc preparation with an desinfectant for impetigo divided by all prescriptions for impetigo. 5f. flucloxacillin, azitromycin for impetigo divided by all prescriptions for antibacterial antibiotics for impetigo. 5g. co-trimoxazol, ciprofloxacin and norfloxacin for chronical and recurrent symptoms on prostatitis divided by all antibacterial antiobiotic prescriptions for same condition. 5h. trimethoprim, nitrofurantoin for acute and recurrent cystitis among female patients >12 years divided by all prescriptions for same population. 5i. Terbutalin turbohaler/salbutamol diskus/salbutamoldosis-aerosol for intermittent/mildly persistent and moderate persistent asthma with acute complaints among patients >7 years divided by all asthma prescriptions for same population. 5j. Budesonide turbuhaler/fluticason discus/fluticasondosis-aerosol for mildly persistent asthma with maintenance treatment among patients >7 years divided by all asthma prescriptions for same population.  5k. Budesonide turbuhaler/fluticason diskus/fluticason dosis-aerosol AND: salmeterol discus/salmeterol dosis-aerosol/formoterol dosis-aerosol for severe persistent asthma with maintenance treatment among patients >7 years divided by all asthma prescriptions for same population. 5l. ipratropiumbromid powder inhaler, ipratropiumbromid dosis-aerosol, salbutamol discus, salbutamol dosis-aerosol for newly diagnosed COPD patients >40 years divided by all prescriptions for COPD patients >40 years of age. 5m. statins for newly diagnosed patients with diabetes or CVD between 18 and 70 years and cholesterol >5.5mmol divided by all statin prescriptions for newly diagnosed diabetes mellitus or CVD.  **6. Appropriate prescribing of particular antibiotics: volume per GP per 1000 enlisted patients. (95% CI).** 6a. benzolyperoxi and salicylacid for acne vulgaris (mainly comedones). 6b. erythromycin, minocyclin, cyproteronacetate for acne vulgaris (mainly inflammation, symptoms). 6c. minocyclin, benzoylperoxi, salicyl acid for acne vulgaris (comedones with inflammation, symptoms). 6d. Fenoxymethyl penicillin, feneticillin, erytromycin for erysipelas. 6e. Fusedine acid, zinc preparation combined with an desinfectant for impetigo. 6f. flucloxacillin, azitromycin for impetigo. 6g. co-trimoxazol, ciprofloxacin and norfloxacin for chronical and recurrent symptoms on prostatitis. 6h. trimethoprim, nitrofurantoin for acute and recurrent cystitis among female patients >12 years. **6i. Sum score for antiobiotic prescriptions (primary).  7. Appropriate prescribing of particular drugs for asthma/COPD treatment: volume per GP per 1000 enlisted patients. (95% CI).** 7a. Terbutalin turbohaler/salbutamol diskus/salbutamol dosis-aerosol for intermittent/mildly persistent and moderate persistent asthma with acute symptoms among patients >7 years. 7b. Budesonide turbuhaler/fluticason discus/fluticason dosis-aerosol for mildly persistent asthma with maintenance treatment among patients >7 years.  7c. Budesonide turbuhaler/fluticason diskus/fluticason dosis-aerosol AND: salmeterol discus/salmeterol dosis-aerosol/formoterol dosis-aerosol for severe persistent asthma with maintenance treatment among patients >7 years. 7d. ipratropiumbromid powder inhaler, ipratropiumbromid dosis-aerosol, salbutamol discus, salbutamol dosis-aerosol for newly diagnosed COPD patients >40 years **7e. Sum score for asthma/COPD drug prescriptions (primary).  8. Appropriate prescribing of particular cholesterol-lowering drugs: volume per GP per 1000 enlisted patients. (95% CI) (primary).**  Note: also reports volume of prescriptions for all antibiotics, % of prescriptions for inhaled corticosteroids in asthma patients, and volume of prescriptions for inhaled corticosteroids in asthma patients; however, only reports data for ‘clinically meaningful’ results. | 1a. 74% (33 to 94) vs. 75% (59 to 90): NS 1b. 66% (23 to 100) vs. 46% (16 to 74): NS 1c. 67% (59 to 73) vs. 61% (51 to 70): NS 1d. 39% (31 to 49) vs. 42% (32 to 58): NS 1e. 100% (0) vs. 98% (94–100): NS  2a. 4.4 (2.8 to 8.6) vs. 5.1 (2.8 to 10.6) 2b. 0.2 (0.0 to 0.6) vs. 0.3 (0.1 to 0.7) 2c. 0.2 (0.0 to 0.4) vs. 0.8 (0.3 to 2.4), *P*=.03 2d. 4.5 (2.9 to 6.4) vs. 6.1 (4.4 to 8.6) 2e. 7.6 (5.0 to 10.4) vs. 10.6 (7.5 to 18.1) 2f. 4.6 (2.5 to 13.7) vs. 5.6 (3.8 to 8.1) 2g. 5.3 (2.9 to 12.5) vs. 6.5 (4.5 to 10.3) 2h. 1.5 (0.8 to 2.2) vs. 4.6 (2.8 to 8.1), *P*=.03 2i. 28.2 (20.8 to 44.5) vs. 39.7 (29.7 to 64.1), NS  3a. 1.1 (0.5 to 2.3) vs. 1.7 (0.8 to 3.3) 3b. 0 (0.0 to 0.1) vs. 0.5 (0.3 to 0.9), *P*=.00 3c. 1.1 (0.6 to 2.6) vs. 2.2 (1.4 o 4.3), NS  4. 0 vs. 0.1 (0.0 to 0.2), NS  5a. 19% (7 to 38) vs. 24% (9 to 49): NS 5b. 59% (42 to 72) vs. 68% (56 to 77): NS 5c. 50% (32 to 73) vs. 35% (17 to 52): NS 5d. 29% (21 to 38) vs. 28% (16 to 37): NS 5e. 64% (49 to 76) vs. 57% (40 to 65): NS 5f. 30% (16 to 42) vs. 26% (14 to 46): NS 5g. 47% (23 to 65) vs. 53% (24 to 81): NS 5h. 73% (69 to 80) vs. 57% (52 to 63); *P*=.01  5i. 47% (38 to 54) vs. 51% (39 to 65): NS 5j. 44% (30 to 56) vs. 27% (14 to 47): NS 5k. 36% (20 to 53) vs. 51% (26 to 78): NS 5l. 15% (9 to 29) vs. 15% (8 to 23): NS 5m. 88% (71 to 100) vs. 72% (52 to 81): NS  6a. 0.3 (0.1 to 1.2) vs. 0.3 (0.1 to 0.5)  6b. 1.9 (1.1 to 2.8) vs. 2.0 (1.3 to 3.1) 6c. 0.6 (0.3 to 1.1) vs. 0.4 (0.1 to 1.1) 6d. 1.1 (0.6 to 2.5) vs. 1.2 (0.6 to 2.2). 6e. 5.0 (3.5 to 8.6) vs. 4.4 (2.6 to 7.0) 6f. 0.7 (0.3 to 1.5) vs. 0.5 (0.2 to 0.8)  6g. 0.8 (0.4 to 1.9) vs. 0.4 (0.2 to 0.9) 6h. 10.1 (7.6 to 14.0) vs. 11.5 (6.9 to 19.3) 6i. 20.7 (17.1 to 26.1) vs. 20.5 (14.2 to 27.4), NS   7a. 3.3 (2.1 to 4.6) vs. 4.8 (3.3 to 6.9)  7b. 1.7 (1.0 to 2.6) vs. 1.4 (0.7 to 4.1) 7c. 0.3 (0.1 to 0.7) vs. 0.5 (0.3 to 1.0) 7d. 0.7 (0.3 to 1.1) vs. 1.0 (0.6 to 1.7)  7e. 5.9 (3.8 to 7.9) vs. 7.7 (5.6 to 11.8), NS  8. 1.0 (0.5 to 2.2) vs. 1.2 (0.7 to 1.8), NS | ... | ... | 0 | … |
| Peterson  2007[35]  USA | **1. median (IQR) ratio of overall prescribed to recommended doses (primary)** 2. median (IQR) ratio of prescribed to recommended doses by type (not prespecified) 2a. antihistamine/anti-emetic 2b. benzodiazepines 2c. neuroleptics 2d. antihypertensives 2e. NSAIDS 2f. antispasmodics 2g. opiates 2h. sulfonylureas 2i. other anticholinergic 2j. other 2k. beers criteria medications 2l. scheduled 2m. PRN 2n. single dose 2o. multiple dose 2p. non-critical care unit 2q. critical care unit and procedure suites 2r. emergency room 2s. subacute unit  3. median (IQR) ratio of overall prescribed to recommended doses by physicians in the intervention group only vs. physicians in the control group only (not prespecified)  4. percentage of recommended doses selected (not prespecified) | 1. 2.5 (1.0,4.0) vs. 3.0 (1.5, 5.0) (*P* <.001) 2a. 4.0 [2.0 , 4.0] vs. 4.0 [2.0 , 6.0] 2b. 2.0 [1.0 , 4.0] vs. 2.5 [1.2 , 4.2] 2c. 4.0 [1.0 , 10] vs. 4.0 [1.0 , 10] 2d. 2.0 [1.0 , 4.0] vs. 2.0 [1.0 , 4.0] 2e. 4.0 [1.5 , 4.0] vs. 4.0 [2.0 , 4.0] 2f. 2.0 [1.0 , 4.0] vs. 3.0 [1.1 , 6.0] 2g. 1.0 [0.5 , 1.5] vs. 1.0 [0.4 , 1.5] 2h. 4.0 [2.0 , 6.5] vs. 4.0 [2.0 , 8.0] 2i. 2.5 [2.0 , 5.0] vs. 2.5 [1.0 , 5.0] 2j. 1.0 [1.0 , 1.6] vs. 1.3 [1.0 , 2.0] 2k. 2.0 [1.0 , 4.0] vs. 2.0 [1.0 , 4.0] 2l. 2.0 [1.0 , 4.0] vs. 2.0 [1.0 , 4.0] 2m. 4.0 [3.0 , 6.0] vs. 4.0 [3.0 , 7.5] 2n. 1.0 [1.0 , 2.0] vs. 1.25 [1.0 , 2.0] 2o. 4.0 [2.0 , 6.0] vs. 4.0 [2.0 , 6.0] 2p. 2.5 [1.0 , 4.0] vs. 3.0 [1.3 , 5.0] 2q. 3.0 [1.5 , 6.0] vs. 3.0 [2.0 , 6.0] 2r. 2.0 [1.0 , 4.0] vs. 2.0 [1.0 , 4.0] 2s. 3.0 [1.5 , 6.0] vs. 4.0 [2.0 , 4.0]  3. 2.0 [1.0,4.0] vs. 4.0[2.0,6.0] (*P* <.001) 4. 28.6% vs. 24.1% (*P* <.001) | ... | ... | + | … |
| Raebel  2007a[37]  USA | 1y study period. Primary outcomes. **1. Rate of all first dispensings of targeted potentially inappropriate medications, n/N (%).  1a. ≥ 1 medication.** 1b. 1 medication. 1c. 2 different medications. 1d. 3 different medications.   2. Rate of dispensings of specific targeted potentially inappropriate medications, n/N (%).  2a. Amitriptyline. 2b.Chlordiazepoxide. 2c. Diazepam. 2d. Doxepin. 2e. Flurazepam. 2f. Ketorolac. 2g. Meperidine (oral). 2h.Oxycodone/aspirin. 2i. Total. 3. Rate of dispensings of specific targeted medications for indications considered inappropriate, n/N (%).  3a. Amitriptyline. 3b.Chlordiazepoxide. 3c. Diazepam. 3d. Doxepin. 3e. Flurazepam. 3f. Ketorolac. 3g. Meperidine (oral). 3h.Oxycodone/aspirin. 3i. Total. | 1a. 543/29840 (1.8%) vs. 644/29840 (2.2%) (*P*=.002). 1b. 535/29840 vs. 632/29840 1c. 8/29840 vs. 11/29840 1d. 0 vs. 1/29840, *P*=.90 for 1b-1d   2a. 114/29840 (0.38%) vs. 183/29840 (0.61%), *P<.*001 2b. 11/29840 (0.04%) vs. 14/29840 (0.05%), *P*=.55 2c. 383/29840 (1.28%) vs. 411/29840 (1.38%), *P*=.32 2d. 32/29840 (0.11%) vs. 42/29840 (0.14%), *P*=.24 2e. 4/29480 (0.01%) vs. 2/29840 (0.01%), *P*=.69 2f. 2/29840 (0.01%) vs. 0 (0%), *P*=.50  2g. 4/29840 (0.01%) vs. 4/29840 (0.01%), *P*=NA  2h. 1/29840 (0%) vs. 1/29840 (0%), *P*=NA 2i. 551/29840 (1.85%) vs. 657/29840 (2.20%), *P*=.002  3a. 111/29840 (0.37%) vs. 175/29840 (0.59%), *P*<.001, RR reduction 37% 3b. 11/29840 (0.04%) vs. 14/29840 (0.05%), *P*=.55 3c. 167/29840 (0.56%) vs. 213/29840 (0.71%), *P*=.02, RR reduction 21%  3d. 27/29840 (0.09%) vs. 38/29840 (0.13%), *P*=.17 3e. 4/29480 (0.01%) vs. 2/29840 (0.01%), *P*=.69 3f. 2/29840 (0.01%) vs. 0 (0%), *P*=.50  3g. 4/29840 (0.01%) vs. 4/29840 (0.01%), *P*=NA  3h. 1/29840 (0%) vs. 1/29840 (0%), *P*=NA 3i. 327/29840 (1.10%) vs. 447/29840 (1.50%), *P<.*001 | ... | ... | + | … |
| Raebel  2007b[36]  USA | 4-mo data collection (stopped early for planned 12-month follow-up).  1. Patients dispensed targeted drugs (primary): n/N (%). 1a. Category D drug. 1b. Category X drug. 1c. Category D and X drugs. **1d. Category D or X drugs.**  2. First dispensings of targeted drugs (secondary). 2a. Number from category D or X/number first dispensings of unique drugs (%). 2b. Number (%) of category D/category X drugs dispensed.   3. Of patients who received a targeted drug, number (%) given:  3a. 1 category D or X drug. 3b. 2 different category D or X drugs. 3c. ≥3 different category D or X drugs.  4. Number (%) of first dispensings of specific category D/category X drugs. 4a. ACE-I.  4b. Antidepressant. 4c. Antineoplastic. 4d. Barbiturate. 4e. Benzodiazepine. 4f. β-blocker. 4g. Clomiphene citrate. 4h. Codeine. 4i. Oestrogens (not oral contraceptives). 4j. Lithium carbonate. 4k. Misoprostol. 4l. Nonsteroidal anti-inflammatory agent. 4m. Narcotic analgesic (not codeine).  4n. Oral contraceptive  4o. Phenytoin. 4p. Propylthiouracil. 4q. Progesterone (not oral contraceptives). 4r. Sulfamethoxazole-trimethoprim. 4s. Tretinoin. 4t. Tetracycline derivatives. 4u. Warfarin. 4v. Total | 1a. 108/6075 (1.8%) vs. 198/5025 (3.9%)  1b. 54/6075 (0.9%) vs. 58/5025 (1.2%) 1c. 15/6075 (0.2%) vs. 20/5025 (0.4%), *P*=.05 for 1a-1c. 1d. 177/6075 (2.9%) vs. 276/5025 (5.5%), *P<.*001  2a. 238/593 (40.2%) vs. 361/848 (42.6%), *P*=.36 2b. 166(69.8%)/72(30.3%) vs. 280 (77.6%)/81(22.4%), *P*=.03 for difference in proportions  3a. 133/177 (75.1%) vs. 211/276 (76.5%) 3b. 31/177 (17.5%) vs. 51/276 (18.4%) 3c. 13/177 (7.3%) vs. 14/276 (5.1%), *P*=.60 over 3a-3c.  4a. 0 vs. 1 (0.2%), *P*>.05 4b. 1 (0.4%) vs. 2 (0.6%), *P*>.05 4c. 0 vs. 3 (0.8%), *P*>.05 4d. 8 (3.4%) vs. 16 (4.4%), *P*>.05 4e. 8 (3.4%) vs. 15 (4.2%), *P*>.05 4f. 4 (1.7%) vs. 8 (2.2%), *P*>.05 4g. 5 (2.1%) vs. 11 (3.1%), *P*>.05 4h. 29 (12.2%) vs. 54 (15.0%), *P*>.05  4i. 6 (2.5%) vs. 6 (1.7%), *P*>.05 4j. 0 vs. 3 (0.8%), *P*>.05 4k. 5 (2.1%) vs. 6 (1.7%), *P*>.05  4l. 22 (9.2%) vs. 36 (10.0%), *P*>.05 4m. 66 (27.7%) vs. 94 (26.0%), *P*>.05 4n. 53 (22.3%) vs. 53 (14.7%), *P*=.02  4o. 0 vs. 1 (0.3%), *P*>.05  4p. 0 vs. 2 (0.6%), *P*>.05 4q. 2 (0.8%) vs. 6 (1.7%), *P*>.05 4r. 9 (3.8%) vs. 28 (7.8%), *P*>.05 4s. 1 (0.4%) vs. 1 (0.3%), *P*>.05 4t. 18 (7.6%) vs. 15 (4.2%), *P*>.05 4u. 1 (0.4%) vs. 0, *P*>.05 4v. 238 (100%) vs. 361 (100%) | ... | ... | + | … |
| Thomson  2007[38]  UK | 1. Mean (95% CI) difference in decision conflict scale score (negative difference represents lower decision conflict in CCDSS group) 1a. pre-clinic **1b. (primary) immediately post-clinic** 1c. 3 month follow-up  2. (secondary) knowledge scale 2a. knowledge of aspirin pre-clinic 2b. knowledge of aspirin post-clinic 2c. knowledge of aspirin 3 month follow-up 2d. knowledge of warfarin pre-clinic 2e. knowledge of warfarin post-clinic 2f. knowledge of warfarin 3 month follow-up  3. (secondary) Degner’s decision-making preference scale  4. (secondary) Number (proportion) of patients who decided to start or continue warfarin (RR, 95% CI) 4a. all patients 4b. patients not already on warfarin 4c. patients already on warfarin  5. Number of consultations with GPs (secondary).  6. Number of hospital appointments (secondary). | 1a. 0.02 (-0.22 to 0.26) 1b. -0.18 (-0.34 to -0.01), *P*=.036 1c. -0.15 (-0.37 to 0.06)   2a. Not significant 2b. Not significant 2c. Not significant 2d. Not significant 2e. Not significant 2f. Not significant  3. No results provided  4.  4a. 39/53, 73.6% vs. 50/56, 81.7% (0.82, 0.68 to 0.99) 4b. 4/16, 25.0% vs. 15/16, 93.8% (0.27, 0.11 to 0.63) 4c. 35/37, 94.6% vs. 35/40, 87.5% (1.08, 0.94 to 1.24)  5. 39 vs. 32  (*P*=.35)  6. 29 vs. 10, *P*=.06 | Secondary; 3-month follow-up  **1. Number of patients admitted to hospital.  2. Adverse events. 2a. Transient ischemic attack**  **2b. Bleed with GP consultation. 2c. Stroke. 2d. Bleed requiring hospital admission.**  **3. (secondary) State Trait Anxiety Inventory – mean change in anxiety from pre-clinic to post-clinic** | 1. 3/53 vs. 4/56  2a. 0/53 vs. 1/56 2b. 0/53 vs. 1/56 2c. 0/53 vs. 0/56  2d. 0/53 vs. 0/56   3. no difference between groups, *P*=.98; -4.57 (95% CI -6.30 to -2.84) for all patients | + | 0 |
| Verstappen  2007[39]  The Netherlands | … | … | **1. number (%) of patients in remission for ≥ 3 months**  1a. in first year **1b. in first two years (primary)** 2. area under the curve (IQR) standardised to time (lower = better outcome for CCDSS) (secondary) 2a. morning stiffness 2b. ESR 2c. tender joint count 2d. swollen joint count 2e. VAS general well-being 2f. VAS pain 2g. functional disability 3. Number (%) of patients meeting modified ACR50 criteria (pre-specified) 3a. at one year 3b. at two years  4. mean (95% CI) time (months) until the first period of remission (not pre-specified) 5. duration (CI) (months) of all periods of remission together (not pre-specified)  6. median (IQR)/mean (95%CI) annual radiographic progression over 2 years (units/year) (not pre-specified)  Adverse events were evaluated at each visit according to a predefined protocol.  7. percentage of patients with AE) 8. number of adverse events/number of protocol visits after methotrexate initiated   9. percentage of total number of adverse events 9a. gastrointestinal 9b. mucocutaneous reaction 9c. neurological disorders 9d. renal events 9e. liver toxicity 9f. haematological abnormalities 9g. pulmonary symptoms 9h. post-dosing reactions of methotrexate 9i. other  10. mean (SD) change from baseline after 1 year (prespecified) CCDSS vs. Control, Mean (95%CI) difference;  10a. ESR, mm/h1st – all patients 10b. ESR, mm/hlst - completers 10c. Morning stiffness, min. - all patients  10d. Morning stiffness, min. - completers  10e. Number of swollen joints – all patients  10f. Number of swollen joints - completers  10g. Number of tender joints - all patients 10h. Number of tender joints - completers  10i. VAS general well-being, mm – all patients 10j. VAS general well-being, mm - completers  10k VAS pain, mm - all patients  10l. VAS pain, mm - completers 10m. Functional disability, Health Assessment Questionnaire - all patients  10n. Functional disability, Health Assessment Questionnaire - completers   11. mean (SD) change from baseline after 2 years (prespecified) CCDSS vs. Control, Mean (95%CI) difference  11a. ESR, mm/h1st – all patients 11b. ESR, mm/hlst - completers 11c. Morning stiffness, min. - all patients  11d. Morning stiffness, min. - completers  11e. Number of swollen joints – all patients  11f. Number of swollen joints - completers  11g. Number of tender joints - all patients 11h. Number of tender joints - completers  11i. VAS general well-being, mm – all patients 11j. VAS general well-being, mm - completers  11k VAS pain, mm - all patients  11l. VAS pain, mm - completers 11m. Functional disability, Health Assessment Questionnaire - all patients  11n. Functional disability, Health Assessment Questionnaire - completers | 1a. 53 (35%) vs. 21 (14%), *P*<.001 1b. 76 (50%) vs. 55 (37%), *P*=.029 2a. 17.0 (7.5 to 41.2) vs. 23.7 (12.3 to 56.7), *P*=.009 2b. 17.7 (10.2 to 27.6) vs. 21.6 (13.0 to 33.6), *P*=.007 2c. 3.6 (1.9 to 6.0) vs. 5.5 (2.8 to 9.2), *P*<.001 2d. 2.7 (1.5 to 5.2) vs. 4.7 (2.8 to 7.6), *P*<.001 2e. 19.0 (11.5 to 35.4) vs. 31.2 (16.2 vs. 44.6), *P*<.001 2f. 12.0 (5.0 to 24.3) vs. 19.0 (9.5 to 34.1), *P*=.001 2g. 0.64 (0.3 to 1.3) vs. 0.80 (0.3 to 1.2), *P*=.8 3a. 87 (58%) vs. 64 (43%), *P*=.018 3b. 69 (46%) vs. 67 (45%), *P*=1.00 4. 10.4 (9.1 to 11.7) vs. 14.3 (12.6 to 16.1), *P*<.001 5. 11.6 (10.1 to 13.1) vs. 9.1 (7.6 to 10.6), *P*=.025 6. 0 (0 to 2.0) / 1.9 (1.0 to 2.7) vs. 0 (0 to 2.5) / 2.1 (1.3 to 2.8), *P*=.9  7. 94% vs. 87%   8. 2378/3190 vs. 873/1132  9a. 24.6% vs. 25.2% 9b. 14.8% vs. 18.2% 9c. 18.8% vs. 18.8% 9d. 2.4% vs. 2.8% 9e. 23.2% vs. 18.6% 9f. 7.1% vs. 4.2% 9g. 2.0% vs. 5.3% 9h. 1.8% vs. 2.1% 9i. 5.2% vs. 4.8%  10a. -18 (27) vs. -15 (24), -3 (-9 to 2) 10b. -24 (27) vs. -16 (24), -7 (-15 to -0.4) 10c. -63 (61) vs. -56 (59), -7 (-21 to 6) 10d. -73 (56) vs. -64 (57), -9 (-25 to 7) 10e. -11 (8) vs. -9 (7), -2 (-4 to -1) 10f. -14 (7) vs. -10 (8), -3 (-5 to -1) 10g. -11 (7) vs. -8 (8), -3 (-6 to -1) 10h. -13 (8) vs. -9 (8), -4 (-6 to -1) 10i. -32 (29) vs. -21 (29), -11 (-17 to -4) 10j. -38 (27) vs. -24 (29), -14 (-22 to -6) 10k. -36 (31) vs. -24 (30), -11 (-18 to -4) 10l. -42 (27) vs. -27 (30), -15 (-23 to -7) 10m. -0.44 (0.59) vs. -0.39 (0.66), -0.05 (-0.19 to 0.09) 10n. -0.56 (0.53) vs. -0.49 (0.67), -0.07 (-0.24 to 0.10)  11a. -16 (27) vs. -16 (24), -0.3 (-6 to 5) 11b. -22 (27) vs. -19 (24), -3 (-10 to 4) 11c. -56 (68) vs. -57 (63), 1 (-13 to 16) 11d. -60 (70) vs. -69 (60), 8 (-10 to 26) 11e. -11 (8) vs. -11 (8), -0.3 (-2; 2) 11f. -13 (7) vs. -13 (7), -0.4 (-2; 2) 11g. -10 (9) vs. -9 (8), -1 (-3 to 1) 11h. -12 (9) vs. -11 (8), -1 (-4 to 1) 11i. -30 (31) vs. -22 (28), -8 (-15 to -1) 11j. -37 (29) vs. -28 (27), -9 (-16 to -1) 11k. -34 (31) vs. -26 (31), -9 (-16 to -1) 11l. -40 (28) vs. -30 (28), -10 (-18 to -2) 11m. -0.41 (0.64) vs. -0.42 (0.76), 0.01 (-0.15 to 0.17) 11n. -0.55 (0.62) vs. -0.54 (0.79), -0.01 (-0.20 to 0.19) | … | + |
| Feldstein  2006a[22, 41]  USA | 3 CCDSS reminder groups: EMR, automated voice message (AVM), and pharmacy team outreach (PTO).   **1. Number (proportion) of patients who completed all baseline laboratory monitoring**  1a. by day 9, immediately before second reminder **1b. by day 25 (primary)**  2. Time to completion of lab tests: hazard ratio (95% CI). (prespecified) 2a. EMR vs. control. 2b. AVM vs. control. 2c. PTO vs. control. Hazard Ratio >1 indicates benefit for treatment group. 3. Number (proportion) of patients with abnormal test results detected (prespecified).  Economic analysis reported in a supplementary article. Costs were determined from trial data and a mix of other sources, including expert opinion (US $) 4. Total cost of interventions per 100 patients. 5. Incremental cost per 100 patients (incremental cases completed); ICER per additional completed case. 5a. EMR.  5b. AVM vs. control. 5c. PTO vs. AVM.  6. Probability of cost-effectiveness for maximum willingness-to-pay level for an additional completed case. 6a. Willingness to pay, $40. 6b. Willingness to pay, $60. 6c. Willingness to pay, $80.   7. Incremental cost per 100 patients (incremental abnormal cases found); ICER per additional abnormal case found. 7a. EMR. 7b. AVM vs. control. 7c. PTO vs. AVM.  8. Probability of cost-effectiveness for maximum willingness-to-pay level for an additional abnormal case found. 8a. Willingness to pay, $400. 8b. Willingness to pay, $600. 8c. Willingness to pay, $800.   9. Sensitivity analysis based on estimates of time for ordering, reviewing, and follow-up of tests. 9a. Low estimates. 9b. High estimates.   10. Sensitivity analysis of cost of contact for patients in EMR group. | EMR vs. AVM vs. PTO vs. Control 1a. 61/196 (31.3%) vs. 117/267 (43.8%) vs. 184/261 (70.5%) vs. 34/237 (14.3%), *P*<.001; *P*<.05 for all differences among arms. 1b. 95/196 (48.5%) vs. 177/267 (66.3%) vs. 214/261 (82.0%) vs. 53/237 (22.4%), *P*<.001; *P*<.05 for all differences among arms.  2a. 2.5 (1.8 to 3.5), *P*<.001 2b. 4.1 (3.0 to 5.6), *P*<.001 2c. 6.7 (4.9 to 9.0), *P*<.001  3. 10/196 (5.1%) vs. 18/267 (6.7%) vs. 22/261 (8.4%) vs. 7/237 (3.0%), *P*=.06  4. $3748 vs. $4159 vs. $5160 vs. $2092  5a. ICER = dominated by mix of AVM and control (mix would be less expensive and more effective than EMR). 5b. $2067 (44); $47  5c. $1001 (16); $64  6a. 0.02 vs. 0.14 vs. 0.00 vs. 0.84 6b. 0.00 vs. 0.59 vs. 0.39 vs. 0.01 6c. 0.00 vs. 0.16 vs. 0.84 vs. 0.00  7a. ICER dominated. 7b. $2067 (3.79); $546 7c. $1001 (1.69); $593  8a. 0.09 vs. 0.18 vs. 0.11 vs. 0.62 8b. 0.13 vs. 0.30 vs. 0.35 vs. 0.22 8c. 0.12 vs. 0.32 vs. 0.50 vs. 0.07  9a. No difference in ICER ranking, AVM ICER = $44 9b. No difference in ICER ranking, AVM ICER = $50  10. Data not reported. States “even if the cost of patient contact were reduced to zero…the EMR arm would never be the optimal strategy.” | ... | ... | + | … |
| Judge  2006[42]  USA | **1. Alerts followed by appropriate prescriber action during the 1 year study period. n/N, %; RR (95% CI) (prespecified).**  2. Alerts, within each category, followed-up by the prescriber during the 1 year study period. n/N, %; RR (95% CI) (prespecified). 2a. Central nervous system side effects. 2b. Constipation side effects.  2c. Related to orders for warfarin. 2d. Potential renal insufficiency or electrolyte imbalance. 2e. Hypokalaemia. 2f. Dose recommendations. 2g. Hyperkalaemia. 2h. Anticholinergic side effects. 2i.Related to orders for multiple antiplatelets. 2j.Drug interactions. 2k.Orders for phenytoin. | 1. 606/1982, 31% vs. 513/1861, 28%; 1.1 (1.00 to 1.2) 2a. 78/447, 17% vs. 53/427, 12%; 1.4 (1.0 to 1.9) 2b. 60/271, 22% vs. 75/307, 24%; 0.91 (0.67 to 1.2) 2c. 61/248, 25% vs. 19/269, 7%; 3.5 (2.1 to 5.7) 2d. 146/288, 51% vs. 133/221, 60%; 0.84 (0.72 to 0.99)  2e. 151/233, 65% vs. 118/178, 66%; 0.98 (0.85 to 1.1) 2f. 20/189, 11% vs. 17/206, 8%; 1.3 (0.69 to 2.4) 2g. 53/140, 38% vs. 59/129, 46%; 0.83 (0.62 to 1.1) 2h. 18/75, 24% vs. 13/53, 25%; 0.98 (0.53 to 1.8) 2i. 7/42, 17% vs. 9/27, 33%; 0.50 (0.21 to 1.2) 2j. 10/42, 24% vs. 4/30, 13%; 1.8 (0.62 to 5.2) 2k. 2/7, 29% vs. 13/14, 93%; 0.31 (0.09 to 1.0) | ... | ... | 0 | … |
| Kattan  2006[43]  USA | Prespecified  **1. Number of weeks from the first scheduled provider visit after symptoms warranting a step-up in therapy to a step-up in medication use by percent of study participants. a. Entire 1 year period, p-value** b. First 6 months hazard ratio, p-value (not pre-specified) **2. Actions within 2 months of medication step-up recommendation.** 2a. % scheduled visits. **2b. % of visits resulting in medication step-up.** | 1a.See figure 2 for graph, faster with CCDSS, *P*=.15 1b. 2.95; *P*= .04  2a. 17.1% vs. 12.3%, *P*=.005 2b. 46.0% vs. 35.6%, *P*=.03 | All reported as mean (SE); p-value **1. Maximum symptom days per 2 weeks (primary)** 2. Days limited in activities for more than half day per 2 weeks (not prespecified) 3. School days missed per 2 weeks (prespecified) 4. Number of ED visits per year (prespecified) 5. Number of unscheduled clinic visits per year (not prespecified) 6. Number of hospitalizations per year (prespecified)  Note: Data available for subgroup of 226 children who needed and received medication step-up. | 1. 3.43 (0.11) vs. 3.52 (0.11); *P=*.54 2. 1.42 (0.07) vs. 1.60 (0.08); *P=*.09 3. 0.67 (0.04) vs. 0.72 (0.04); *P=*.38 4. 0.87 (0.07) vs. 1.14 (0.08); *P=*.013 5. 1.14 (0.08) vs. 1.31 (0.08); *P=*.14 6. 0.22 (0.03) vs. 0.24 (0.03); *P=*.56 | + | 0 |
| Palen  2006[47]  USA | Prespecified **1. Rate of compliance with ordering the recommended laboratory monitoring* for patients prescribed study medications. 1a. Overall, n/N dispensings (%).** 1b. ACE-Is, N dispensings (% compliance). 1c. Allopurinol, N dispensings (% compliance).  1d. Carbamazepine, N dispensings (% compliance). 1e. Colchicine, N dispensings (% compliance). 1f. Digoxin, N dispensings (% compliance). 1g. Diuretic, N dispensings (% compliance). 1h. Gemfibrozil, N dispensings (% compliance). 1i. Isoniazid, N dispensings (% compliance). 1j.Losartan potassium, N dispensings (% compliance). 1k. Metformin hydrocholoride, N dispensings (% compliance). 1l. Methotrexate, N dispensings (% compliance)  1m. Niacin, N dispensings (% compliance). 1n. Phenytoin sodium, N dispensings (% compliance). 1o. Pioglitazone hydrochloride, N dispensings (% compliance). 1p. Potassium chloride, N dispensings (% compliance). 1q. Rifampin, N dispensings (% compliance) 1r. Statins, N dispensings (% compliance). 1s. Valproic acid, N dispensings (% compliance).  Subgroup analysis (not prespecified). 2. Rate of compliance with ordering the recommended laboratory monitoring for patients prescribed study medications, %. 2a. Male patients. 2b. Female patients.  *Compliance = test completed from 180 d before to 14 d after the time of the medication order. | 1a. 10,494/18556 (56.6%) vs. 8957/15686 (57.1%), *P*=.31 1b. 3099 (47.0%) vs. 2729 (47.5%), *P*=.681  1c. 429 (57.6%) vs. 355 (61.1%), *P*=.31  1d. 153 (34.6%) vs. 119 (35.3%), *P*=.91 1e. 411 (52.8%) vs. 400 (46.0%), *P*=.05 1f. 242 (55.0%) vs. 178 (48.9%), *P*=.22 1g. 5384 (44.0%) vs. 4270 (45.6%), *P*=.11 1h. 569 (71.2%) vs. 454 (62.3%), *P*=.003 1i. 33 (15.2%) vs. 36 (19.4%), *P*=.64 1j. 506 (52.0%) vs. 433 (52.7%), *P*=.84 1k. 1098 (67.6%) vs. 940 (7.6%), *P*=.14  1l. 7 (42.9%) vs. 9 (0.0%), *P*=.03  1m. 34 (67.7%) vs. 36 (47.2%), *P*=.084 1n. 83 (32.5%) vs. 52 (25.0%), *P*=.35  1o. 76 (92.1%) vs. 63 (93.7%), *P*=.73 1p. 1623 (54.3%) vs. 1291 (57.8%), *P*=.06 1q. 7 (14.3%) vs. 6 (50.0%), *P*=.20 1r. 4717 (75.7%) vs. 4245 (73.9%), *P*=.05 1s. 85 (36.5%) vs. 70 (38.6%), *P*=.79  2a. 57.5% vs. 58.5%, *P*=.18 2b. 55.7% vs. 55.9%, *P*=.82 | ... | ... | 0 | … |
| Paul  2006[48]  Italy, Germany and Israel | **1. Rate of appropriate antibiotic treatment, intervention intention-to-treat OR (95% CI) p value; (n/N(%)) (primary**  **outcome):** 1a. Israel 1b. Germany 1c. Italy **1d. Overall**  2. Rate of appropriate antibiotic treatment, intervention per protocol, OR (95% CI) p value per site (n/N(%)) (primary outcome): 2a. Israel 2b. Germany 2c. Italy 2d. Overall  3. Number (%) of antibiotics prescribed in Israel / Germany / Italy ): (secondary outcome) 3a. no antibiotic 3b. narrow-spectrum penicillins 3c. piperacillin/tazobactam or sulbactam 3d. first-generation cephalosporin 3e. broad-spectrum cephalosporins 3f. Flouroquinolones 3g. aminoglycosides 3h. glycopeptides 3i. carbapanems | 1a. 140/203 (69.0%) vs. 131/206 (63.6%), 1.27 (0.84 to 1.92) *P*=.251 1b. 38/44 (86.4%) vs. 32/43 (74.4%), 2.18 (0.72 to 6.54) *P*=.160 1c. 38/50 (76.0%) vs. 13/4 (54.2%), 2.68 (0.95 to 7.52) *P*=.057 1d. 216/297 (72.7%) vs. 176/273 (64.5%), 1.48 (1.03 to 2.11) *P*=.033  2a. 74/87 (85.1%) vs. 131/206 (63.6%), 3.26(1.69 to 6.27) *P*≤0.001 2b. 18/19 (94.7%) vs. 32/43 (74.4%), 6.19 (0.74 to 51.91) *P*=.062 2c. 22/28 (78.6%) vs. 13/4 (54.2%), 3.10 (0.93 to 10.39), *P*=.061 2d. 114/134 (85.1%) vs. 176/273 (64.5%), 3.42 (1.97 to 5.96), *P*=.001  3a. 173(20%) vs. 172 (21%) / 4(2%) vs. 3(2%) / 28(16%) vs. 8(9%) 3b. 92(11%) vs. 85(10%) / 36(17%) vs. 26(15%) / 44(25%) vs. 8(9%) 3c. 26(3%) vs. 17(2%) / 14(7%) vs. 13(8%) / 11(6%) vs. 3(3%) 3d. 29(3%) vs. 11(1%) / 0 vs. 0 / 0 vs. 0 3e. 333(39%) vs. 405(49%) / 108(52%) vs. 84(49%) / 23(18%) vs. 37(43%) 3f. 144(17%) vs. 98(12%) / 29(14%) vs. 29(17%) / 68(38%) vs. 28(32%) 3g. 33(4%) vs. 15(2%) / 6(3%) vs. 8(5%) / 3(2%) vs. 1(1%) 3h. 26(3%) vs. 21(3%) / 9(4%) vs. 8(5%) / 5(3%) vs. 6(7%) 3i. 5(0.6%) vs. 3(0.4%) / 9(4%) vs. 6(3%) / 6(3%) vs. 3(3%) | 1. Mean/median (SD) duration of hospital stay (prespecified) 1a. Israel 1b. German 1c. Italy **1d. Overall** 2. Mean/median (SD) duration of hospital stay among patients surviving 30 days (N=1837) 2a. Israel 2b. German 2c. Italy 2d. Overall 3. Mean/median (SD) duration of fever (prespecified). 3a. Israel 3b. German 3c. Italy **3d. Overall** 4. Overall 30 day mortality intention to treat, n/N(%) (prespecified) 4a. Israel 4b. German 4c. Italy **4d. Overall** 5. Overall 30 day mortality per protocol, n/N(%) 5a. Israel 5b. German 5c. Italy 5d. Overall | 1a. 4/7.21(9.7) vs. 5/8.04(11.1), *P*=.014 1b. 10/13.6(11.2) vs. 14/16.3(12.0), *P*=.016 1c. 8/12.13(15.7) vs. 7/11.3(10.7), *P*=.600 1d. 6/8.83(11.29) vs. 6/9.45(11.52), *P*= 0.055 2a. 4/7.1(10.2) vs. 5/7.9(11.6), *P*=.032 2b. 11/16.4(13.2) vs. 16/19.9(13.8), *P*=.040 2c. 8/12.2(15.9) vs. 7/11.4(10.7), *P*=.586 2d. 5/8.8(11.9) vs. 5/9.4(12.2), *P*=.128 3a. 1/2.2(4.1) vs. 1/2.5(4.7), *P*=.014 3b. 1/1.9 (2.7) vs. 1/2.1(3.0), *P*=.487 3c. 3/4.0(3.4) vs. 3/3.8(4.3), *P*=.024 3d. 1/2.4(3.9) vs. 1/2.5(4.5), *P*=.253 4a. 113/860(13.1) vs. 128/823(15.6), *P*=.158 4b. 26/208(12.5) vs. 16/172(9.3), *P*=.322 4c. 10/177(5.6) vs. 1/86(1.2), *P*=.109 4d. 149/1153(12.9) vs. 145/1012(14.3), *P*=.611 5a. 35/344(10.2) vs. 38/301(12.6), *P*=.327 5b. 9/69(13.0) vs. 6/53(11.3), *P*=.774 5c. 5/120(4.2) vs. 0/42(0), *P*=.328 5d. 49/503(9.7) vs. 44/371(11.9), *P*=.719 | + | 0 |
| Derose  2005[50]  USA | 1-4 primary outcomes 1. Rate of dispensed prescriptions for ACE-Is or ARBs within 2 weeks after the 1st visit by an eligible patient: n/N (%, 95% CI), p-value.  2. Rate of dispensed prescriptions for statins within 2 weeks after the 1st visit by an eligible patient: n/N (%, 95% CI), p-value.  3. Rate of dispensed prescriptions for either type of medication (ACE-I/ARB or statin) within 2 weeks after the 1st visit by an eligible patient: n/N (%, 95% CI); OR, 95% CI, p-value.  **4. Odds ratio (95% CI) for prescribing ACE-I, ARB, or statins in intervention vs. control group, controlling for number of visits, medication recommended, and patient age, sex, and past medication use.**  Subgroup analyses (not clearly prespecified).  5. Odds ratio for intervention vs. control specialists/primary care physicians.  6. Interaction for number of visits (1 vs. >1) and treatment group (CCDSS vs. control).  Note: Included pts were those eligible for ACE-I/ARB but not dispensed drug in past 12 mo or eligible for statins or other lipid-lowering drug but not dispensed drug in past 6 mo. | 1. 164/2311 (7.1%, 6.1 to 8.2) vs. 134/2367 (5.7%, 4.8 to 6.7), *P*= 0.048 2. 171/2103 (8.1%, 7.6 to 10.2) vs. 160/2080 (7.7%, 6.6 to 8.9), *P*= 0.61 3. NR/4414 (7.6%, 6.8 to 8.4) vs. NR/4447 (6.6%, 5.9 to 7.4), *P*=.08 4. 1.192 (1.01 to 1.40), *P*=.04  5. 1.16/1.20, *P*=.92 for interaction. 6. No significant interaction for # visits and treatment group. | ... | ... | + | … |
| Heidenreich  2005[51]  USA | Primary  **1. Proportion prescribed ≥ moderate daily dose of ACE-I or appropriate alternative at 6 months (including patients on target doses at baseline).**  2. Number (proportion) of patients prescribed ≥ moderate daily dose of ACE-I or appropriate alternative at 6 months (excluding randomized patients who were on ≥ moderate daily doses at baseline); adjusted OR (95% CI).  Not prespecified  3. Proportion prescribed any dose of ACE-I or appropriate alternative at 6 months. | **1. 125/221 (57%) vs. 114/235 (49%), *P*=.09** 2. 52/137 (38%) vs. 37/140 (26%), *P*=.04; 1.70 (1.02 to 2.86), *P*<.05 3. 121/137 (88%) vs. 122/140 (87%), *P*=.77 | Secondary  **1. Mortality for n=251 with follow-up at 1 y; hazard ratio (95% CI).  2. Renal function at 6 months. 2a. Mean (SD) creatinine (mg/dL) for n=258 at 6 months.**  2b. Number (proportion) of patients with creatinine >3 (mg/dL) at 6 months.  **3. Mean (SD) systolic BP (mm Hg) at 6 months.  4. Mean (SD) diastolic BP (mm Hg) at 6 months.** | 1. 0.98 (0.78 to 1.23)  2a. 1.8 (1.8) vs. 1.8 (1.9), *P*>.2 2b. 15/124 (12%) vs. 16/134 (12%), *P*>.2 3. 126 (22) vs. 126 (23), *P*>.2 4. 68 (14) vs. 68 (14), *P*>.2 | 0 | 0 |
| Raebel, 2005[54], USA | **1. Percentage (95% CI) of drug dispensings with baseline laboratory monitoring (from 180 days prior to dispensing until 14 days after) (primary outcome)**  2. n/N, percentage (95% CI) of drug dispensings with baseline laboratory monitoring (from 180 days prior to dispensing until 14 days after) for each drug; difference(comparison by drug not pre-specified) 2a. allopurinol 2b. amiodarone 2c. azathioprine 2d. carbamazepine 2e. divalproex sodium 2f. isotretinoin 2g. lithium 2h. metformin 2i. methotrexate 2j. nefazodone hydrochloride 2k. pioglitazone hydrochloride 2l. statin + gemfibrozil  Note: The number of patients started on felbamate (0 vs. 2) or ticlopidine (5 vs. 7) during the study was low and data on lab monitoring were not presented.  3. percentage (95% CI) of drug dispensings with baseline laboratory monitoring (from 180 days prior to dispensing until 14 days after) broken down by age subgroup (18-39y, 40-49y, 50-59y, 60-69y, 70-79y, ≥80y) (not pre-specified)  **there are other descriptions of findings in intervention group, but these do not compare CDSS vs. control | 1. 79.1% (78.0 to 80.2) vs. 70.2% (68.9 to 71.5), *P*<.001  2a 575/701, 82.0% (79.9 to 84.8) vs. 484/692, 69.9% (66.4 to 73.3); 12.1%; *P*<.001 2b. 202/257, 78.6% (73.1 to 83.5) vs. 107/208, 51.4% (44.4 to 58.4); 27.2%; *P*<.001 2c. 97/108, 89.8% (82.5 to 94.8) vs. 94/112, 83.9% (75.8 to 90.2); 5.9% *P*=.20 2d. 356/499, 71.3% (67.2 to 75.3) vs. 273/484, 56.4% (51.6 to 60.7); 15.9%; *P*<.001 2e. 343/517, 66.3% (62.1 to 70.4) vs. 306/514, 59.5% (55.1 to 63.8); 6.8%; *P*=.02 2f. 105/117, 89.7% (82.8 to 94.6) vs. 141/148, 95.3% (90.5 to 98.1); 5.6%; *P*=.83 2g. 152/285, 53.3% (47.6 to 59.2) vs. 117/272, 43.0% (37.1 to 49.1); 10.3%; *P*=.02 2h. 1538/1855, 82.9% (81.1 to -84.5) vs. 1333/1759, 75.8% (73.7 to 77.8); 7.1%; *P*<.001 2i. 235/259, 90.7% (86.5 to 94.0) vs. 218/246, 88.6% (84.0 to 92.3); 2.1%; *P*=.43 2j. 54/93, 58.1% (47.4 to 68.2) vs. 54/112, 48.2% (38.7 to 57.9); 9.9%; *P*=.16 2k. 122/131, 93.1% (87.4 to 96.8) vs. 103/115, 89.6% (82.5 to 94.5); 3.5%; *P*=.32 2l. 295/326, 90.5% (86.8 to 93.4) vs. 288/345, 83.5% (79.1 to 87.2); 7.0%; *P*=.01  3. values not provided but *P*<.001 in favour of CCDSS | ... | ... | + | … |
| Krall  2004[58]  USA | (no prespecified outcomes) 1. Number (proportion) of patients who were eligible for aspirin therapy at beginning of study who were no longer eligible after 1 month (i.e., practitioner had responded to alert in intervention group or acted similarly in control group). **1a. All patients.** 1b. Patients of physicians and osteopaths.  1c. Patients of nurse practitioners and physician assistants. | 1a. 315/580 (54.3%) vs. 128/496 (25.8%), *P*<.001 1b. 304/554 (54.9%) vs. 113/416 (27.2%), *P*<.001 1c. 11/26 (42.3%) vs. 15/80 (18.8%), *P*=.02 | … | … | + | … |
| Ansari  2003[61]  USA | Primary outcomes. **1. Proportion of patients who were initiated or uptitrated and maintained on β-blockers at 1 y, n/N (%)**   2. Proportion of β-blocker-naive patients who were initiated on β-blockers at 1 y, n/N (%)  **3. Proportion of patients on target β-blocker doses at 1 y, n/N (%).**  Prespecified. 4. Mean time from initiation to achievement of target dose of β-blockers (for patients who reached the target dose.   Note: target doses were carvedilol 50 mg, metoprolol tartrate 100mg, or atenolol 100 mg. | CCDSS vs. Provider Education only vs. Nurse Facilitator (NF)  1.10/64 (16%) vs. 14/51 (27%) vs. 36/54 (67%), *P*<.001 for NF vs. other 2 groups; NS for CCDSS vs. provider education.  2. 5/41 (12%) vs. 10/35 (29%) vs. 22/36 (61%), *P*<.001 for NF vs. other 2 groups; NS for CCDSS vs. provider education.  3. 1/64 (2%) vs. 5/51 (10%) vs. 23/54 (43%), P<.001 for NF vs. other 2 groups; *P*=NR for CCDSS vs. provider education.  [CCDSS vs. Education, *P*=.048 uncorrected chi-square, P=.12 Yates-corrected chi-square, calculated by RA^b^]  4. 9.3 mo vs. 5.9 mo vs. 8.5 mo, P<.001 for NF vs. other 2 groups. | 1 y follow-up. **Prespecified. 1. Number of patients hospitalised or with ED visits, n/N (%).**   2. Number of patients hospitalised for chronic heart failure, n/N (%).   **3. Median hospitalization or ER visits per patient, n.   4. Deaths, n/N (%).** | CCDSS vs. Provider Education only vs. Nurse Facilitator  1. 29/64 (45%) vs. 25/51 (49%) vs. 23/54 (43%), *P*=.81  2. 9/64 (14%) vs. 5/51 (10%) vs. 5/54 (9%), *P*=.66  3. 1/64 (2%) vs. 1/51 (2%) vs. 2/54 (4%), *P*=.14  4. 1/64 (2%) vs. 7/51 (14%) vs. 5/54 (9%), *P*=.05 | 0 | 0 |
| Filippi  2003[62]  Italy | 1. n (%) patients with antiplatelet drug prescription: baseline (12 mo pre-study/follow-up (over 7 mo study); difference (%); OR (95% CI). 1a. Patients with 1 cardiac risk factor and without CVD. (N=2,651 vs. 2,578) 1b. Patients with ≥ 2 cardiac risk factors and without CVD. (N=1,577 vs. 1,440) 1c. Patients with CVD. (N=3,802 vs. 3,295) **1d. All patients (primary). (N=8,030 vs. 7,313)** | 1a. 358 (13.5%)/736 (27.8%) vs. 263 (10.2%)/440 (17.1%); 378 (14.3%) vs. 177 (6.9%); 2.38 (1.97 to 2.87)  1b. 224 (14.2%)/508 (32.2%) vs. 180 (12.5%)/276 (19.2%); 284 (18.0%) vs. 9.6 (6.7%); 3.22 (2.52 to 4.12)  1c. 1,304 (34.3%)/1,768 (46.5%) vs. 1,229 (37.3%)/1,526 (46.3%); 464 (12.2%) vs. 297 (9.0%); 1.36 (1.16 to 1.59)  1d. 1,886 (23.5%)/3,012 (37.5%) vs. 1,672 (22.9%)/2,242 (30.7%); 1,126 (14.0%)* vs. 570 (7.8%)*; 1.99 (1.79 to 2.22); * = *P*<.001 for change from baseline. | ... | ... | + | … |
| Tamblyn  2003[65]  Canada | Primary outcomes (initiation and discontinuation rates) over 13-mo study. **1. Number of inappropriate prescriptions started per 1000 visits; Number (%) of patients given an inappropriate prescription; RR (95% CI).**     2. Number of pre-existing inappropriate prescriptions discontinued per 1000 visits; **Number (%) of patients with pre-existing inappropriate prescriptions discontinued; RR (95% CI).  2a. Any prescriptions.** 2b. All prescriptions.  Secondary outcomes over 13-mo study. 3. Number of inappropriate prescriptions started per 1000 visits by type of prescribing problem; RR (95% CI). 3a. Drug–disease contraindication. 3b. Drug–age contraindication. 3c. Excessive duration of therapy. 3d. Therapeutic duplication. 3e. Drug interaction.  4. Number of patients starting an inappropriate prescription by type of prescribing problem, n/N, %.  4a. Drug–disease contraindication. 4b. Drug–age contraindication. 4c. Excessive duration of therapy. 4d. Therapeutic duplication. 4e. Drug interaction. 5. Number of pre-existing inappropriate prescriptions discontinued per 1000 visits, by type of prescribing problem; RR (95% CI). 5a. Drug–disease contraindication. 5b. Drug–age contraindication. 5c. Excessive duration of therapy. 5d. Therapeutic duplication 5e. Drug interaction.  6. Number of patients with pre-existing inappropriate prescriptions discontinued, by type of prescribing problem, n/N, %. 6a. Drug–disease contraindication. 6b. Drug–age contraindication. 6c. Excessive duration of therapy. 6d. Therapeutic duplication. 6e. Drug interaction. 7. Inappropriate prescriptions discontinued for excessive duration of therapy, by source of prescription. 7a. Total number of pre-existing inappropriate prescriptions. 7b. Study physician as prescriber: % prescriptions, number of discontinuations per 1000 visits; RR (95% CI). 7c. Study physician + another physician as prescribers: % prescriptions, number of discontinuations per 1000 visits; RR (95% CI). 7d. Another physician as prescriber: % prescriptions, number of discontinuations per 1000 visits; RR (95% CI).  8. Inappropriate prescriptions discontinued for therapeutic duplication, by source of prescription. 8a. Total number of pre-existing inappropriate prescriptions. 8b. Study physician as prescriber: % prescriptions, number of discontinuations per 1000 visits; RR (95% CI). 8c. Study physician + another physician as prescribers: % prescriptions, number of discontinuations per 1000 visits; RR (95% CI). 8d. Another physician as prescriber: % prescriptions, number of discontinuations per 1000 visits; RR (95% CI).  9. Inappropriate prescriptions discontinued for drug interaction, by source of prescription. 9a. Total number of pre-existing inappropriate prescriptions. 9b. Study physician as prescriber: % prescriptions, number of discontinuations per 1000 visits; RR (95% CI). 9c. Study physician + another physician as prescribers: % prescriptions, number of discontinuations per 1000 visits; RR (95% CI). 9d. Another physician as prescriber: % prescriptions, number of discontinuations per 1000 visits; RR (95% CI).  Unspecified subgroup analyses. 10. Rate of inappropriate prescriptions: CCDSS vs. control group; RR (95% CI). 10a. Experienced computer users (those who had used computers for recreational or work-related activities). 10b. Inexperienced computer users. 11. Rate of discontinuation of inappropriate prescriptions: RR (CCDSS vs. control) for experienced and inexperienced users. | 1. 43.8 vs. 52.2; 755/4767, 15.8% vs. 909/4603, 19.7%; 0.82 (0.69 to 0.98)  2a. 71.4 vs. 67.4; 1002/1578, 63.5% vs. 1045/1670, 62.6%, 1.06 (0.89 to 1.26) 2b. 35.5 vs. 32.1; 47.5% vs. 44.5%, 1.14 (0.98 to 1.33)  . 3a. 16.6 vs. 18.4; 0.89 (0.72 to 1.10) 3b. 10.7 vs. 13.7; 0.77 (0.59 to 1.00) 3c. 13.3 vs. 17.1; 0.78 (0.61 to 0.99) 3d. 6.1 vs. 6.8; 0.87 (0.69 to 1.11) 3e. 1.6 vs. 1.5; 1.12 (0.68 to 1.87)  4a. 396/5520, 7.2% vs. 470/5469, 8.6%  4b. 283/5727, 4.9% vs. 375/5516, 6.8%  4c. 361/5791, 6.2% vs. 499/5768, 8.7%  4d. 179/6193, 2.9% vs. 217/6188, 3.5%  4e. 49/6221, 0.79% vs. 51/6212, 0.82%   5a. 62.6 vs. 57.9; 1.08 (0.85 to 1.36)  5b. 40.7 vs. 42.9; 0.94 (0.79 to 1.13) 5c. 32.3 vs. 32.6; 1.00 (0.77 to 1.29) 5d. 317.1 vs. 334.0; 0.94 (0.59 to 1.51)  5e. 68.6 vs. 51.5; 1.33 (0.90 to 1.95)  6a. 552/933, 59.2% vs. 522/881, 59.3% 6b. 330/636, 51.9% vs. 401/812, 49.4% 6c. 196/506, 38.7% vs. 208/548, 40.0% 6d. 146/150, 97.3% vs. 170/176, 96.6% 6e. 106/148, 71.6% vs. 89/134, 66.4%  7a. 506 vs. 548 7b. 63.6%, 63.7 vs. 65.5%, 59.7; 1.06 (0.8 to 1.5) 7c. 13.4%, 16.3 vs. 13.0%, 11.4; 1.43 (0.7 to 3.1) 7d. 22.9%, 46.4 vs. 21.5%, 42.3; 1.09 (0.63 to 1.89)   8a. 148 vs. 174 8b. 21.6%, 388.1 vs. 17.8%, 495.7; 0.78 (0.3 to 2.2) 8c. 35.8%, 519.6 vs. 40.2%, 312.1; 1.66 (0.99 to 2.79) 8d. 42.5%, 662.5 vs. 42.0%, 585.6; 1.10 (0.65 to 1.85)  9a. 148 vs. 133 9b. 29.7%, 165.1 vs. 35.3%, 76.5; 2.15 (0.98 to 4.70)  9c. 36.5%, 74.6 vs. 36.8%, 56.1; 1.33 (0.74 to 2.54) 9d. 33.8%, 81.8 vs. 27.8%, 122.0; 0.75 (0.35 to 1.59)  10a. 0.70 (0.55 to 0.89) 10b. 1.03 (0.82 to 1.29)  11. 1.17 vs. 0.93, *P*=.32 for study group/computer experience interaction.   Note: Non-CCDSS factors affecting prescribing included increased copayments for prescriptions when study started, and frequent hardware and software problems early in study (affecting 22% of physicians). | ... | ... | + | … |
| Weir  2003[67]  UK & Germany | **1a. (secondary) Number (%) of “optimal” treatments (the treatment that would provide the lowest estimated event rates according to CCDSS). 1b. (secondary) Median (IQR) rank of therapy prescribed.**  1c. Odds ratio for optimal therapy being prescribed (95% CI) in multilevel model | 1a. 56 (30%) vs. 140 (34%), *P=*NS 1b. 2 (1 to 3) vs. 2 (1 to 3)  1c. 1.32 (0.83 to 1.80) | **(primary)**  **1a. Median (IQR) estimated RR reduction in ischemic and haemorrhagic vascular events that is achieved by actual prescribed therapy vs. “the option of ‘no antiplatelet or anticoagulant therapy’”, estimated from baseline data.** | 1a. 16.7 (13.5 to 22.9) vs. 16.3 (13.1 to 23.8), *P=*NS | 0 | 0 |
| Zanetti[68]  2003  USA | **1. Number (proportion) of patients given an intraoperative redose of antibiotics, n (%); adjusted OR (95% CI). (primary outcome)** | 1. 93/137 (68%) vs. 55/136 (40%); 3.31 (1.97 to 5.61), *P*< .0001.  Note: 227 vs. 222 randomized; 168 vs. 163 could have reminders activated (i.e. surgery documented as >225 mins and patient given antibiotics); and 137 vs. 136 were documented as eligible for intraoperative redosing according to guidelines and were included in primary analysis. | **1. Number (proportion) with surgical-site infection. (secondary outcome)** | 1. 5/137 (4%) vs. 8 /136 (6%); *P*= .4. | + | 0 |
| Christakis  2001[73]  USA | Primary **1. Mean (SE) change in proportion of time antibiotics prescribed for <10 days over 8 months.**  Secondary 2. Mean (SE) change in frequency of no antibiotic prescribing for otitis media over 8 months.  Note: some of this data is also included in Davis, 2007 | 1. 44.43% (4.24) vs. 10.48% (5.25), *P*<.01 2. -4.33% (5.15) vs. -16.81% (5.09), *P*=.095 | ... | ... | + | … |
| Rossi  1997[81]  USA | Main outcome for 6-month study. **1. Prescription changes from a calcium channel blocker to another antihypertensive agent: n/N of patients (%). 1a. Overall.** 1b. Changed to β-blockers. 1c. Changed to diuretics. 1d. Increased ACE-I dose. 1e. Changed to both β-blockers and diuretics. 1f. No other medication substituted. | 1a. 39/346 (11.3%) vs. 1/373 (<1%), *P*<.0001 1b. 26/346 vs. 1/373 1c. 7/346 vs. 0/373 1d. 3/346 vs. 0/373 1e. 2/346 vs. 0/373 1f. 1/346 vs. 0/373 | … | … | + | … |
| Rotman  1996[83]  USA | 1 y study period. **Prespecified 2. Rate of clinically relevant drug interactions.** | 2. No difference   Note: CCDSS was used to write only 2.8% of prescriptions (75 of 2570). | … | … | 0 | … |
| McDonald  1980[87]  USA | Prespecified 1. Mean provider response rate for reminders over 5 weeks. **With references (R1)** vs. without references (R2) **vs. no reminders (C).**   Specific reminders not prespecified for analysis. All data R1/R2 vs. C  2. Number of events detected / mean adherence response rate for reminders by 17 residents over 5 weeks. 2a. Overall.  2b. Record a finding.  2c Order a test.  2d. Change a treatment.  2e. Miscellaneous.   3. Number of events detected / Mean adherence response rate for reminders by 9 interns over 5 weeks. 3a. Overall.  3b. Record a finding.  3c Order a test.  3d. Change a treatment.  3e. Miscellaneous.   4. Number of events detected / mean adherence response rate for reminders over 5 weeks by nurse clinicians. 4a. Overall.  4b. Record a finding.  4c Order a test.  4d. Change a treatment.  4e. Miscellaneous. | 1. 40.9% vs. 35.9% (*P*=.154 for R1 vs. R2) vs. 19.8% (*P*<.001 vs. R1 /R2 combined [38.4%])   2a. 1503, 40% vs. 758, 20%, *P*<.001 2b. 420, 23% vs. 200, 13%, *P*<.015 2c. 725, 49% vs. 374, 20%, *P*<.001 2d. 201, 43% vs. 114, 29%, *P*<.037 2e. 129, 36% vs. 70, 20%, *P*<.058 3a. 422, 41% vs. 204, 17%, *P*<.006 3b. 101, 29% vs. 49, 15%, *P=*NS 3c. 226, 38% vs. 108, 9%, *P*<.017 3d. 45, 62% vs. 21, 28%, *P*<.008 3e. 19, 0% vs. 16, 0% 4a. 608, 30% vs. 196, 25%, *P=*NS 4b. 166, 36% vs. 64, 31%, *P=*NS  4c. 289, 24% vs. 89, 15%, *P=*NS 4d. 104, 37% vs. 28, 29%, *P=*NS 4e. 44, 32% vs. 15, 22%, *P*<.058 | ... | ... | + | … |
| Coe  1977[88]  USA | ... | ... | BP measures were prespecified; other measures were not clearly prespecified.   **1a. Number of patients that achieved adequate BP control (DBP <95 mmHg during treatment).** 1b. Number of patients that achieved incomplete but substantial BP control (DBP 95-105 mmHg during treatment). 1c. Number of patients that did not achieve BP control (DBP >105 mmHg during treatment).  2. Mean (SEM) BP measurements. 2a. SBP/DBP mmHg overall: pretreatment; reduction after treatment.  2b. Mean (SEM) SBP pretreatment/posttreatment in patients with DBP <95 mmHg during treatment. 2c. Mean (SEM) DBP pretreatment/posttreatment in patients with DBP <95 mmHg during treatment. 2d. Mean (SEM) SBP pretreatment/posttreatment in patients with DBP 95 to 105 mmHg during treatment. 2e. Mean (SEM) DBP pretreatment/posttreatment in patients with DBP 95 to 105 mmHg during treatment. 2f. Mean (SEM) SBP pretreatment/posttreatment in patients with DBP >105 mmHg during treatment. 2g. Mean (SEM) DBP pretreatment/posttreatment in patients with DBP >105 mmHg during treatment.  3. Time in compliance, %. 3a. For patients with DBP <95 mmHg during treatment. 3b. For patients with DBP 95 to 105 mmHg during treatment. 3c. For patients with DBP >105 mmHg during treatment.   4. Weeks of treatment, ?mean (SEM). 4a. For patients with DBP <95 mmHg during treatment. 4b. For patients with DBP 95 to 105 mmHg during treatment. 4c. For patients with DBP >105 mmHg during treatment.  5. Weeks of compliance, ?mean (SEM). 5a. For patients with DBP <95 mmHg during treatment. 5b. For patients with DBP 95 to 105 mmHg during treatment. 5c. For patients with DBP >105 mmHg during treatment.  6. Number of patients with side effects from different anti-hypertensive drugs. I.Thiazide (n=NR) a. Gout II.Alphamethyldopa (n=26 vs. 21) a. Somnolence b. Syncope c. Depression d. Reaction e. Cannot take f. No higher dose III. Guanethidine (n=19 vs. 9) a. Postural dizziness b. Syncope c. Impotence d. Diarrhoea e. Reaction  Note: Type of drugs prescribed in each group also reported by final DBP control (Table 3 in article). | 1a. 23/56 vs. 30/60 1b. 17/56 vs. 20/60 1c. 16/56 vs. 10/60  Authors report “blood pressure…response was similar for both groups, as were drug side effects and overt non-compliance with treatment.”  2a. 172(3)/113(2) vs. 167(4)/111(2); 19.5(2.5)/13.4(1.4) vs. 18.3(3.3)/14.5(1.4) Note: *P*<.02 for difference in CCDSS and control regression slopes for SBP; no difference reported for DBP.  2b. 165(4)/142(3) vs. 162(5)/136(3) 2c. 105(2)/90(0.9) vs. 107(2)/89(0.9) 2d. 167(5)/151(6) vs. 163(7) /154(4) 2e. 110(2)/100(0.7) vs. 108(2)/98(0.6)  2f. 187(5)/168(5) vs. 189(11)/173(7)  2g. 129(3)/112(2) vs. 129(4)/116(3)   3a. 74.2% vs. 79.6% 3b. 72.8% vs. 58.6% 3c. 54.3% vs. 44.6%  4a. 20.9 (3.3) vs. 24.8 (2.8) 4b. 28.6 (3.7) vs. 39.6 (2.3) 4c. 35.7 (2.9) vs. 22.8 (6.1)   5a. 15.5 (2.7) vs. 19.8 (2.6) 5b. 20.8 (3.4) vs. 23.2 (3.1) 5c. 19.4 (2.8) vs. 10.2 (1.7)    6Ia. 1 vs. 2 6IIa. 16 vs. 13 6IIb. 3 vs. 0 6IIc. 2 vs. 1 6IId. 0 vs. 3 6IIe. 2 vs. 1 6IIf. 1 vs. 2 6IIIa.12 vs. 2 6IIIb. 2 vs. 0 6IIIc. 1 vs. 0 6IIId. 1 vs. 0 6IIIe. 1 vs. 0 | … | 0 |
| McDonald  1976[89]  USA | **1. n/N, %, of events to which provider responded by ordering the required tests to monitor drug effects over 8 months (prespecified) 1a. Overall.** 1b. Renal function (blood urea nitrogen or creatinine). 1c. Serum potassium. 1d. Serum uric acid. 1e. Liver function (serum glutamic oxalacetic transaminase, alkaline phosphatase, or bilirubin) 1f. Haemoglobin or haematocrit. 1g. Leukocyte count. 1h. Serum sodium.  **2. n/N, %, of events (abnormal measures) to which provider responded by changing therapy appropriately over 8 months (prespecified) 2a. Overall  Medication / Abnormality / Suggested response.**  2b. Oral hypoglycaemic, triamterene, potassium chloride, digoxin, thiazide, tetracycline, aspirin, phenobarbitol, macrodantin, or phenothiazine / Last blood urea nitrogen >25 mg/dL, or last creatinine >2mg/dL / Reduce because of risk of overtreatment.  2c. Methyldopa / As 2b / As 2b.  2d. Digitoxin / As 2b / As 2b.  2e. Subtotal for renal protocols. 2f. Aspirin-containing compounds / Last haemoglobin <12g/dL, or last haematocrit <36% / Reduce because possible cause of bleeding.  2g. Triamterene, potassium chloride /Last potassium >5 meq/L / Reduce because cause of metabolic toxicity. 2h. Cardiac glycosides, potassium-wasting diuretics / Last potassium <3.5 meq/L / Change regimen because of metabolic toxicity.  2i. Furosemide / Last sodium <135 meq/L / Reduce because cause of metabolic toxicity.  2j. Antihypertensives / Last DBP >110 mmHg / Increase regimen because of undertreatment. 2k. Methyldopa / Last alkaline phosphatase >160 IU/L / Reduce because cause of hepatic toxicity. 2l. Thiazides / Last uric acid >9 mg/dL / Reduce because cause of metabolic toxicity. 2m. Subtotals for nonrenal protocols.  3. n/N, %, of events (abnormal measures) to which provider responded by changing therapy appropriately or repeating index measure over 8 months (not clearly prespecified). | 1a. 144/390, 36% vs. 45/402, 11%, *P*<.0001 1b. 76/204, 37% vs. 28/220, 14% 1c. 27/73, 36% vs. 7/68, 10% 1d. 22/65, 33% vs. 6/67, 9% 1e. 13/34, 38% vs. 2/25, 8% 1f. 2/9, 22% vs. 2/12, 16% 1g. 3/4,75% vs. ?/6 (NR) 1h. 1/1, 100% vs. ?/4 (NR)  2a. 31/110, 28% vs. 9/68, 13%, *P*<.026 2b. 13/52 vs. 7/34 2c. 0/13 vs. 1/3 2d. 0/7 vs. 0/4 2e. 13/72, 18% vs. 8/41, 19% 2f. 2/5 vs. 1/5 2g. 2/3 vs. 0/6 2h. 4/8 vs. 0/6 2i. NR vs. 0/2 2j. 8/14 vs. 0/7 2k. 0/1 vs. 0/1 2l. 2/7 vs. NR 2m. 18/38, 47% vs. 1/27, 4%, *P*<.0004  3. 63/110, 57% vs. 16/68, 23%, *P*<.0001 | ... | ... | + | … |
| **Studies of multi-faceted interventions** | | | | | | |
| Bertoni  2009[16, 21]  USA | 3-year follow-up 2. Appropriate lipid management (met 1 of 7 criteria based on LDL-C level and risk strata) (primary)  2a. Proportion of patients at baseline (n=842 vs. 855); Difference 2b. Proportion of patients at follow-up (n=709 vs. 771); Difference  **2c. Change from baseline to follow-up; Difference; intra-class correlation** 2d. Group difference in subgroup of 58 practices with both baseline and follow-up data. Not prespecified   3. Inappropriate prescription of lipid-lowering therapy (secondary). 3a. Proportion of patients at baseline (n=626 vs. 650); Difference 3b. Proportion of patients at follow-up (n=519 vs. 571); Difference 3c. Change from follow-up to baseline; Difference  4. Appropriate prescription of lipid-lowering therapy. (secondary) 4a. Proportion of patients at baseline (n=216 vs. 205); Difference 4b. Proportion of patients at follow-up (n=190 vs. 200); Difference 4c. Change from follow-up to baseline; Difference  Stratified subgroup analyses 5-7. Appropriate lipid management* of patient dyslipidaemia by Risk Category* 5. Low risk patients: baseline n=296 vs. 357; follow-up n=309 vs. 336 6. Intermediate low-risk or intermediate high-risk: baseline n=315 vs. 281; follow-up n=253 vs. 254 7. High risk patients: baseline n=231 vs. 217; follow-up n=147 vs. 181 a. Proportion of patients at baseline; Difference. b. Proportion of patients at follow-up; Difference c. Change from follow-up to baseline; Difference; intraclass correlation  *Risk category defined by Framingham risk score (history and 10-year risk of coronary heart disease [CHD])  (1) Low risk (0-1 risk factor for CHD); (2) intermediate low risk (≥2 risk factors and a 10 year risk of <10%) (3) intermediate high risk (≥2 risk factors and a 10 year risk of 10% to 20% )(4) high risk (CHD risk equivalent [diabetes, CHD, stroke, or peripheral vascular disease] and/or ≥2 risk factors with a 10 year risk of >20%) | 2a. 73.4% vs. 79.7%; -6.3; *P*=.02 2b. 72.3% vs. 68.9%; +3.4; *P*=.18 2c. -1.1 vs. -10.8; +9.7; *P*≤0.01; 0.01  2d. +9.2%, *P*=.02  3a. 6.6% vs. 4.2%; +2.4; *P*=.15 3b. 3.9% vs. 6.4%; -2.5; *P*=.07 3c. -2.7 vs. +2.2; -4.9; *P*=.01  4a. 38.8% vs. 45.3%; -6.5; *P*=.27 4b. 24.8% vs. 24.1%; +0.7; *P*=.88 4c. -14.0 vs. -21.2; +7.2; *P*=.37  5a. 91.4% vs. 94.1%; -2.7; *P*=.29 5b. 90.9% vs. 89.2%; +1.7; *P*=.49 5c. -0.5 vs. -4.9; +4.4; *P*=.21; 0.01  6a. 69.4% vs. 73.9%; -4.5; *P*=.60 6b. 70.3% vs. 62.6%; +7.7; *P*=.07 6c. +0.9 vs. -7.3; +8.2; *P*=.03; 0.01  7a. 47.5% vs. 55.6%; -8.1; *P*=.14 7b. 24.4% vs. 28.7%; -4.3; *P*=.41 7c. -23.1 vs. -26.9; +3.8; *P*=.65; 0.01 | ... | ... | + | … |
| Gilutz  2009[19]  Israel | Mean 21-month follow-up **1. Appropriate initiation, up-titration, or continuation of statin therapy; % (unclear if represents patients); difference; OR (unclear if lower & upper ranges represent 95% CIs) (primary).**  2. Appropriate uptitration in patients with LDL≥110 mg/dL, % (unclear if represents patients – component of primary).  **3. Rate of adequate lipoprotein monitoring: % (not clear if represents patients); OR (unclear if lower & upper ranges represent 95% CIs) (primary).**  4. Effect of intervention on monitoring in 3425 patients not rehospitalised, RR, CI (not clear if 95% CI) (not prespecified). | 1. 59.1% vs. 53.7%; 5.4% (2.5% drug initiation, 1.8% up-titration, and 1.1% avoiding drug cessation), P<.003; 1.232 (lower 1.112, upper 1.365), *P*=.001  2. 8.6% vs. 7.4%, *P=*NS  3. 54.8% vs. 48.7%, *P*<.001; 1.28 (lower 1.17, upper 1.41), *P*<.001  4. 1.423 (1.24 to 1.64), *P*<.0001  In favour of the CCDSS | Mean 21 month follow-up. **1. Change in LDL level (primary) in 52.5% of patients with initial LDL >120 mg/dL: Baseline/Final mean (SD), % reduction. Note: data for 38.5% of patients with initial LDL <110 mg/dL and 9% with initial LDL 110-120 mg/dL were not reported.**  2. Proportion of patients who are alive and have not had a cardiovascular rehospitalization during 1^st^ year, % (secondary). | 1. 145.5 (22.3) / 121.9 (34.2), 16.2% vs. 145.8 (22.9) / 124.3 (34.6), 14.8%, *P*<.02  2. 57.1% vs. 59.2%, *P*<.03  (Data and text not clearly consistent; could not confirm with author if numbers represent CV hospitalization-free survival or a composite of mortality and CV hospitalization). | + | + |
| Javitt  2008[27]  USA | Not prespecified. 1. Resolution rate for problems identified by care considerations over 1 y: %, difference (% improvement). **1a. Add a drug (n=601 total). 1b. Stop a drug (n=592 total).**  2. Resolution rate for 311 patients with a recommendation to use an ACE-I (based on HOPE trial; n=155 vs. 156) over 1 y.  Note: Number of care considerations issued differed between groups: 1299 vs. 1519. | 1a. 26.6% vs. 18%, 8.6% (48%), *P*≤.05 1b. 28% vs. 34%, -6% (-18%) , *P=*NS  2. 27% vs. 14% | ... | ... | + | … |
| Quinn  2008[29]  USA | Secondary **1. Medications intensified at 3 months (% patients). 2. Medication errors identified at 3 months (% patients).** | 1. 84.62% vs. 23.08%, *P*=.002 2. 53.38% vs. 0%, *P*=.002 | **Primary 1. Mean HbA1c levels; Baseline/follow-up at 3 months; difference.**  Prespecified 2. Medications self-care (mean days/week); Baseline/follow-up at 3 months.  3. Patients reporting improved confidence about diabetes control at 3 months.  Not specified 4. New depression diagnosis at 3 months (% patients). | 1. 9.51%/7.48% vs. 9.05%/8.37%; 2.03% vs. 0.68%, *P*<.04  2. 5.92/6.64 vs. 6.3/6.75, *P*=.495 3. 100% vs. 75%, *P*=.167 4. 9.09% vs. 20%, *P*=.37 | + | + |
| van Wyk  2008[31]  The Netherlands | Primary 12-mo follow-up  (auto alerting vs. on-demand vs. control) **2. Patients requiring treatment were who treated.**  2a. n/N (%) patients. **2b. RR (95% CI) adjusted for number of individual visits, CVD, diabetes mellitus and practice size 2bi. Auto alerting vs. control. 2bii. On-demand vs. control.** 2biii. Auto alerting vs. on-demand. | 2a. 801/1218 (65.7%) vs. 385/969 (39.7%) vs. 275/766 (35.9%) 2bi. 1.40 (1.15 to 1.70) 2bii. 1.19 (0.94 to 1.50) 2biii. 1.18 (0.96 to 1.45) | ... | ... | Auto, +  On-demand, 0 | … |
| Feldstein  2006b[40]  USA | At 6 months **1. % of participants who received only medication within 6 months of the start of the study (component of primary); p-value  1a. provider reminder + patient reminder vs. control 1b. provider reminder alone vs. control** 1c. provider reminder + patient reminder vs. provider reminder alone  Prespecified 2. Change in probability of osteoporosis medication prescription as predicted by linear model: coefficient (represents absolute change) (95% CI) 2a. Provider reminder + patient reminder vs. control  2b. Provider reminder vs. control  Positive value = increase in probability of event with CCDSS  Note: n’s for those receiving specified treatment can be calculated from article. | 1a. 10.1% vs. 4.0%; *P*<.01 1b. 11.9% vs. 4.0%; *P*<.01 1c. 10.1% vs. 11.9% ; *P*=.54  2a. 0.15 (0.05 to 0.26) 2b. 0.23 (0.12 to 0.33) | … | … | + | … |
| Kuilboer  2006[44]  The Netherlands | 1. Median of paired differences of Delta values (the difference between the intervention and baseline periods) (P-value) for each age group: 0-11, 12-39, 40-59, ≥60. **Not clearly pre-specified  1a. Number of antihistamines prescriptions 1b. Number of cromoglycate prescriptions 1c. Number of deptropine prescriptions 1d. Number of oral bronchodilators prescriptions 1e. Number of oral corticosteroids prescriptions** | 1a. 0.000 (0.875), 0.000 (0.500), -0.004 (0.080), -0.000 (0.317) 1b. 0.000 (0.144), -0.0004 (0.033), 0.000 (0.051), 0.000 (0.893) 1c. -0.003 (0.753), N/A, N/A, N/A 1d. 0.001 (0.807), 0.000 (0.655), 0.000 (0.121), 0.000 (0.225) 1e. -0.004 (0.050), -0.002 (0.836), -0.023 (0.109), -0.045 (0.679) | ... | ... | 0 | … |
| Lester  2006[45, 59]  USA | **1. Patients with changes in statin prescriptions at 1 month (primary), n/N, %. 2. Patients with changes in statin prescriptions at 12 months (primary), n/N, %.** 3. median interval (IQR) to first medication adjustment among patients with changes (months) (not prespecified)  4. median (IQR) time to first measured LDL after study initiation (not prespecified)  NOTE: the preliminary data in the 2004 paper reports 15 primary care providers and 256 pts randomized; 2006 publication only mentions 14 primary care providers and 235 patients, Author indicated that 1 physician (centre) was lost during the study, hence different numbers.  2004 also reports 1 outcome not in 2006 paper – looks like 1 mo follow-up (but not explicit): Patients with repeat fasting lipid profile ordered. 12.9% vs. 7.6%, *P*=.16 | 1. 18/118, 15.3% vs. 2/117, 2%, *P*=.001 2. 29/118, 24.6% vs. 20/117, 17.1%, *P*=.14 3. 0 (0 to 8.5) vs. 7.1 (3.9 to 10.4), *P*=.005  4. 99 (48 to 171) vs. 121 (45 to 208), *P*=.48 | **1. Patients with change in LDL levels of all patients with LDL results (primary), n/N, %** 2. mean (SD) first LDL level after intervention (part of primary) 3. mean (SD) final LDL level (part of primary)  Prespecified subgroup analysis. 4. Patients with LDL cholesterol level > 130mg/dL at baseline, n/N, %. 5. Of patients with LDL>130 mg/dL at baseline, mean (SD) first LDL after intervention. 6. Of patients with LDL>130 mg/dL at baseline, mean (SD) final LDL level. | 1. 81/118, 68.6% vs. 82/117, 82%, *P*=.8 2. 111.7 (30.2) vs. 118.1 ( 32.1), *P*=.2 3. 106.8 (26.8) vs. 111.5 (30.0), *P*=.3  4. 41/118, 34.7% vs. 39/117, 33.3%, *P*=.9 5. 119 (32.1) vs. 138 (35.6), *P*=.04 6. 111.4 (29.3) vs. 128.3 (35.7), *P*=.055 | + | 0 |
| Cobos  2005[49]  Spain | Mean follow-up 12.2 vs. 11.2 months **All secondary  1. Mean number of assessments. 1a. AST/ALT measurements. 1b. Creatine kinase; determinations.**  **2. Number (%) patients treated with LLDs; odds ratio (95% CI). 2a. Patients with CHD. 2b. High-risk patients without CHD. 2c. Low-risk patients without CHD.** | 1a. 1.41 vs. 1.31, *P*=.033 1b. 0.54 vs. 0.24, *P*=.053  2a. 102 (92.7%) vs. 125 (85.0%); 2.54 (0.92 to 6.98) 2b. 201 (70.5%) vs. 260 (76.9%); 0.69 (0.44 to 1.06) 2c. 124 (19.0%) vs. 292 (44.2%); 0.25 (0.16 to 0.41) | Mean follow-up 12.2 vs. 11.2 months. Primary outcome & analysis: **1. n/N (%) patients with successful management* in ITT analysis; difference (95% lower confidence limit); odds ratio (95% CI).** Primary outcome – sensitivity analysis 2. n/N (%) patients with successful management* in per-protocol analysis (≥1 post-baseline assessment); difference (95% lower confidence limit); odds ratio (95% CI). Primary outcome – sensitivity analysis 3. n/N (%) patients with successful management* in per-protocol analysis (≥9 months follow-up); difference (95% lower confidence limit); odds ratio (95% CI).  Not clear if subgroup analyses prespecified. 4. Proportion of patients with successful management (ITT: 1046 vs. 1145 patients). 4a. Patients with coronary heart disease (CHD) and no previous lipid-lowering drug (LLD) treatment. 4b. Patients with CHD and previous LLD treatment. 4c. High-risk patients without CHD and no previous LLD treatment. 4d. High-risk patients without CHD and previous LLD treatment. 4e. Low-risk patients without CHD and no previous LLD treatment. 4f. Low-risk patients without CHD and previous LLD treatment. Secondary outcomes  5. Mean final lipid values (mg/dL); difference (95% CI) (ITT: 1046 vs. 1145 patients). 5a. Total cholesterol. 5b. LDL-cholesterol. 5c. High-density lipoprotein-cholesterol. 5d. Triglycerides. Management success:  *If cardiovascular risk ≥20% over 10 yrs, success = LDL-C < 115mg/dL at study end for patients with CHD or < 130mg/dL for those without CHD. If cardiovascular risk <20% over 10 years, success = risk still <20% at study end. | 1. 565/1046 (54.02%) vs. 578/1145 (50.48%); 3.53% (-4.97)*; 1.02 (0.58 to 1.77) 2. 516/789 (65.40%) vs. 526/832 (63.22%); 2.18% (-3.96)*; 1.06 (0.72 to 1.55) 3. 422/620 (68.06%) vs. 356/544 (65.44%); 2.62 (-3.21)*; 1.12 (0.72 to 1.76) *Lower CI <-5% meets non-inferiority criterion.  4a. 23.69% vs. 23.39%, *P=*NS 4b. 22.26% vs. 21.98%, *P=*NS 4c. 21.53% vs. 21.25%, *P=*NS 4d. 20.20% vs. 19.94%, *P=*NS 4e. 73.68% vs. 73.36% , *P=*NS 4f. 72.09% vs. 71.76%, *P=*NS Note: No significant interactions for group by CV risk level or group by previous LLD treatment.  5a. 233.8 vs. 231.0; 2.8 (-1.7, -7.3); *P*=.218 5b. 149.2 vs. 146.5; 2.7 (-1.7, -7.1); *P*=.227 5c. 58.0 vs. 56.3; 1.6 (-0.6, -3.6); *P*=.142 5d. 136.6 vs. 135.2; 1.4 (-8.3, -11.2); *P*=.766 | 0 | 0 |
| Javitt  2005[52]  USA | 1. % physician compliance with recommendations over 12 months; relative (%) difference (components of primary). **1a. Recommendations to add a drug. 1b. Recommendations to discontinue a medication.** | 1a. 24% vs. 17%; 42% (*P*=.007). 1b. unable to assess. | N = 19,739 vs. 19,723 patients. 1. Hospital utilization over 12 months (prespecified) **1a. Admissions per 1000 persons, mean ± SD; difference. 1b. Inpatient days per 1000 persons, mean ± SD; difference. 1c. Mean hospital length of stay in days; % difference. 1d. Total number of hospital admissions.**  2. Mortality (not prespecified).  Subgroup analyses of patients who triggered recommendations (both interventions [n=961] and control [n=982]):  3. Hospital utilization over 12 months 3a. Admissions per 1000 persons, mean ± SD; difference.  3b. Inpatient days per 1000 persons, mean ± SD; difference. 3c. Mean hospital length of stay in days; % difference. 3d. Total number of hospital admissions.  Subgroup analyses for patients with HOPE trial-consistent recommendation for ACE-I prescription (n=156 vs. 155 patients).  4. Total hospital admissions over 12 months.  5. HOPE-related hospital utilization over 12 months. 5a. Hospital admissions, n (%). 5b. Inpatient days per person, mean. 6. Non-HOPE-related hospital utilization over 12 months. 6a. Hospital admissions, n (%). 6b. Inpatient days per person, mean. | 1a. 63.5 ± 3.4 vs. 69.3 ± 3.4; -9.1% (*P* =.03). 1b. 247.7 ± 6.0 vs. 273.0 ± 6.2; -9.3% (*P* =.001). 1c. 4.1 vs. 4.1 1d. 1251 vs. 1366; 115  2. Data NR; NS overall and for in-hospital mortality.   3a. 213.8 ± 5.7 vs. 264.6 ± 5.7; -19.2% (*P*<.001). 3b. 1152.0 ± 45.0 vs. 1252.3 ± 47.0; -8.0% (*P*=.004). 3c. 5.4 vs. 4.7; 13.8% (NS). 3d. 106 vs. 302  4. 133 vs. 152. 5a. 49 (36.8%) vs. 69 (45.4%), *P*=.02 5b. 1.4 vs. 2.2, *P*=.003  6a. 84 (53.8%) vs. 83 (53.5%), *P*=.55 6b. 3.3 vs. 3.8, *P*=.34 | + | + |
| Plaza  2005[53]  Spain | **Prespecified; 12-mo follow-up. 1. Prescriptions of oral glucocorticoids** | 1. 130 vs. 727, *P*=.0135 | 12-mo follow-up Primary prespecified outcome. 1. Estimated increment of the cost-effectiveness coefficient (primary)  1a. From the social perspective.  1b. From the perspective of the one who pays.  **2. St. George Respiratory Questionnaire score (score range 0 [no impairment] to 100 [maximum impairment]):  2a. Total score (SE); difference (95% CI)** 2b. Activity (SE); difference (95% CI) 2c. Symptoms (SE); difference (95% CI) 2d. Impact (SE); difference (95% CI)  Prespecified  3. Number of emergency room visits. 4. Number of hospitalizations. 5. Days spent in ICU. 6. Days hospitalised. 7. Days on rescue medication.  8. Number of short cycles of oral steroid use.  9. Number of patients symptom-free at the end of the study. | 1a. -135 per each point reduction in the SGRQ scale (95% CI -8374 to -38)  1b. -61 (95% CI -2323 to -14).  The negative sign of the coefficients was due to a minor cost and major reduction of the SGRQ in intervention group compared to the usual care group.  2a. 27.3 (2.0) vs. 34.1 (1.9), *P*=.002; 6.8 (2.5 to 11.1) 2b. 35.6 (2.9) vs. 44.4 (2.9), *P*=.005; 8.8 (2.7 to 14.8) 2c. 32.9 (1.9) vs. 39.7 (1.8), *P*=.003; 6.8 (2.3 to 1.3) 2d. 20.7 (2.0) vs. 26.3 (1.9), *P*=.001; 5.6 (1.2 to 10.1)  3. 49 vs. 115, *P*=.0888 4. 12 vs. 15, *P*>.10 5. 8 vs. 2, *P*>.10 6. 37 vs. 166, *P*>.10 7. 3,478 vs. 9,318, *P*=.0257 8. 53 vs. 95, *P*>.10  9. 49 vs. 22 | + | + |
| Sequist  2005[55]  USA | **Prespecified**  **1. Receipt of recommended components of diabetes care during the 6 mo study; hazard Ratio (95% CI): 2a. Hypertension/ACE-I use  2b. Statin use for LDL cholesterol ≥ 130 mg/dL   3. Receipt of recommended components of coronary artery disease care during the 6 mo study; hazard Ratio (95% CI): 3a. Aspirin use 3b. β-blocker use  3c. Statin use for LDL cholesterol ≥130 mg/dL**  Hazard Ratio>1 = benefit for CCDSS | 2a. 1.42 (0.94 to 2.14), *P*=.10 2b. 1.10 (0.65 to 1.85), *P*=.73  3a. 2.36 (1.37 to 4.07), *P*=.002 3b. 1.09 (0.72 to 1.63), *P*=.69 3c. 1.51 (1.05 to 2.17), *P*=.03 | ... | ... | 0 | … |
| Tierney  2005[56]  USA | Physician intervention vs. pharmacist intervention vs. both interventions vs. control: Number of patients/grp, 194 vs. 161 vs. 182 vs. 169  Components of primary outcome 1. Number of suggestions adhered to/Number of patients with suggestions, %, of care suggestions adhered to over 3 yrs. **a. Start ipratropium.  b. Start inhaled β-agonist.  c. Switch to cheaper β-agonist.  d. Increase/decrease theophylline dose.  e. Stop ipratropium.  f. Start inhaled corticosteroid.  g. Start oral corticosteroid.**  **Prespecified**  **2. Medication compliance measures.  a. Mean Inui score (%).  b. Mean (SD) Morisky score.  c. N, %, of patients with ≥2 prescription refills.  d. Mean (SD) medication possession ratio (measure referenced but not described).**  **3. Mean (SD) score for patient satisfaction with physician (American Board of Internal Medicine questionnaire; score range/direction not described).  4. Mean (SD) score for patient satisfaction with pharmacist (American Board of Internal Medicine questionnaire; score range/direction not described).** | 1a. 30/71, 42% vs. 15/59, 25% vs. 23/65, 35% vs. 17/67, 25%, *P*=NS  1b. 18/30, 60% vs. 13/25, 52% vs. 16/24, 67% vs. 23/33, 70%, *P*=NS  1c. 23/30, 77% vs. 13/20, 65% vs. 30/33, 91% vs. 17/24, 71%, *P*=NS 1d. 26/39, 67% vs. 18/25, 72% vs. 20/31, 65% vs. 16/24, 67%, *P*=NS 1e. 7/22, 32% vs. 10/18, 56% vs. 16/28, 57% vs. 12/21, 57%, *P*=NS 1f. 2/18, 11% vs. 3/10, 30% vs. 3/11, 27% vs. 1/9, 11%, *P*=NS 1g. 5/10, 50% vs. 2/4, 50% vs. 3/9, 33% vs. 2/9, 22%, *P*=NS  All *P*=NS unless noted otherwise.  2a. 81% vs. 80% vs. 82% vs. 80%  2b. 0.95 (1.1) vs. 0.85 (1.0) vs. 0.89 (1.1) vs. 0.88 (1.0) 2c. 128, 95% vs. 89, 81% vs. 109, 92% vs. 96, 87% 2d. 0.98 (0.8) vs. 1.00 (2.7) vs. 1.1 (2.0) vs. 0.92 (1.0)  3. 1.9 (0.9) vs. 2.0 (0.9) vs. 2.1 (0.6) vs. 2.1 (0.7), *P*=NS 4. 2.1 (0.7) vs. 2.1 (0.8) 2.0 (0.6) vs. 2.1 (0.7), *P*=NS | Physician intervention vs. pharmacist intervention vs. both interventions vs. control  All prespecified with follow-up at 12 mo. **1. Mean (SD) SF-36 subscale scores (N/grp: 135 vs. 110 vs. 118 vs. 111). Higher scores better.  a. Physical function.  b. Role physical.  c. Pain.  d. General health.  e. Vitality.  f. Social function.  g. Role emotional.  h. Mental health.   2. Mean (SD) McMaster Asthma Quality of Life Questionnaire subscale scores (N/grp: 38 vs. 31 vs. 27 vs. 20). Higher scores better.  a. Overall health status.**  b. Activity.  c. Symptoms.  d. Emotion.  e. Environment.   **3. Mean (SD) McMaster Chronic Respiratory Disease Questionnaire subscale scores (N/grp: 72 vs. 104 vs. 91 vs. 91). Higher scores better.  a. Overall health status.**  b. Dyspnoea.  c. Fatigue.  d. Emotion.  e. Mastery.   **4. Mean (SD) number of emergency department visits.  a. For any reason.**  b. For reactive airways disease.  **5. Mean (SD) number of hospitalizations. a. For any reason.**  b. For reactive airways disease. | All *P*=NS unless noted otherwise. 1a. 38 (23) vs. 38 (27) vs. 36 (24) vs. 37 (26)  1b. 32 (40) vs. 33 (40) vs. 38 (41) vs. 32 (40), *P*<.05 in favour of both interventions 1c. 49 (25) vs. 47 (27) 48 (26) vs. 44 (26) 1d. 37 (24) vs. 29 (25) vs. 35 (20) vs. 34 (22) 1e. 37 (21) vs. 39 (23) vs. 36 (23) vs. 36 (20) 1f. 69 (27) vs. 63 (30) vs. 61 (29) vs. 63 (29)  1g. 65 (43) vs. 60 (44) vs. 59 (43) vs. 60 (45) 1h. 62 (23) vs. 62 (23) vs. 50 (25) vs. 61 (24)  2a. 4.0 (1.5) vs. 4.2 (1.4) vs. 4.2 (1.1) vs. 3.7 (1.3) 2b. 4.5 (1.5) vs. 4.6 (1.3) vs. 4.4 (1.2) vs. 3.9 (1.2)  2c. 4.0 (1.5) vs. 4.0 (1.5) vs. 4.2 (1.2) vs. 3.6 (1.4) 2d. 3.8 (2.0) vs. 4.3 (1.6) vs. 4.4 (1.2) vs. 3.6 (1.5), *P*<.05 in favour of pharmacist intervention 2e. 3.9 (1.6) vs. 4.2 (1.5) vs. 4.0 (1.4) vs. 3.7 (1.4)   3a. 4.4 (1.2) vs. 4.3 (1.3) vs. 4.1 (1.1) vs. 4.2 (1.1) 3b. 4.2 (1.6) vs. 4.2 (1.7) vs. 4.0 (1.6) vs. 4.0 (1.5) 3c. 3.8 (1.3) vs. 3.7 (1.5) vs. 3.4 (1.2) vs. 3.6 (1.2) 3d. 4.6 (1.3) vs. 4.5 (1.4) vs. 4.2 (1.2) vs. 4.4 (1.3) 3e. 4.8 (1.4) vs. 4.8 (1.5) vs. 4.5 (1.4) vs. 4.6 (1.4)  4a. 1.4 (1.7) vs. 1.5 (2.3) vs. 1.4 (2.1) vs. 1.4 (1.9) 4b. 0.3 (0.7) vs. 0.4 (0.8) vs. 0.4 (0.8) vs. 0.3 (0.8) 5a. 0.5 (1.6) vs. 0.5 (1.1) vs. 0.4 (1.1) vs. 0.4 (0.8) 5b. 0.1 (0.5) vs. 0.1 (0.5) vs. 0.1 (0.5) vs. 0.1 (0.3) | 0 | 0 |
| Wolfenden  2005[57]  Australia | 6-mo trial Components of primary outcome. 1. Receipt of elements of cessation care: n/N patients, %; OR (95% CI). **1a. Preoperative NRT offered: self-report. 1b. Preoperative NRT offered: medical audit. 1c. Postoperative NRT prescribed: medical audit.**  **2. Annual incremental cost of sustaining comprehensive cessation care: Australian dollars (prespecified).** | 1a.60/73, 82% vs. 4/50, 8%; 53.1 (16.2 to 173.5) *P*<.01 1b. 79/89, 89% vs. 0/56, 0%; 855.6 (49.1 to infinity) *P*<.01 1c. 61/71, 86% vs. 0/37, 0%; 439.2 (25.0 to infinity), *P*<.01  2. Australian $14,681 or $35/smoking patient. | ... | ... | + | … |
| Murray  2004[60]  USA | **All reported as pharmacist vs. physician vs. pharmacist + physician vs. control groups at 12 months (n=180 vs. 181 vs. 180 vs. 171 patients): 1. Compliance with treatment suggestions (secondary): n (%) patients with suggestions; mean (SD) adherence rate. a. All antihypertensive drug suggestions.** b. Start or increase ACE-I. c. Start diuretic. d. Start or increase calcium channel blocker. e. Start or increase β-blocker.  **2. Patient satisfaction with physicians and pharmacists (secondary).**  Not prespecified 3. Total number of antihypertensive drug suggestions/mean (SD) per patient. | 1a. 117 (65%) vs. 123 (68%) vs. 125 (69%) vs. 114 (67%); 25 (33) vs. 29 (36) vs. 35 (39) vs. 26 (33); *P*=.13 1b. 89 (42%) vs. 92 (51%) vs. 96 (53%) vs. 91 (53%); 33 (47) vs. 44 (50) vs. 41 (49) vs. 30 (46); *P*=NS 1c. 54 (30%) vs. 55 (30%) vs. 52 (29%) vs. 58 (34%); 22 (42) vs. 22 (42) vs. 25 (44) vs. 31 (47); *P*=NS 1d. 38 (21%) vs. 56 (31%) vs. 46 (26%) vs. 51 (30%); 47 (51) vs. 34 (48) vs. 39 (49) vs. 49 (51); *P*=NS 1e. 35 (14%) vs. 31 (17%) vs. 34 (19%) vs. 20 (12%); 29 (46) vs. 45 (51) vs. 47 (51) vs. 45 (51); *P*=NS  2. No data reported.  3. 234/2.0 (1.1) vs. 255/2.1 (1.1) vs. 243/1.9 (1.0) vs. 245/2.1 (1.1) | **All reported as pharmacist vs. physician vs. pharmacist + physician vs. control groups at 12 months.  1. Mean (SD) overall composite quality of life score (primary). (n=116 vs. 124 vs. 116 vs. 127 patients)**  All other outcomes were secondary. 2. Mean (SD) short-form 36 subscale scores (n=116 vs. 124 vs. 116 vs. 127 patients). 2a. Physical function. 2b. Role physical. 2c. Pain. 2d. General health. 2e. Vitality. 2f. Social function. 2g. Role emotional. 2h. Mental health.  3. Bulpitt subscales (%) (n=116 vs. 124 vs. 116 vs. 127 patients). 3a. Faint. 3b. Faint on standing. 3c. Faint in the morning. 3d. Sleepy. 3e. Weak. 3f. Blurry vision. 3g. Short of breath. 3h. Swollen ankles. 3i. Walk slowly. 3j. Loose bowel movements. 3k. Dry mouth 3l. Dysphagia. 3m. Bad taste in mouth. 3n. Runny nose. 3o. Poor concentration. 3p. Flushing of face or neck. 3q. Nightmares. 3r. Nausea or vomiting. 3s. Rash. 3t. Itching. 3u. White fingers. 3v. Finger pain. 3w. Headache. 3x. Dry cough. 3y. Libido decreased. 3z. Erectile dysfunction.  4. Mean (SD) number of emergency department visits per patient (n=180 vs. 181 vs. 180 vs. 171 patients).  4a. All.  4b. Heart disease specific.   5. Mean (SD) number of hospitalizations per patient (n=180 vs. 181 vs. 180 vs. 171 patients). 5a. All.  5b. Heart disease specific.  6. Mean (SD) systolic BP (mm Hg) (n=128 vs. 126 vs. 129 vs. 124 patients). 6a. Baseline.  6b. Last 6 months.  7. Mean (SD) diastolic BP (mm Hg) (n=128 vs. 126 vs. 129 vs. 124 patients).  7a. Baseline. 7b. Last 6 months.   Not prespecified 8. Deaths (n=180 vs. 181 vs. 180 vs. 171 patients). | 1. 37 (21) vs. 35 (20) vs. 38 (22) vs. 36 (21); *P*=NS  2a. 48 (29) vs. 52 (28) vs. 45 (30) vs. 49 (28), *P*=NS 2b. 53 (41) vs. 49 (42) vs. 46 (44) vs. 44 (44), *P*=NS 2c. 51 (29) vs. 53 (27) vs. 45 (28) vs. 48 (27), *P*=NS 2d. 46 (23) vs. 51 (24) vs. 45 (24) vs. 46 (24), *P*=NS 2e. 46 (21) vs. 48 (23) vs. 43 (24) vs. 45 (23), *P*=NS 2f. 72 (29) vs. 75 (27) vs. 68 (32) vs. 70 (29), *P*=NS 2g. 66 (43) vs. 70 (41) vs. 64 (44) vs. 66 (43), *P*=NS 2h. 66 (23) vs. 70 (21) vs. 62 (24) vs. 65 (22), *P*=NS  3a. 42% vs. 43% vs. 47% vs. 42%, *P*=NS 3b. 17% vs. 19% vs. 23% vs. 23%, *P*=NS 3c. 22% vs. 12% vs. 14% vs. 18%, *P*=NS 3d. 72% vs. 71% vs. 73% vs. 75%, *P*=NS 3e. 52% vs. 59% vs. 61% vs. 54%, *P*=NS 3f. 38% vs. 40% vs. 44% vs. 38%, *P*=NS  3g. 49% vs. 36% vs. 45% vs. 44%, *P*=NS 3h. 51% vs. 46% vs. 49% vs. 43%, *P*=NS 3i. 45% vs. 42% vs. 47% vs. 39%, *P*=NS 3j. 46% vs. 40% vs. 44% vs. 38%, *P*=NS 3k. 49% vs. 49% vs. 59% vs. 50%, *P*=NS 3l. 24% vs. 20% vs. 29% vs. 28%, *P*=NS 3m. 45% vs. 40% vs. 43% vs. 48%, *P*=NS 3n. 53% vs. 53% vs. 58% vs. 54%, *P*=NS 3o. 22% vs. 18% vs. 21% vs. 23%, *P*=NS 3p. 20% vs. 20% vs. 23% vs. 22%, *P*=NS 3q. 31% vs. 30% vs. 34% vs. 35%, *P*=NS 3r. 32% vs. 26% vs. 26% vs. 25%, *P*=NS 3s. 15% vs. 16% vs. 18% vs. 16%, *P*=NS 3t. 30% vs. 36% vs. 44% vs. 37%, *P*=NS 3u. 25% vs. 17% vs. 22% vs. 17%, *P*=NS 3v. 13% vs. 13% vs. 15% vs. 13%, *P*=NS 3w. 52% vs. 46% vs. 49% vs. 51%, *P*=NS 3x. 37% vs. 37% vs. 37% vs. 34%, *P*=NS 3y. 34% vs. 40% vs. 29% vs. 28%, *P*=NS 3z. 33% vs. 41% vs. 42% vs. 42%, *P*=NS  4a. 1.11 (1.94) vs. 1.02 (1.67) vs. 1.01 (3.03) vs. 1.21 (2.04); *P*=NS 4b. 0.02 (0.13) vs. 0.01 (0.07) vs. 0.01 (0.07) vs. 0.04 (0.20); *P*=.02 for intervention groups vs. control group  5a. 0.25 (0.62) vs. 0.25 (0.69) vs. 0.19 (0.74) vs. 0.25 (0.89); *P*=NS 5b. 0.01 (0.07) vs. 0.01 (0.10) vs. 0.01 (0.11) vs. 0.02 (0.13); *P*=NS 6a. 144 (18) vs. 143 (20) vs. 143 (17) vs. 142 (16); *P*=NS 6b. 144 (21) vs. 144 (18) vs. 142 (23) vs. 143 (18); *P*=NS  7a. 78 (10) vs. 75 (12) vs. 76 (11) vs. 78 (10); *P*=NS 7b. 77 (11) vs. 75 (12) vs. 77 (14) vs. 78 (11); *P*=NS  8. 1% vs. 2% vs. 1% vs. 1% | 0 | 0 |
| Tierney  2003[66]  USA | Components of primary outcome (Physician intervention vs. pharmacist intervention vs. both intervention vs. control) 1. Adherence with care suggestions over 12 months. **1a. Start or increase an ACE-I. 1b. Start or increase a β-blocker. 1c. Start low-dose aspirin. 1d. Start or increase a diuretic. 1e. Start or increase a long-acting nitrate. 1f. Start an antihyperlipidemic drug. 1g. Start or increase a calcium blocker.**  **2. Medication compliance over 12 months (secondary).**  **3. Patient satisfaction with physicians over 12 months(secondary). 4. Patient satisfaction with pharmacist over 12 months(secondary).** | 1a-1g. n/N patients (%) 1a. 41/109 (38%) vs. 40/92 (44%) vs. 39/94 (42%) vs. 39/107 (36%), *P*>.2 1b. 15/96 (16%) vs. 11/76 (14%) vs. 18/91 (20%) vs. 10/83 (12%), *P*>.2 1c. 18/74 (24%) vs. 17/72 (24%) vs. 13/68 (19%) vs. 23/81 (28%), *P*>.2 1d. 17/71 (24%) vs. 11/53 (21%) vs. 13/62 (21%) vs. 20/73 (27%), *P*>.2 1e. 6/30 (20%) vs. 8/34 (24%) vs. 8/44 (18%) vs. 3/25 (12%), *P*>.2 1f. 7/22 (32%) vs. 5/15 (33%) vs. 11/22 (50%) vs. 8/22 (36%), *P*>.2 1g. 7/21 (33%) vs. 5/13 (39%) vs. 6/23 (26%) vs. 10/17 (59%), *P*>.2  2. Data not reported, *P*>.69  3. Data not reported, *P*>.5 4. Data not reported, *P*>.4 | Physician intervention vs. pharmacist intervention vs. both intervention vs. control  **1. Mean (SD) quality of life (score SF-36) at 12 mo (primary).  1a. Physical function 1b. Role physical 1c. Pain 1d. General health 1e. Vitality 1f. Social function 1g. Role emotional 1h. Mental health  2. Mean (SD) quality of life (Chronic heart disease questionnaire subscale scores) at 12 months (primary). 2a. Overall health status**  2b. Dyspnoea 2c. Fatigue 2d. Emotion  3. Mean (SD) number of emergency department visits over 12 months (secondary). 3a. All. 3b. Heart disease specific.  4. Mean (SD) number of hospitalizations over 12 months (secondary). a. All. b. Heart disease specific.  5. Mortality over 12 months (not prespecified). | 1a. 36 (27) vs. 38 (26) vs. 39 (27) vs. 42 (26), *P*=NS 1b. 35 (40) vs. 37 (41) vs. 40 (42) vs. 43 (42), *P*=NS 1c. 47 (28) vs. 53 (29) vs. 52 (27) vs. 53 (28), *P*=NS 1d. 38 (22) vs. 41 (24) vs. 39 (22) vs. 42 (24), *P*=NS 1e. 40 (23) vs. 40 (25) vs. 44 (24) vs. 44 (25), *P*=NS 1f. 65 (30) vs. 66 (31) vs. 64 (32) vs. 69 (28), *P*=NS 1g. 61 (46) vs. 64 (44) vs. 71 (43) vs. 61 (44), *P*=NS 1h. 64 (22) vs. 64 (23) vs. 65 (24) vs. 63 (25), *P*=NS 2a. 4.5 (1.2) vs. 4.6 (1.2) vs. 4.6 (1.3) vs. 4.6 (1.2), *P*=NS 2b. 5.0 (1.5) vs. 5.3 (1.5) vs. 5.2 (1.6) vs. 5.2 (1.4), *P*=NS 2c. 3.8 (1.4) vs. 3.8 (1.5) vs. 4.0 (1.5) vs. 4.0 (1.3), *P*=NS 2d. 4.5 (1.3) vs. 4.6 (1.4) vs. 4.7 (1.4) vs. 4.6 (1.4), *P*=NS 3a. 1.1 (1.9) vs. 1.1 (1.8) vs. 1.1 (1.4) vs. 1.0 (1.7), *P*=NS 3b. 0.2 (0.4) vs. 0.2 (0.6) vs. 0.1 (0.4) vs. 0.2 (0.5), *P*=NS  4a. 0.4 (1.0) vs. 0.5 (1.0) vs. 0.5 (1.1) vs. 0.5 (1.1), *P*=NS 4b. 0.2 (0.6) vs. 0.2 (0.7) vs. 0.2 (0.6) vs. 0.2 (0.5), *P*=NS   5. Data not reported (2% overall), *P*>.9 | 0 | 0 |
| Eccles  2002[69, 64]  UK | **Prespecified (adherence) 1. Drugs prescribed for patients with angina (n=2881; n=1415 computerised system, n=1466 controls) proportion of patients 12 months before/12 months after intervention period; odds ratio (95%CI). 1a.** β**-blockers.** | 1a. 47%/48% vs. 49%/49%; 0.99 (0.73 to 1.33) | Prespecified – angina patients **1. Change in overall quality of life (SF-36 and EQ-5D questionnaires) from 12 months before to 12 months after intervention. 2. Change in disease-specific quality of life (Seattle angina questionnaire) from 12 months before to 12 months after intervention.** | 1. No difference between groups (data not reported) 2. No difference between groups (data not reported) | 0 | 0 |
| Flottorp  2002[70, 63]  Norway | Primary outcomes for sore throat (evaluated for 18 wks before and after the intervention) **1. Use of antibiotics: % at follow-up (n/N), % change from baseline, % difference; intracluster correlation coefficient (95%), *P* value.**  Primary outcomes for urinary tract infection (evaluated for 18 wks before and after the intervention) **2. Use of antibiotics: % at follow-up (n/N), % change from baseline, % difference; intracluster correlation coefficient (95%), *P* value.** | 1. 43.8% (2202/5031) vs. 49.5% (1552/3135), -4.3% vs. -1.3%, 3.0% 0.085 (0.056 to 0.114), P=.032  2. 46.3% (1167/2522) vs. 43.4% (1285/2961), -0.2% vs. 0.2%, 0.4%; 0.085 (0.057 to 0.113), *P*=.639  Note: Variations in rates of antibiotic use are also displayed in figure 2 p.4. | ... | ... | + | … |
| Lesourd  2002[71]  France | … | … | Follow-up period NR **1. Patient pregnancy rate (primary), n/N (%).** 1a. Clinical pregnancies. 1b. Ongoing pregnancies.   Subgroup analysis by expected response to FSH stimulation (response defined p.457 of paper; number of patients in each subgroup NR). 2. Patient clinical pregnancy rate: n (% of cycles). 2a. Poor responders. 2b. Normal responders. 2c. High responders. | 1a. 15/82 (18%) vs. 13/82 (16%), *P*=NS 1b. 13/82 (16%) vs. 12/82 (15%), *P*=NS  2a. 4 (29%) vs. 1 (6%) 2b. 9 (15%) vs. 12 (20%) 2c. 2 (25%) vs. 0% | … | 0 |
| Selker  2002[72]  USA | No clearly pre-specified outcomes – subgroup analyses not pre-specified.  **1a. Number of patients who had ST-segment elevation detected but did not have AMI. 1b. Number (%) of patients in 1a who received thrombolytic therapy 1c. Number (%) of patients who received thrombolytic therapy and had contraindications  2. The effect of the CCDSS (TPI) on treatment of patients with acute myocardial infarction: % of patients, Relative Risk (95% CI) (adjusted), P-value 2a. all patients; thrombolytic therapy within 1 hour**  2b. all patients; thrombolytic therapy  2c. patients with inferior AMI; thrombolytic therapy within 1 hour 2d. patients with inferior AMI; thrombolytic therapy 2e. patients with anterior AMI; thrombolytic therapy within 1 hour 2f. patients with anterior AMI; thrombolytic therapy   3. The effect of the CCDSS (TPI) on treatment of patients with acute myocardial infarction: % of patients, Relative Risk (95% CI) (adjusted), P-value 3a. women; thrombolytic therapy within 1 hour 3b. women; thrombolytic therapy 3c. men; thrombolytic therapy within 1 hour 3d. men; thrombolytic therapy   4. The effect of the CCDSS (TPI) on treatment of patients with acute myocardial infarction for whom physician consultation was entirely by telephone: % of patients, Relative Risk (95% CI) (adjusted), P-value 4a. thrombolytic therapy within 1 hour 4b. thrombolytic therapy   5. The effect of the CCDSS (TPI) on treatment of patients with acute myocardial infarction who presented to hospitals without an on-site emergency department physician: % of patients, Relative Risk (95% CI) (adjusted), P-value 5a. thrombolytic therapy within 1 hour 5b. thrombolytic therapy | 1a. 208 vs. 191 1b. 3 (1.4%) vs. 1 (0.5%), *P*>.2 1c. 1 (0.3%) vs. 2 (0.6%), *P*>.2  2a. 53.3% vs. 52.5%, 1.0 (0.9 to 1.2), *P*>.2 2b. 62.1% vs. 60.5%, 1.1 (0.96 to 1.1), *P*=.2  2c. 58.6% vs. 53.2%, 1.1 (0.9 to 1.3), *P*=.08 2d. 67.6% vs. 61.1%, 1.1 (1.01 to 1.2), *P*=.03  2e. 45.3% vs. 51.4%, 0.9 (0.8 to 1.1), *P*>.2 2f. 53.9% vs. 59.5%, 0.9 (0.8 to 1.1), *P*>.2   3a. 48.4% vs. 40.5%, 1.2 (0.96 to 1.5), *P*=.10 3b. 58.2% vs. 48.1%, 1.2 (1.01 to 1.5), *P*=.03 3c. 55.9% vs. 58.0%, 1.0 (0.9 to 1.1), *P*>.2 3d. 64.2% vs. 66.2%, 1.0 (0.9 to 1.1), *P*>.2  4a. 53.6% vs. 41.1%, 1.3 (1.01 to 1.7), *P*=.04 4b. 63.2% vs. 47.3%, 1.3 (1.2 to 3.1), *P*=.01  5a. 58.8% vs. 40.9%, 1.4 (0.8 to 2.6), *P*=.19 5b. 76.5% vs. 50.0%, 1.5 (0.97 to 2.4), *P*=.04 | **1. Proportion of patients who died within 30 day follow-up (P-value) 2. Number (%) of strokes within 30 day follow-up (P-value).  3. Number (%) of thrombolysis-related bleeding vents that required transfusion during the 30 day follow-up (P-value).** | 1. 5.0 vs. 3.4 (*P*= 0.15) 2. 3 (0.5%) vs. 3 (0.5%) (*P*> 0.2) 3. 22 (5.8%) vs. 16 (4.5%) (*P* >0.2) | 0 | 0 |
| Dexter  2001[74]  USA | (primary outcomes-"the rates at which the various preventive therapies were ordered") 1. Proportion of hospitalizations with an order for therapy **1a. Prophylactic heparin 1b. Prophylactic aspirin at discharge**  2 Proportion of hospitalizations during which therapy was ordered for an eligible patient **2a. Prophylactic heparin 2b. Prophylactic aspirin at discharge** | 1a. 10.5% vs. 8.2%, *P*<.001 1b. 29.7% vs. 25.4%, *P*=.005  2a. 32.2% vs. 18.9%, *P*<.001 2b. 36.4% vs. 27.6%, *P*<.001 | ... | ... | + | … |
| McCowan  2001[75]  UK | N = 147 vs. 330 patients; 6 month follow-up Main outcomes  **1. Prescriptions for acute asthma exacerbations; number (proportion) of patients; OR (95% CI). 1a. Received oral corticosteroids. 1b. Received emergency nebulisations**  Prespecified  2. Number (proportion) of patients for whom each of the British asthma guidelines steps was taken for maintenance prescribing (analysis not provided) 2a. step 0 2b. step 1 2c. step 2 2d. step 3 2e. step 4 2f. step 5 | 1a.. 7 (5%) vs. 35 (11%); 0.42 (0.14 to 1.29) 1b. 1 (1%) vs. 17 (5%); 0.13 (0.01 to 0.91)  2. *P*=.51 for trend across 2a-2f  2a. 53 (36%) vs. 116 (35%) 2b. 20 (14%) vs. 45 (15%) 2c. 50 (34%) vs. 127 (38%) 2d. 6 (4%) vs. 21 (6%) 2e. 18 (12%) vs. 15 (5%) 2f. 0% vs. 3 (1%) | N=147 vs. 330 patients; 6 month follow-up.  **1. Number (proportion) of patients with acute exacerbation of asthma; OR (95% CI) (primary)**  2. Number (proportion) of patients with hospital contacts for asthma; OR (95% CI) (prespecified) 2a. Admissions. 2b. Accident and emergency. 2c. Outpatients. | 1. 12 (8%) vs. 57 (17%); 0.43 (0.21 to 0.85)  2a. 0% vs. 4 (1%); 0 (0 to 3.44) 2b. 0% vs. 2 (1%); 0 (0 to 9.16) 2c. 2 (1%) vs. 7 (2%); 0.64 (0.09 to 3.38) | + | + |
| Demakis  2000[76]  USA | Components of primary outcomes 1. Proportion of patients in compliance with all 13 standards of care over 17 months. N, % adherent; OR (95% CI). **1a. Warfarin treatment monitoring. 1b. Atrial fibrillation: warfarin, aspirin, or ticlopidine. 1c. Myocardial infarction:** β**-blocker.** **1d. Gastrointestinal bleeding/NSAID therapy: switch drugs.   2. Proportion of all visits for which care was indicated and residents provided proper care over 17 months. N, % adherent; OR (95% CI). 2a. Warfarin treatment: monitoring.  2b. Atrial fibrillation: warfarin, aspirin, or ticlopidine.** **2c. Myocardial infarction:** β**-blocker.** **2d. Gastrointestinal bleeding/NSAID therapy: switch drugs.** | 1a. 287, 67.3% vs. 276, 64.5%; 1.13 (0.68 to 1.88, *P*=.63) 1b. 236, 75.0% vs. 241, 81.7%; 0.67 (0.41 to 1.09), *P*=.10 1c. 275, 44.7% vs. 334, 41.3%; 1.15 (0.81 to 1.62, *P*=.42) 1d. 490, 65.5% vs. 474, 67.9%; 0.90 (0.65 to 1.23, *P*=.49)   2a. 105, 32.4% vs. 122, 42.6%; 0.64 (0.36 to 1.15, *P*=.13) 2b. 62, 54.8% vs. 66, 53.0%; 1.08 (0.51 to 2.28, *P*=.85) 2c. 150, 18.0% vs. 189, 18.0%; 1.00 (0.54 to 1.85, *P*>.99) 2d. 145, 24.8% vs. 113, 31.0%; 0.74 (0.40 to 1.34, *P*=.31) | ... | ... | 0 | … |
| Hetlevik  1999[77-79]  Norway | … | … | All prespecified  For hypertension patients at 18 mo (total N = 2239) 1. Mean (SD) and change for SBP (mm Hg) in last 12 mo (n=1727).  2. Mean (SD) and change for DBP (mm Hg) in last 12 mo (n=1727).  3. Mean (SD) and change for serum cholesterol (mmol/L) in last 12 mo (n=821).  4. Mean (SD) and change for BMI (kg/m2) in last 18 mo (n=286).  5. Proportion and change in proportion of smokers at 18 mo (n=297).  6. Mean (SD) and change in CHD risk score at 18 months  6a. Women (n=89).  6b. Men (n=76).   7. Proportion and change in proportion of patients with cardiovascular inheritance at 18 mo (n=482).   **For hypertension patients at 21 mo (after feedback on missing data at 18 mo). 8. Mean (SD) and change for SBP (mm Hg) (n=1839).  9. Mean (SD) and change for DBP (mm Hg) (n=1839).  10. Mean (SD) and change for serum cholesterol (mmol/L) (n=1349).  11. Mean (SD) and change for BMI (kg/m2) (n=1053).  12. Proportion and change in proportion of smokers (n=1160).  13. Mean (SD) and change in CHD risk score 13a. Women (n=500).  13b. Men (n=391).  14. Proportion and change in proportion of patients with cardiovascular inheritance (n=1235).**   Note: Mean (SD) SBP higher in CCDSS group at baseline. 159.1 (20.3) vs. 156.4 (19.7), difference 2.7 (1.0 to 4.5).  Prespecified For diabetic patients at 18 mo (total N = 1034) 15. Mean (SD) and change (95% CI) for SBP (mm Hg) in last 12 mo (n=648).  16. Mean (SD) and change for DBP (mm Hg) in last 12 mo (n=648).  17. Mean (SD) and change for serum cholesterol (mmol/L) in last 12 mo (n=321).  18. Mean (SD) and change for BMI (kg/m2) in last 18 mo (n=112).  19. Proportion and change in proportion of smokers at 18 mo (n=89).  20. Mean (SD) and change in CHD risk score at 18 mo  20a. Women (n=19).  20b. Men (n=22).  21. Proportion and change in proportion of patients with cardiovascular inheritance at 18 mo (n=150).   22. Mean (SD) and change in HbA1c level in last 12 mo (n=640).   **For diabetic patients at 21 mo (after feedback on missing data at 18 mo) 23. Mean (SD) and change (95% CI) for SBP (mm Hg) (n=697).  24. Mean (SD) and change for DBP (mm Hg) (n=697).  25. Mean (SD) and change for serum cholesterol (mmol/L) (n=535).  26. Mean (SD) and change for BMI (kg/m2) (n=427).  27. Proportion and change in proportion of smokers (n=460).  28. Mean (SD) and change in CHD risk score 28a. Women (n=184).  28b. Men (n=142).  29. Proportion and change in proportion of patients with cardiovascular inheritance (n=452).  30. Mean (SD) and change in HbA1c level (n=689).**   After 18 mo, CCDSS had been used in treatment of 104 hypertension patients (12%) and 52 of diabetic patients (14%). | 1. 156.7 (19.5) vs. 155.5 (18.7), 1.2 (-0.6 to 3.0) 2. 88.6 (9.7) vs. 89.6 (8.8), -1.0 (-1.9 to -0.2) 3. 6.6 (1.2) vs. 6.7 (1.3), -0.1 (-0.3 to 0.1) 4. 28.9 (4.3) vs. 28.6 (4.9), 0.3 (-0.9 to 1.3) 5. 23% vs. 29%, -6 (-16 to 4) 6a. 18.3 (19.8) vs. 25.2 (24.2), -6.9 (-16.3 to 2.5) 6b. 56.0 (42.0) vs. 65.1 (83.4), -9.1 (-40.7 to 22.6) 7. 76% vs. 89%, -13.0 (-20.1 to 5.9) 8. 156.8 (19.4) vs. 155.6 (19.0), 1.2 (-0.6 to 3.0) 9. 88.8 (9.7) vs. 89.8 (8.9), -1.0 (-1.9 to -0.2) 10. 6.64 (1.2) vs. 6.57 (1.3), 0.07 (-0.1 to 0.2) 11. 27.8 (4.5) vs. 27.7 (4.8), 0.1 (-0.4 to 0.7) 12. 21% vs. 19%, 2.0 (-2.6 to 6.6) 13a. 17.9 (17.9) vs. 20.6 (23.5), -2.7 (-6.3 to 1.0) 13b. 67.9 (83.9) vs. 66.8 (73.4), 1.1 (-14.6 to 6.9) 14. 62% vs. 66%, -4.0 (-14.5 to 6.5) 15. 151.4 (22.2) vs. 153.7 (20.5), -2.3 (-5.6 to 1.0) 16. 82.8 (10.7) vs. 85.3 (9.9), -2.4 (-4.0 to -0.9) 17. 6.2 (1.5) vs. 6.3 (1.2), -0.1 (-0.3 to 0.2) 18. 29.6 (5.0) vs. 29.8 (5.7), -0.2 (-2.4 to 2.0) 19. 23% vs. 30%, -7 (-28.3 to 14.3) 20a. 30.2 (32.8) vs. 12.5 (9.3), 17.7 (-18.0 to 53.4)  20b. 39.8 (33.9) vs. 68.7 (83.4), -28.9 (-229.1 to 171.3) 21. 84% vs. 94%, -10.0 (-19.8 to -0.3) 22. 7.9 (1.6) vs. 8.0 (1.6), -0.1 (-0.4 to 0.1)  23. 151.5 (22.1) vs. 152.7 (19.0), -1.2 (-4.4 to 2.0) 24. 82.8 (10.6) vs. 85.1 (10.1), -2.3 (-3.8 to -0.8) 25. 6.2 (1.3) vs. 6.2 (1.3), 0 26. 28.6 (5.1) vs. 28.3 (6.3), 0.3 (-0.8 to 1.4) 27. 19% vs. 16%, 3.0 (-4.0 to 10.0) 28a. 14.3 (17.7) vs. 14.2 (17.5), 0.1 (-5.1 to 5.2)  28b. 51.4 (53.5) vs. 48.7 (44.1), 2.6 (-14.2 to 19.5) 29. 66% vs. 63%, 3.0 (-5.8 to 11.8) 30. 7.8 (1.6) vs. 7.9 (1.6), -0.1 (-0.4 to 0.1) | … | 0 |
| Overhage  1997[80]  USA | Prespecified unless otherwise indicated: **1. Number of times pharmacists intervened with physicians for significant errors over 6 months.**   Not prespecified  2. Compliance with corollary orders within 24 hours for the following 25 most common triggering orders. Total number of orders; % compliance (% increase).  2a. Heparin infusion 2b. cimetidine po 2c. Insulin lente humulin 2d. Furosemide po 2e. Ferrous sulphate 2f. Furosemide IV 2g. Warfarin. 2h. Insulin NPH humulin 2i. Vancomycin IV 2j. Sustained release theophyllin 2k. Gentamicin IV 2l. Insulin reg humulin 2m. Digoxin po 2n. Glyburide po 2o. Meperidine IM/IV 2p. Captopril po 2q.Enalapril po 2r.Kayexalate suspension 2s.Timentin IV 2t.Spironolactone po 2u.Glipizide po  3. Compliance with the following 25 most common corollary orders within 24 hours. Total number of orders; % compliance (% increase).  3a. Theophylline level 3b. Diphenhydramine 3c. Acetominophen 3d. Vancomycin 3e. Phenytoin level 3f. Gentamicin level | 1. 105 vs. 156, *P*=.003  2a. 1476; 77.42% vs. 40.24% (37.18%) 2b. 1055; 12.66% vs. 5.18% (7.48%) 2c. 518; 40.00% vs. 31.01% (8.99%) 2d. 410; 75.38% vs. 62.09% (13.29%) 2e. 394; 21,43% vs. 16,47% (4.96%) 2f. 360; 60.88% vs. 51.85% (-0.98%) 2g. 303; 68,18% vs. 35.09% (33.09%) 2h. 241; 52.17% vs. 26.19% (25.98%) 2i. 224; 60.44% vs. 44.36% (16.08%) 2j. 215; 73.33% vs. 45.46% (27.88%) 2k. 197; 78.35% vs. 61.00% (17.35%) 2l. 197; 53.33% vs. 35.87% (17.46%) 2m. 178; 96.88% vs. 84.15% (12.73%) 2n. 177; 51.28% vs. 43.43% (7.85%) 2o. 177; 24.24% vs. 5.41% (18.84%) 2p. 177; 74.42% vs. 55.06% (19.36%) 2q. 161; 73.68% vs. 70.59% (3.10%) 2r. 161; 26.09% vs. 18.48% (7.61%) Article reports difference % as 18.48 (repeat of control group %) – revised to 7.61% - could not confirm with author (no response).  2s. 161; 45.24% vs. 14.29% (30.95%) 2t. 158; 42.25% vs. 20.69% (21.56%) 2u. 147; 47.22% vs. 36.00% (11.22%)  3a. 270; 75.89% vs. 46.51% (29.38%) 3b. 267; 16.41% vs. 7.19% (9.21%) 3c. 232; 19.66% vs. 14.78% (4.88%) 3d. 143; 90.74% vs. 65.17% (25.57%)  3e. 140; 73.13% vs. 38.36% (34.78%) 3f. 118; 90% vs. 75.86% (14.14%) | Not clearly prespecified **1. Mean hospital length of stay (days).  2. Maximum serum creatinine level during hospital stay (units not reported).** | 1. 7.62 vs. 8.12 (difference -0.5, 95% CI -0.17 to 1.19, *P*=.94) 2. 1.51 (1.25) vs. 1.42 (0.88), *P*=.28 | + | 0 |
| Overhage  1996[82]  USA | **Components of primary outcomes 1. Compliance with preventive care guidelines over 6 months: No. of eligible patients (% compliance). 1a. Aspirin. 1b. Oestrogen treatment. 1fc Calcium treatment..**  **1d. ACE-I.**  **1e. Heparin prophylaxis. 1f. β-blocker.** | 1a. 246 (9.4%) vs. 247 (9.7%), *P*=.89 1b. 243 (0.8%) vs. 232 0.3%), *P*=.62 1c. 243 (5.4%) vs. 232 (3.9%), *P*=.45  1d. 35 (29.0%) vs. 45 (56.0%), *P*=.02  1e. 30 (43.3%) vs. 28 (35.7%), *P*=.55 1f. 14 (14.3%) vs. 10 (20.0%), *P*=.71 | ... | ... | 0 | … |
| Tierney  1993[84]  USA | … | … | **Predefined. 1. Mean (SE) / median length of hospital stay (days, % reduction).  2. Resources used 1 and 3 months after discharge (limited data). 2a. Number of primary care visits.**  **2b. Number of emergency department visits.  2c. Number of outpatient visits.  2d. Number of hospital readmissions.** | 1. 7.60 (0.20) / 5 vs. 8.49 (0.24) / 6, 10.5%, *P*=.11 2a. *P*>.20 2b. *P*>.20 2c. *P*>.20 2d. *P*>.20 | … | 0 |
| Mazzuca  1990[85]  USA | Pre-specified. 3 treatment groups (B - CCDSS reminder + seminar; C = B + seminar-related clinical materials; D = C + diabetes patient education service) vs. control (seminar only).   1. Adherence to 5 recommendations for care of non-insulin dependent diabetes (11 months follow up): number of physicians/number of eligible patients; mean (SE) for **B vs.** C vs. D vs. **A. 1a. Initiation of oral hypoglycaemic therapy.** | 1a. 99/292; 0.24 (0.07) vs. 0.26 (0.06) vs. 0.31 (0.07) vs. 0.20 (0.06); *P*=NS overall | ... | ... | 0 | … |
| McAlister  1986[86]  Canada | No outcomes clearly prespecified  1. Mean % of patients treated for hypertension (95% CI) (16 Month Follow Up) **a.All patients** b.Moderate hypertension c.Mild hypertension d.Newly diagnosed  All patients: baseline DBP > 90 mmHg or prescribed antihypertensive medication. Moderate hypertension: baseline DBP >104 mmHg Mild hypertension: baseline DBP >90 to <105 mmHg | 1a.95.4 (87.1-100*) vs. 95.7 (87.7 -100) ; NS 1b.95.1 ( 86.6-100) vs. 84.5 (70.3-98.7) ; NS 1c.91.4 (80.4-100) vs. 90.2 (78.5-100) ; NS 1d.79.4 (63.5-95.3) vs. 76.1 (59.4 -92.8) ; NS  *Upper 95% CI truncated at 100% | No outcomes clearly prespecified  1.Mean % of patients with diastolic pressure ≤90 mmHg on last visit at 16 Months (95% CI) **1a.All patients** 1b.Moderate hypertension 1c.Mild hypertension 1d.Newly diagnosed  2.Mean no. of days with diastolic pressure ≤90 mmHg per patient-year at 16 months (95% CI) **2a.All patients** 2b.Moderate hypertension 2c.Mild hypertension 2d.Newly diagnosed  3.Mean change in median diastolic pressure (mmHg) from baseline to last visit (95% CI) **3a.All patients** 3b.Moderate hypertension 3c.Mild hypertension 3d.Newly diagnosed | 1a. 88.9 (76.5-100) vs. 87.5 (74.5-100); NS 1b. 86.0 (72.4-99.6) vs. 76.2 (59.5-92.9); NS 1c. 87.9 (75.1-100) vs. 88.3 (75.7-100); NS 1d. 92.4 (82.0-100) vs. 91.5 (80.6-100); NS  2a. 215.6 (175.1-256.1) vs. 202.6 (160.8-244.4); NS 2b. 191.7 (136.6-246.8) vs. 175.7 (119.1-232.3); NS 2c. 251.0 (205.7-296.3) vs. 274.0 (229.5-318.5); NS 2d. 323.2 (299.7-346.7) vs. 258.5 (212.8-304.2); P<.03  3a. -4.9 (-6.6 to -3.2) vs. -4.1 (-6.1 to -2.1); NS 3b. -21.7 (-25.1 to -18.3) vs. -16.7 (-19.9 to -13.5); P<.06 3c. -9.8 (-11.9 to -7.7) vs. -8.5 (-10.8 to -6.2); NS 3d. -15.1 (-18.2 to -12.0) vs. -11.3 (14.2 to -8.4); NS | 0 | 0 |

Abbreviations: ACE-I, angiotensin converting enzyme inhibitor; ALT, alanine aminotransferase; AMI, acute myocardial infarction; ARB, angiotensin receptor blocker; BP, blood pressure; CCDSS, computerized clinical decision support system; CHD, coronary heart disease; CI, confidence interval; COPD, chronic obstructive pulmonary disease; CVD, cardiovascular disease; DBP, diastolic blood pressure; ED, emergency department; EMR, electronic medical record; ESR, erythrocyte sedimentation rate; GP, general practitioner; IQR, interquartile range; ITT, intention to treat; LDL, low-density lipoprotein; LLD, lipid lowering drug; LVEF, left ventricular ejection fraction; NR, not reported; NRT, nicotine replacement therapy; NS, not significant; NSAID, non-steroidal anti-inflammatory drug; OR, odds ratio; po, per os (by mouth); PTCA, percutaneous transluminal coronary angioplasty; RR, relative risk; SBP, systolic blood pressure; SD, standard deviation; SE, standard error; SEM, standard error of the mean; TPI, thrombolytic predictive instrument; VAS, visual analog scale. Outcomes in bold font were assessed for effect..

^a^Outcomes are evaluated for effect as positive (+) or negative (−) for CCDSS, or no effect (0), based on the following hierarchy. An effect is defined as ≥ 50% of relevant outcomes showing a statistically significant difference (2*P*<.05)

- If a single primary outcome is reported, *in which all components are applicable*, this is the only outcome evaluated.
- If > 1 primary outcome is reported, the ≥ 50% rule applies and only the primary outcomes are evaluated.
- If no primary outcomes are reported (or only some of the primary outcome components are relevant) but overall analyses are provided, the overall analyses are evaluated as primary outcomes. Subgroup analyses are not considered.
- If no primary outcomes or overall analyses are reported, or only some components of the primary outcome are relevant for the application, any reported prespecified outcomes are evaluated.
- If no clearly prespecified outcomes are reported, any available outcomes are considered.
- If statistical comparisons are not reported and data are insufficient to conduct analyses, ‘effect’ is designated as not evaluated (…).

^b^Calculated based on data in article.
