# Supplementary material for: Computerized clinical decision support systems for drug prescribing and management: A decision-maker-researcher partnership systematic review
Source: Implement Sci. 2011 Aug 3;6:89. doi: 10.1186/1748-5908-6-89 (PMC3179735; doi:10.1186/1748-5908-6-89)
Supplement: Additional file 5 — Costs and CCDSS process-related outcomes for drug prescribing. Cost and CCDSS process-related outcomes for the included studies. [file 1748-5908-6-89-S5.DOCX]

**Additional file 5, Table S5. Costs and CCDSS process-related outcomes for trials of drug prescribing**

| **Study** | **CCDSS adverse effects** | **Costs^b^** | **Group comparison for CCDSS workflow** | **Practitioner satisfaction with CCDSS system** |
| --- | --- | --- | --- | --- |
| **Studies of drug-only interventions** | | | | |
| Field, 2009[17, 24] | … | Cost of developing and implementing system (through 2 weeks after system went live) including 94 alerts for 62 drugs were reported in a supplementary publication; cost of on-going maintenance and upgrades was not.  Estimated costs (based on reported hours and estimated hourly wages):  1. Costs by personnel category; hours; cost (US $), % of total time:  a. Pharmacist (Master’s level); 12 hours; $5,307.60; 13%  b. Pharmacist (Bachelor’s level); 59.75 hours; $1,814.01; 6%  c. Physician; 414.25 hours; $25,902.04; 45%  d. Project coordinator; 79.75 hours; $1,315.08; 9%  e. Health services researcher; 18.5 hours; $529.10; 2%  f. Informatics project manager; 121.75 hours; $4,987.27; 13%  g. Computer programmer; 110.5 hours; $8,813.48; 12%  h. Total 924.5 hours; $48,668.57  2. Costs by activity category; hours; cost (US $), % of total cost:  a. Project management; 80.25 hours; $2,220.17; 5%  b. CCDSS content preparation; 482.25 hours; $27,455.61; 56%  c. Informatics project management; 121.7 hours; $4,987.27; 10%  d. Blueprint/instruction preparation for programmer; 50.8 hours; $1,869.95; 4%  e. Programming; 110.5 hours; $8,813.48; 18%  f. Testing and implementing; 79.0 hours; $3,322.09; 7%  g. Total 924.5 hours; $48,668.57 | … | … |
| Fortuna, 2009[18] | ... | ... | ... | 51 (57%) of 89 clinicians who received alerts responded to a survey on attitudes to alerts.  A majority of clinicians agreed with the following alert benefits (n, %):  1. Provided useful evidence supporting prescribing decisions. 43 (88%)  2. Provided useful patient education information on insomnia. 40 (83%)  3. Increased clinician awareness of costs of hypnotic drugs. 35 (71%)  4. Didn't interfere with workflow. 35 (70%)  A minority of clinicians agreed with the following:  1. Prompted clinicians to spend more time discussing alternative treatments with patients. 24 (47%)  2. Changed prescribing decisions. 11 (23%) |
| Reeve, 2008[30] | ... | ... | ... | All pharmacists (intervention and control groups) were asked to respond to the following items; Median (score range, 1, strongly disagree, 10, strongly agree).  1. My initial reaction to the pop-up was that it was an intrusion: 2  2. I ignored the pop-up as it was of low importance to me: 3  3. The message the pop-up attempted to convey was clear and easy to understand: 7  4. I thought the Pharmacist Information was well-presented, useful and easy to understand: 7  5. I was concerned with the accuracy and evidence base of the information presented: 2  6. I considered the information in the Patient Information Leaflet useful for the patient: 7  7. I am comfortable to contact a doctor on a patient’s behalf to discuss aspirin prophylaxis: 6  8. The pop-up message & associated material increased my knowledge about aspirin & diabetes: 7  9. These types of pop-ups are useful: 7 |
| Heidenreich, 2007[33] | ... | … | ... | 85% of responding practitioners (35/41) thought reminders should be continued, 5% thought they should be discontinued, and 10% had no opinion. |
| Raebel, 2007b[36] | The study was stopped after 4 of 12 planned months because of a high rate of false alerts. 2 common false positive alerts were for medications not contraindicated (298 of 763 alerts, 39.1%) and for women that were not pregnant (347 false-positive alerts). The former was a function of software limitations; the latter a function of delays in updating the clinical databases. | ... | ... | ... |
| Verstappen, 2007[39] | ... | ... | ... | Author reports that majority of practitioners were satisfied with system, as indicated in questionnaire. |
| Kattan, 2006[43] | ... | Estimated cost of the intervention was $69.20 per child over the year. Total cost savings was $337.00 per child in the intervention group. Monte Carlo simulations, using the observed distributions of symptom days and resource use, showed that the intervention had a 97% chance of being cost saving. Hourly wage of $15 for a clerical employee was used in the calculation. There were 6 calls per child per year (40 mins per call) resulting in a cost of $60. The cost for these materials on a per child basis was $9.20. | ... | ... |
| Paul, 2006[48] | … | 1. Cost of antibiotic treatment for all 2326 patients (intervention vs. control) in Israel; Germany; Italy; overall n (%) p value:  a. direct cost in Euros (€), mean (SD)/patient.  25.2(33.2) vs. 25.5(30.9), *P* = .079; 68.9(75.6) vs. 73.5(85.4), *P* = .674; 79.1(87.7) vs. 84.9(83.9), *P* = .302; 37.9(54.2) vs. 40.2(57.6) *P* = .473  b.observed side effect cost in Euros, mean (SD)/patient.  98.3(1048.6) vs. 88.5(1046.9), *P* = .163; 129.2 (1294.4) vs. 189.8 (1765.5), *P* = .526;  74.6 (992.2) vs. 24.4 (159.2), *P* = .819; 100.1(1085.1) vs. 99.5 (1154.0), *P* = .960 c. Ecological costs in Euros, mean (SD)/ patient.  445.(404.7) vs. 511.7(439.9), *P* < .001; 517.8(374.6) vs. 503.8 (336.7), *P* = .870; 317.2 (282.2) vs. 372.2(248.3), *P* = .030; 439.5(388.4) vs. 499.3(414.1), *P* = .002  d. total antibiotic cost in Euros, mean (SD)/patient.  546.0(476.7) vs. 612.5(507.7), *P* = .001; 712.1(532.6) vs. 716.1(522.1), *P* = .960; 487.5(419.5) vs. 540.0(371.5), *P* = .135; 565.5(483.4) vs. 623.2(502.2), *P* = .007  (Note: See article appendix 2 for cost analysis calculations) | ... | ... |
| Weir, 2003[67] | ... | ... | ... | 9/9 respondents to survey indicated that CCDSS information was available quickly enough to be useful for prescribing decision.  8/9 respondents indicated that format for evidence was acceptable.  3/9 respondents disagreed with CCDSS.  5/9 respondents believed that CCDSS had influenced their prescribing. |
| Zanetti, 2003[68] | 1, Inappropriate activation of the system, n, %. 4/449 procedures (1%).  2. Unnecessary intraoperative redosing, n=1. | ... | ... | ... |
| Rotman, 1996[83] | ... | Primary outcome: Mean drug cost per physician per day (NS between treatments). | ... | 5-point Likert scale for user satisfaction (1, strongly disagree; 5, strongly agree). CCDSS vs. control, mean (SD). During intervention: 2.98 (0.547) vs. 3.72 (0.333)  Change from preintervention: -0.343 (0.112) vs. 0.488 (0.084 Difference between groups in change from preintervention: 0.831 (0.138), *P* < .0001 |
| McDonald, 1976[89] | There was a problem with the recommendations for renal treatment-in some cases the computer suggested unwarranted reductions in medication. Practitioners did not act on the erroneous recommendations. | ... | ... | ... |
| **Studies of multi-faceted interventions** | | | | |
| Bertoni, 2009[16, 21] | Patients had a greater risk for overtreatment than of undertreatment because all patients were screened, including low risk patients who would not normally be screened. | ... | ... | ... |
| Gilutz, 2009[19] | Author comment: no adverse effects. | Author comment: yes it was cost effective; data not stated. The cost of implementing the CCDSS was $170000. | ... | Author commented that 143 satisfaction grading forms were available with 91% rate of general satisfaction. |
| Javitt, 2008[27] | ... | Outcomes over 1 year.  1. Change in costs $ of medical care from previous year relative to control group; $ (t-statistic).p-value  a. Total charges (per member per month [pmpm]). −21.92 (1.99); *P* <.05 (6.1% reduction in mean total charges from mean control group charges of $352 pmpm).  b. In-patient charges (pmpm). −12.833 (1.8); *P* = .1  c. Out-patient charges (pmpm). −1.823 (0.60); NS  d. Medication charges (pmpm). 0.7 (0.90); NS  e. Professional charges (pmpm). −7.963 (2.20); *P* < .05  f. In hospital (pmpm). 0.000 (0.10); NS  2. Mean reimbursement differentials (hospital charged health maintenance organization for all services but was reimbursed at a fixed rate): -$8.96 overall (6% drop in CCDSS group), mostly due to professional reimbursements (-$4.62) and mean in-patient charges (-$3.88).  3. Charge differentials:  3a. Patients at the median. $0.561 pmpm  3b. Patients at the 90th percentile. -$26.512 pmpm  3c. Patients at the 99th percentile. -$658.612 pmpm  4. Change in costs $ of medical care in subgroup of patients > 50 years of age at baseline (intervention vs. control); $ (t-statistic).p-value  a. Total charges (per member per month [pmpm]). -$72.171 (2.04)  b. In-patient charges (pmpm). -$49.633 (2.12)c. Out-patient charges (pmpm). -$9.59 (1.10)  d. Medication charges (pmpm). $0.447 (0.13)  e. Professional charges (pmpm). -$13.395 (1.18)  5. Change in costs $ of medical care in all patients (weighted by propensity score for risk of receiving a care consideration based on pre-study characteristics), (intervention vs. control); $ (t-statistic).p-value  a. Total charges (per member per month [pmpm]). -$66.363 (3.44)  b. In-patient charges (pmpm). -$38.067 (3.5)c. Out-patient charges (pmpm). -$2.913 (0.57)d. Medication charges (pmpm). -$0.821 (0.46)e. Professional charges (pmpm). -$24.562 (2.86) | ... | ... |
| Quinn, 2008[29] | ... | ... | ... | All physicians reported that they would recommend the WellDoc System to other patients and that it was easy to use, facilitated treatment decisions, and would improve efficiency of office visits. |
| Lester, 2006[45, 59] | ... | Median time to complete an email notification: 90 sec (range 15 sec to 49 min). | ... | ... |
| Cobos, 2005[49] | ... | Direct costs, sum of costs of physician visits, laboratory analyses, and lipid lowering drugs prescribed during the study. For each patient, visit and laboratory costs were estimated by frequency x unit cost (physician visit Euro 12, lipid Euro 9.46, alanine aminotransferase and aspartate amintransferase Euro 2 each, creatine kinase; Euro 1). All costs in Euros for CCDSS vs. control.  1. LLD treatment costs at 1 year. 125,569 vs. 214,683  2. LLD total costs at 1 year. 170,061 vs. 264,658  3. Adjusted means for treatment costs per patient; difference (95% CI); savings %. 178 vs. 237; 59 (34 to 83, p<0.00001); 24.9%.  4. Adjusted means for total costs per patient; difference (95% CI); savings %. 223 vs. 283; 60 (33 to 86), p=0.001; 20.8% | ... | ... |
| Javitt, 2005[52] | ... | Study reported payer costs but not patient or caregiver costs. Intervention costs were $1 to $1.50 per plan member per month (pmpm) with an overall return on investment of $8.07 pmpm.  Other cost outcomes for N, 19,739 vs. 19,723 patients. 1. Total inpatient charges per patient per month over 12 months; difference in $. $58.95 vs. $68.36; -$9.41 (*P=* 0.001). 2. Total inpatient paid claims per patient per month over 12 months; difference in $. $26.06 vs. $28.20; -$2.14 (*P =* 0.008). 3. Overall difference ($ per patient per month) in charges over 12 months (95% CI). -$18.62 (-$12 to -$25), p-value not reported. 4. Overall difference ($ per patient per month) in paid claims over 12 months (95% CI) (primary). -$8.07 (-$5 to -$11), p-value not reported. Subgroup analyses of patients who triggered recommendations (both interventions [n=961] and control [n=982]): 5. Total inpatient charges per patient per month over 12 months; difference in $. $242.30 vs. $296.30; -$54.00 (*P* = .007). 6. Total inpatient paid claims per patient per month over 12 months; difference in $. $93.50 vs. $127.50; -$34.00 (*P* = .006). 7. Overall difference ($ per patient per month) in charges over 12 months (95% CI). -$77.91 (-$26 to -$130) (*P* =.003). 8. Overall difference ($ per patient per month) in paid claims over 12 months (95% CI). -$68.08 (-$39 to -$98) (*P* =.003). 9. Difference in charges/paid claims over 12 months by type of intervention recommended. 9a. Add a drug. Higher in control group (data shown only in figure 2, $300-350/approx. $200) 9b. Stop a drug. Higher in control group (data in figure 2, $100-$150/$50-$100 9c. Get a test. NS (data in figure 2, 0-$50/0 to -$50 Note: Figures (but no data) in article show claims costs separately for inpatients, outpatients, professional, pharmacy, and total – both overall and for subgroup of patients who triggered recommendations.  10. Difference in charges/paid claims over 12 months for subgroup of patients who did not trigger care considerations. (Not significant). Subgroup analyses for patients with HOPE trial-consistent recommendation for ACE-I prescription (n=156 vs. 155 patients). 11. Inpatient charges per person over 12 months, mean. $5,835 vs. $8,746, *P* = .05 12. Non-HOPE-related hospital inpatient charges per person over 12 months, mean. $6,704 vs. $8,416, *P* = .30 | … | ... |
| Plaza, 2005[53] | ... | Direct and indirect costs were calculated. The former was calculated as the product of the consumption of each resource times its unitary cost. Treatment costs were estimated using drug market prices in Spain. The remaining costs were obtained through the SOIKOS database with prices corrected for year 2001 and using average prices (medical visits: 8.47 €; home visits:19.53 €; emergency visits: 87.53 €; days in intensive care unit: 1,156.44 €; shift days: 282.46 €; espirometries: 10.03 €; blood standard analysis: 10,73 €; total E immunoglobulin: 6.77 €, thorax radiographies: 14.35 €; skin allergic tests: 30.05 €).  Indirect costs were calculated, for active workers, as the product of hours lost and the total cost per hour of effective work (11.79 €). The total costs were estimated using a social and national healthcare system perspective.  From a social perspective the medical costs per patient rose to 2444.35 (987 treatments) in the usual care group and 1408 (833 treatments) in group 1.  The estimated difference was -1022 (95%CI -2165 to 122; *P*=.08).  From the perspective of the National Health Care System, the total median medical cost per patient rose 1544 (891 treatments) in the usual care group and to 1077 (767 treatments) in group 1. The difference was -489 (95%CI -1302 to 324; *P*=.24). | ... | ... |
| Sequist, 2005[55] | ... | ... | ... | 71% of physicians preferred the electronic decision support over a paper-based system and 76% thought that the system helped to improve quality of care. Of the physicians in the intervention group, 68% found electronic reminders for diabetes care useful; 53% found them useful for coronary artery disease management. |
| Tierney, 2005[56] | ... | Physician intervention vs. pharmacist intervention vs. both interventions vs. control 1. Mean (SD) direct health care charges over 12 months (US $). All *P* =NS unless noted otherwise.  a. Outpatient charges. 3,142 (3,381) vs. 2,814 (3,282) vs. 3,177 (3,558) vs. 3,129 (2,921)  b. Inpatient charges. 4,864 (17,257) vs. 2,519 (7,267) vs. 2,475 (8,699) vs. 2,671 (6,805)  c. Total health care charges. 8,006 (18,720) vs. 5,333 (9,400) vs. 5,652 (10,579) vs. 5,800 (8,536), p<0.05 for increase with physician intervention. | ... | ... |
| Wolfenden, 2005[57] | ... | Trial cost/Estimated annual cost of ongoing care delivery (Australian $)  1. Clinical practice change strategies.  1a. Staff time – opinion leaders. 886/0  1b. Staff time – consensus processes. 1799/0  1c. Staff time – training.1086/522  1d. Staff time – performance feedback. 1234/821  1e. Staff time – project staff. 15,866/3225  1f. Goods – computer hardware. 3300/0  1g. Services – software development. 10,000/0  1h. Subtotal. 34,171/4568  2. Delivery of care elements. [To confirm with author – seems incorrectly offset]  2a. Staff time – referral to computer, brief advice, NRTprovision/prescription. 281/562 2b. Goods. 445/1512  2c. NRT. 377/1232  2d. Computer stock. 2346/7590 2e. Services. 20/80  2f. Computer maintenance. 80/175  2g. Subtotal. 3549/11,151.  3. Total costs. 37,720/15,719 | ... | All aspects of care delivery highly acceptable to 5 nurses and 10 anaesthetists (n, % acceptable):  a. Quit advice and assistance from a touch-screen computer program. 12/15, 80% b. Computer print-outs were helpful when providing advice and assistance. 12/15, 80%  c. Was not difficult to find time to offer preoperative NRT. 13/15, 87%  d. Completing or approving postoperative NRT prescriptions integrated well with existing clinical procedures. 14/15, 93%  e. Intervention is appropriate for other preoperative clinics. 15/15, 100% |
| Murray, 2004[60] | ... | Physician intervention vs. pharmacist intervention vs. both vs. neither.  1. % direct health care charges ± SD  a. Outpatient. 3005 ± 4318 vs. 2868 ± 3553 vs. 2681 ± 3520 vs. 2229 ± 2137; NS  b. Inpatient. 2145 ± 9805 vs. 2577 ± 7709 vs. 3519 ± 17830 vs. 893 ± 3450; NS  c. Total health care charges. 5149 ± 11756 vs. 5445 ± 9612 vs. 6200 ± 18947 vs. 3122 ± 4633; NS | ... | ... |
| Tierney, 2003[66] | …. | Mean (SD) direct health care charges per patient over 1 year: physician intervention vs. pharmacist intervention vs. both interventions vs. control.  a. Outpatient charges. $2,961 (2,795) vs. $3,143 (3,124) vs. $3,176 (2,974) vs. $2,696 (3,363)  b. Inpatient charges. $3,341 (9,162) vs. $4,245 (11,544) vs. $4,463 (15,217) vs. $4,330 (14,461)  c. Total charges. $6,302 (10,928) vs. $7,387 (13,206) vs. $7,639 (16,921) vs. $7,025 (17,024) | ... | ... |
| Eccles, 2002[64, 69] | ... | ... | ... | Author comment: We have always interpreted Figure 3 (shows the number of times the guidelines were triggered for each practice and the proportion of active interactions that involved going beyond the first screen - the median number of active interactions was zero for much of the study) to mean that the majority of the users were not satisfied - in that they stopped using the system. This is certainly what the process evaluation suggested. |
| Flottorp, 2002[63, 70] | Experience with installation and use of software was reported in the supplementary paper. Practices provided group feedback on the following 4 items (5-point scale, 1, no problems, 5, very difficult).  1. Experience with software installation (n=112): mean 2.5 (median 2); 1, 35%; 2, 18%; 3, 23%; 4, 9%; 5, 13%; don't know, 3%  2. Experience using software (n=112): mean 2.1 (median 2); 1, 39%; 2, 29%; 3, 18%; 4, 8%; 5, 4%; don't know, 3%  3. Experience filling out 'pop-up' questionnaires (n=112): mean 1.9 (median 2); 1, 42%; 2, 30%; 3, 19%; 4, 5%; 5, 2%; don't know, 2%  4. Software worked technically ok (n=120): mean 2.1 (median 2); 1, 32%; 2, 36%; 3, 21%; 4, 11%; 5, 1%; don't know, 0% | ... | ... | Limited information on practitioner satisfaction with the CCDSS in this multicomponent intervention. Mean score of 2.8 (range 1, yes, 5, no) for 205 respondents on question "Was the computer-based advice helpful?" |
| McCowan, 2001[75] | ... | All 9 responders said the CCDSS could be used in consultations lasting <= 10 minutes, although users indicated it had slightly increased consultation times. | ... | Users found the software easy to use. Management recommendations and reminders were popular with users, who felt they contributed to improved quality of consultations. The risk prediction data was not popular within the consultation. Printed management plans were useful and of value to patients. |
| Hetlevik, 1999[77-79] | ... | ... | ... | 1. Physicians reported the CCDSS provided some or much benefit for checking blood pressure (68%); taking history (67%); diagnostic support, lab tests, lifestyle advice (oral), and infarction risk score calculation (61%); lifestyle advice (out-prints) (56%); clinical exams (53%), and treatment indications (50%).  2. Physician evaluation of CCDSS user-friendliness (agreed or partly agreed).  a. One CCDSS for 3 diagnoses as a good/acceptable solution (20/24, 83%)  b. CCDSS too large (22/24, 92%)  c. Recommended procedures too time-consuming (20/24, 83%)  d. Many recommendations were unnecessary (12/24, 50%)  e. Could remember procedures without the CCDSS (16/24, 67%)  3. Physicians reported some CCDSS implementation strategies were of some or large benefit:  a. Ready to use (11/21, 52%)  b. Physician training (17/21, 81%)  c. Assistant training (10/18, 56%)  d. Physician user manual (11/21, 52%)  e. Telephone repetitions for physicians (15/21, 71%)  f. Attending risk intervention seminar (2/2, 100%)  Strategies of little of no use:  g. Assistant user manual (10/19, 53%)  h. Report of own patients with diabetes (11/21, 52%)  i. Checking use of CCDSS (14/20, 70%)  j. Visiting the CCDSS stand (2/2), 100%). |
| Overhage, 1997[80] | ... | Mean hospital charges for intervention vs. control: $8,073.52 vs. $8,589.47 (difference -$515.95, 95% CI -828.41 to 1,316.85, p=0.68). | ... | ... |
| Tierney, 1993[84] | ... | Main outcome  1. Mean (SEM) /median charges per hospital admission ($ US), % change, (p value for mean).  1a. Overall. 6,077 (210) / 3669 vs. 6,964 (242) / 3984, 12.7%, *P* =.02  1b. Beds. 2,283 (69) / 1450 vs. 2,551 (81) / 1550, 11.9%, *P* = .04  1c. Diagnostic tests. 1,621 (58) / 992 vs1,852 (53) / 1143, 12.5%, *P* = .006  1d. Drugs. 1,001 (42) / 505 vs. 1,181 (47) / 546, 15.3%, *P* = .008  1e. Other. 1, 171 (63) / 541 vs. 1,381 (83) / 533, 15.2%, *P* = .81 2. Hospital costs (item charge x cost centre charge ratio), % reduction with CCDSS.  2a. Overall. 13.1%, *P* = .02  2b. Bed costs. 10.5%, *P* = .04  2c. Test costs. 12.4%, *P* = .005  2d. Drug costs. 15.1%, *P* = .008  2e. Other. 14.9%, *P* = .80  3. Total outpatient charges at 1 and 3 months after discharge. No data, *P* > .20. | Compared with no computerized ordering, workflow demands were greater.  1. Mean time writing orders between 10am and 8pm (minutes). 58.5 vs. 25.5, *P* < .001.  2. Mean increase in time for intervention group writing orders per patient between 10am and 8pm (minutes).  2a. Overall. 5.5  2b. Admitting. 10.6  2c. Discharge. 9.9 3. Mean decrease in time for intervention group routinely recording patient data (writing “scut” cards) (minutes). 5.7, *P* = .2 | Results of user survey (% physicians)  1. Workstation use increased order accuracy more than paper chart. 75%  2. Workstation use made work more interesting. 70%  3. Work done faster using workstation. 44%  4. Work easier using workstation. 52%  5. Workstation system too complex. 20%  6. Workstation use may result in “cookbook” medicine. 35%  7. Improvement in user opinion on workstation speed over 17 months. *P* = .04  8. Improvement in user opinion about workstation ease of use. *P* = .005 |

Abbreviations: CCDSS, computerized clinical decision support system; CI, confidence interval; LLD, lipid-lowering drug; NRT, nicotine replacement therapy; NS, not significant; SD, standard deviation; SEM, standard error of the mean; SGRQ, St. George’s Respiratory Questionnaire.

^a^ Ellipses (…) indicate outcome was not assessed.

^b^ Costs include workflow measures such as time to process alerts if these are not directly compared between groups.
